# Evaluation of global simulations of aerosol particle and cloud condensation nuclei number, with implications for cloud droplet formation

**DOI:** 10.5194/acp-19-8591-2019

**Published:** 2019-07-08

**Authors:** George S. Fanourgakis, Maria Kanakidou, Athanasios Nenes, Susanne E. Bauer, Tommi Bergman, Ken S. Carslaw, Alf Grini, Douglas S. Hamilton, Jill S. Johnson, Vlassis A. Karydis, Alf Kirkevåg, John K. Kodros, Ulrike Lohmann, Gan Luo, Risto Makkonen, Hitoshi Matsui, David Neubauer, Jeffrey R. Pierce, Julia Schmale, Philip Stier, Kostas Tsigaridis, Twan van Noije, Hailong Wang, Duncan Watson-Parris, Daniel M. Westervelt, Yang Yang, Masaru Yoshioka, Nikos Daskalakis, Stefano Decesari, Martin Gysel-Beer, Nikos Kalivitis, Xiaohong Liu, Natalie M. Mahowald, Stelios Myriokefalitakis, Roland Schrödner, Maria Sfakianaki, Alexandra P. Tsimpidi, Mingxuan Wu, Fangqun Yu

**Affiliations:** 1 Environmental Chemical Processes Laboratory, Department of Chemistry, University of Crete, Heraklion, 70013, Greece; 2 Laboratory of Atmospheric Processes and their Impacts, School of Architecture, Civil & Environmental Engineering, École Polytechnique Federale de Lausanne, Lausanne, 1015, Switzerland; 3 Institute of Chemical Engineering Sciences, Foundation for Research and Technology (FORTH/ICE-HT), Hellas, 26504, Patras, Greece; 4 NASA Goddard Institute for Space Studies, New York, NY, USA; 5 Center for Climate Systems Research, Columbia University, New York, NY, USA; 6 Royal Netherlands Meteorological Institute (KNMI), De Bilt, the Netherlands; 7 School of Earth and Environment, University of Leeds, UK; 8 independent researcher; 9 Department of Earth and Atmospheric Sciences, Atkinson Center for a Sustainable Future, Cornell University, Ithaca, NY, USA; 10 Department of Atmospheric Chemistry, Max Planck Institute for Chemistry, Mainz, Germany; 11 Forschungszentrum Jülich, Inst Energy & Climate Res IEK-8, 52425 Jülich, Germany; 12 Norwegian Meteorological Institute, Oslo, Norway; 13 Department of Atmospheric Science, Colorado State University, Fort Collins, Colorado, USA; 14 Institute for Atmospheric and Climate Science, ETH Zurich, Zurich, Switzerland; 15 Climate Atmospheric Sciences Research Center , of the State University of New York at Albany, Albany, 12203, New York, USA; 16 Climate System Research, Finnish Meteorological Institute, P.O. Box 503, 00101 Helsinki, Finland; 17 Institute for Atmospheric and Earth System Research/Physics, University of Helsinki, P.O. Box 64, 00014 Helsinki, Finland; 18 Graduate School of Environmental Studies, Nagoya University, Nagoya, Japan; 19 Laboratory of Atmospheric Chemistry, Paul Scherrer Institute, Villigen, Switzerland; 20 Atmospheric, Oceanic & Planetary Physics, Department of Physics, University of Oxford, Oxford OX1 2JD, UK; 21 Atmospheric Sciences and Global Change Division, Pacific Northwest National Laboratory, Richland, Washington, USA; 22 Lamont-Doherty Earth Observatory, Columbia University, Palisades, NY 10964, USA; 23 Laboratory for Modeling and Observation of the Earth System (LAMOS) Institute of Environmental Physics (IUP), University of Bremen, Bremen, Germany; 24 Institute of Atmospheric Sciences and Climate, National Research Council of Italy, Via Piero Gobetti, 101, 40129 Bologna, Italy; 25 Department of Atmospheric Science, University of Wyoming, Laramie, Wyoming, USA; 26 Institute for Environmental Research and Sustainable Development (IERSD), National Observatory of Athens, Penteli, Greece; 27 Centre for Environmental and Climate Research, Lund University, Lund, Sweden

## Abstract

A total of 16 global chemistry transport models and general circulation models have participated in this study; 14 models have been evaluated with regard to their ability to reproduce the near-surface observed number concentration of aerosol particles and cloud condensation nuclei (CCN), as well as derived cloud droplet number concentration (CDNC). Model results for the period 2011–2015 are compared with aerosol measurements (aerosol particle number, CCN and aerosol particle composition in the submicron fraction) from nine surface stations located in Europe and Japan. The evaluation focuses on the ability of models to simulate the average across time state in diverse environments and on the seasonal and short-term variability in the aerosol properties.

There is no single model that systematically performs best across all environments represented by the observations. Models tend to underestimate the observed aerosol particle and CCN number concentrations, with average normalized mean bias (NMB) of all models and for all stations, where data are available, of −24% and −35% for particles with dry diameters > 50 and > 120nm, as well as −36% and −34% for CCN at supersaturations of 0.2% and 1.0%, respectively. However, they seem to behave differently for particles activating at very low supersaturations (< 0.1 %) than at higher ones. A total of 15 models have been used to produce ensemble annual median distributions of relevant parameters. The model diversity (defined as the ratio of standard deviation to mean) is up to about 3 for simulated N_3_ (number concentration of particles with dry diameters larger than 3 nm) and up to about 1 for simulated CCN in the extra-polar regions. A global mean reduction of a factor of about 2 is found in the model diversity for CCN at a supersaturation of 0.2% (CCN_0.2_) compared to that for N_3_, maximizing over regions where new particle formation is important.

An additional model has been used to investigate potential causes of model diversity in CCN and bias compared to the observations by performing a perturbed parameter ensemble (PPE) accounting for uncertainties in 26 aerosol-related model input parameters. This PPE suggests that biogenic secondary organic aerosol formation and the hygroscopic properties of the organic material are likely to be the major sources of CCN uncertainty in summer, with dry deposition and cloud processing being dominant in winter.

Models capture the relative amplitude of the seasonal variability of the aerosol particle number concentration for all studied particle sizes with available observations (dry diameters larger than 50, 80 and 120 nm). The short-term persistence time (on the order of a few days) of CCN concentrations, which is a measure of aerosol dynamic behavior in the models, is underestimated on average by the models by 40% during winter and 20% in summer.

In contrast to the large spread in simulated aerosol particle and CCN number concentrations, the CDNC derived from simulated CCN spectra is less diverse and in better agreement with CDNC estimates consistently derived from the observations (average NMB −13% and −22% for updraft velocities 0.3 and 0.6 ms^−1^, respectively). In addition, simulated CDNC is in slightly better agreement with observationally derived values at lower than at higher updraft velocities (index of agreement 0.64 vs. 0.65). The reduced spread of CDNC compared to that of CCN is attributed to the sublinear response of CDNC to aerosol particle number variations and the negative correlation between the sensitivities of CDNC to aerosol particle number concentration (*∂N*_d_/*∂N*_a_) and to updraft velocity (*∂N*_d_/*∂w*). Overall, we find that while CCN is controlled by both aerosol particle number and composition, CDNC is sensitive to CCN at low and moderate CCN concentrations and to the updraft velocity when CCN levels are high. Discrepancies are found in sensitivities *∂N*_d_/*∂N*_a_ and *∂N*_d_/∂*w*; models may be predisposed to be too “aerosol sensitive” or “aerosol insensitive” in aerosol–cloud–climate interaction studies, even if they may capture average droplet numbers well. This is a subtle but profound finding that only the sensitivities can clearly reveal and may explain inter-model biases on the aerosol indirect effect.

## 1 Introduction

Aerosol particles absorb and scatter radiation, thereby modulating the planetary radiative balance (Boucher et al., 2013; Myhre et al., 2013). They also provide the nuclei upon which cloud droplets and ice crystals form; variations thereof can profoundly impact cloud formation and precipitation. Both the direct radiative effects of aerosols and their impacts on clouds are thought to be important for climate at global and regional scales, although they are highly uncertain and confound projections of anthropogenic climate change (e.g., Boucher et al., 2013; Seinfeld et al., 2016). The impacts of aerosols on clouds in particular introduce considerable uncertainty in our estimates of equilibrium climate sensitivity and transient climate response to the combined changes in aerosol and greenhouse gas concentrations (e.g., Seinfeld et al., 2016; Fan et al., 2016).

Aerosols can be either directly emitted from a variety of sources (primary aerosols) or formed by nucleation from precursor compounds (secondary aerosols), which afterwards can grow by condensation and coagulation from a few nanometers to a few hundred nanometers (Kerminen et al., 2012). Note that secondary aerosol also includes the condensed material upon primary emitted aerosol. Aerosols that have the potential to create cloud droplets at atmospherically relevant conditions are termed cloud condensation nuclei (CCN). The CCN number concentration depends on the particle size distribution, chemical composition and mixing state, as well as the level of water vapor supersaturation that develops in rising air parcels (Köhler, 1936; Seinfeld and Pandis, 2006). It is now established that primary emissions of particulate matter and particle formation from anthropogenic precursor gases have strongly modulated clouds and climate at the global scale since the industrial revolution (Boucher et al., 2013). Much work remains, however, to reduce the uncertainty associated with anthropogenic aerosol–cloud–climate interactions.

Among the main sources of uncertainty in simulating aerosol microphysics at regional to global scales are the amounts of particle and precursor vapor mass emitted by anthropogenic activities or natural sources, as well as the size distribution of the emitted particles and their representation in models. However, Mann et al. (2012) showed that a careful choice of the aerosol parameters describing the aerosol distribution can reduce differences between the sectional and the modal description of aerosol microphysics in most parts of the atmosphere. Furthermore, carbonaceous combustion aerosol, although assumed hydrophobic upon emission, was found to contribute up to 64% of global surface CCN concentrations (Spracklen et al., 2011). Although less important than particle size for CCN formation, particle chemical composition determines aerosol hygroscopicity (Twomey, 1977; Dusek et al., 2006; Petters and Kreidenweis, 2007; Cubison et al., 2008; Bougiatioti et al., 2009). An adequate description of aerosol hygroscopicity is required to accurately describe CCN and cloud droplet number variability. In this respect, uncertainties are partially related to organic aerosol (OA), which can be composed of thousands of compounds with different physical and chemical properties. OA contributes to the fine aerosol mass by up to 30%–70% depending on location and season (Kanakidou et al., 2005; Jimenez et al., 2009), while source estimates of OA span 1 order of magnitude (see the AEROCOM phase II intercomparison study of 31 models by Tsigaridis et al., 2014). Regionally, sea salt (SS) and mineral dust (DU) are also significant contributors to the total aerosol particle mass and number concentration. Atmospheric mass loads during the first phase of AEROCOM showed a high diversity among 15 models of 54% for SS and 40% for DU (Textor et al., 2006). This diversity arises from the different parameterizations used to calculate the size-resolved fluxes and their dependence on wind speed but also from the consideration, or not, of the super-coarse aerosol fraction (Huneeus et al., 2011; Tsigaridis et al., 2013). Although nitrate $\left({\text{NO}}_{3}^{-}\right)$ and ammonium $\left({\text{NH}}_{4}^{+}\right)$ are not explicitly studied here, differences of up to a factor of 13 in the atmospheric burden of ${\text{NO}}_{3}^{-}$ and 17 and 4 for NH_3_ and ${\text{NH}}_{4}^{+}$, respectively, have been found between AEROCOM models (Bian et al., 2017).

Formation of new particles by nucleation in the atmosphere is a frequent phenomenon in the free troposphere and in the continental boundary layer (e.g., Kerminen et al., 2010; Kulmala and Kerminen, 2008), and it is an important source of aerosol particle number on a global scale (Kerminen et al., 2012; Kalivitis et al., 2015; Gordon et al., 2017). Although it is well established that sulfuric acid, due to its low volatility, plays a central role in new particle formation and growth, it cannot explain the observed substantial growth of small particles in many environments where organics and NH_3_ are abundant. This is due to the low concentration of sulfuric acid and is evidenced by the observed poor correlation of its concentration with very small particles (e.g., Pierce et al., 2011). Recently, the involvement of organics from early stages of nucleation and growth of particles has been established (e.g., D’Andrea et al., 2013; Spracklen et al., 2008; Makkonen et al., 2009; Tröstl et al., 2016). Several approaches for modeling particle growth in large-scale models have been developed, which are very sensitive to the volatility of organic vapor (e.g., Laaksonen et al., 2008; Yu, 2011; D’Andrea et al., 2013) and are being implemented in global models.

The number concentration and the size of cloud droplets depend on both the concentrations of CCN and on the cloud updraft velocity (Pruppacher and Klett, 1997; Seinfeld and Pandis, 2006). However, the spatial scale of updrafts governing droplet formation is several orders of magnitude smaller than the size of the grid boxes of global models. Therefore, parameterized aerosol–cloud interactions in climate models require sub-grid-scale vertical velocity distributions to calculate grid-scale relevant cloud droplet number concentration (CDNC) (Morales and Nenes, 2010). Karydis et al. (2012) and Moore et al. (2013) have shown that in regions with low particle number concentrations, such as the Arctic and remote oceans, CDNC is more sensitive to CCN uncertainty than in continental regions where particle number concentrations exceed 10^4^ cm^−3^. In contrast, Ervens et al. (2010) pointed out that at high updraft velocities, supersaturation is controlled by adiabatic cooling, and CDNC is not very sensitive to errors in simulated CCN number concentration. They estimated that uncertainties in the chemical composition of aerosol particles that could lead to a doubling of CCN concentration would affect CDNC by only about 10%–20%. Therefore, there are two distinct regimes with regard to CDNC sensitivity: aerosol limited and updraft velocity limited (Reutter et al., 2009).

Totally different cloud radiative (indirect) effects could be computed by climate models depending on the dominance of CDNC sensitivity to either aerosol number or updraft velocity (Sullivan et al., 2016). Therefore, capturing the balance between the two is critical in understanding where and when aerosol emissions are governing the variability of cloud properties and where the updraft velocity is the controlling factor. The failure of state-of-the-art models to capture such sensitivity implies that even if models exhibit a similar magnitude of aerosol indirect effects, it may be for completely different reasons (Sullivan et al., 2016). In this case models would show limited skills and their predictions would be associated with low confidence.

The aims of this work are to (i) assess the accuracy of state-of-the-art global aerosol models in simulating the chemical composition and number concentration of aerosol particles, with a focus on CCN concentrations at various water vapor supersaturation ratios, (ii) document the diversity of the global models in simulating these aerosol properties, (iii) produce an ensemble view of the global distribution of aerosol particle and CCN number concentrations, together with the most important particle chemical components at the Earth’s surface, (iv) evaluate the agreement of inferred CDNC from modeled and from observed CCN spectra and their sensitivity to aerosol number concentrations and updraft velocities, (v) evaluate the potential causes of model diversity and bias versus observations using model uncertainty analysis, and (vi) provide recommendations for future model improvements.

A total of 16 global models contributed to this study, and multiyear observations of CCN, size-resolved particle number concentration distributions, and particle chemical composition obtained from eight atmospheric monitoring stations in Europe and one in Japan were used as an observational reference, representing distinct atmospheric environments (Schmale et al., 2017, 2018).

## 2 Methodology

### 2.1 Contributing models and model description

Model setup, such as spatial resolution, meteorological conditions and emission inventories, differs significantly among models (Tables S1 to S4 in the Supplement). The spatial resolution varies among the models from 0.94° by 1.3° to 4° by 5.0° (latitude by longitude) and from 25 to 56 vertical layers up to 10 and even 0.1 hPa. Nine of the models are general circulation models (GCMs) and six are chemical transport models (CTMs). The CTMs use prescribed (and different) meteorological datasets, while the GCMs (with the exception of GISS-E2-TOMAS) are nudged to various reanalysis products. Atmospheric transport, secondary aerosol formation and removal of aerosols are driven by wind, temperature, radiation, precipitation and relative humidity, as well as cloud fraction and liquid water content. In addition, most of the models use wind-driven dust, sea salt and marine organic aerosol emissions as well as calculated online biogenic emissions of non-methane volatile organic compounds (NMVOCs) (Table S3). Therefore, meteorology significantly affects number concentration, composition and other metrics of aerosol particles.

Despite the recognized importance of organic compounds in nucleation (Tröstl et al., 2016), several global models that participated in the present study use the binary homogeneous nucleation of sulfuric acid and water (referred to later as BHN; e.g., Kulmala et al., 1998; Vehkamäki, 2002) and the contribution of organics to particle growth (see Sect. S1 and Table S2 and references therein). GEOS-Chem-TOMAS assumes a ternary nucleation mechanism when NH_3_ is present and a binary one when NH_3_ is absent. GEOS-Chem-APM and CAM5-Chem-APM employ a ternary ion-mediated nucleation (TIMN) scheme that considers both binary and ternary as well as ion-mediated and neutral nucleation (Yu et al., 2018). New particle formation in TM5 is calculated as a combination of BHN and organic–sulfuric acid nucleation (Riccobono et al., 2014).

Once in the atmosphere, aerosols undergo transformations through chemical and physical processes, such as coagulation, condensation and evaporation, that modify their size and physical and chemical properties. These aerosol microphysical processes are parameterized differently in models. Eight of the models use modal schemes in which the evolution of particle number and mass concentrations is described by lognormal distributions, and the remaining models use the sectional approach with various numbers of monodisperse size bins describing aerosol particle number concentration and chemical composition (Table S2).

Regarding the eight modal models, six of them (the three ECHAM models, EMAC, TM4-ECPL and TM5) are based on the M7 aerosol module developed by Vignati et al. (2004) for the description of aerosol microphysics or improved versions of M7 to account for SO_2_ oxidation to sulfuric acid, the contribution of organics to growth and additional aerosol species. Other aerosol microphysics modules used in models participating in this study are the Modal Aerosol Modules (MAM3 and MAM4; Liu et al., 2012, 2016), the Advanced Particle Microphysics (APM) package (Yu and Luo, 2009; Yu, 2011; Yu et al., 2018), the TwO-Moment Aerosol Sectional (TOMAS) microphysics package (Adams and Seinfeld, 2002), the Multiconfiguration Aerosol Tracker of mIXing state (MATRIX) module (e.g., Bauer et al., 2008), the Aerosol Two-dimensional bin module for formation and Aging Simulation version 2 (ATRAS2; Matsui, 2017) and a production-tagged module OsloAero5.3 used in combination with the offline microphysics scheme AeroTab5.3 (Kirkevåg et al., 2018). Tables S1, S2, S3 and S4 provide a summary of the main features of the participating models and appropriate references.

Relevant to this study are also differences in the aerosol components that are taken into consideration in the models for the CCN calculations. Nine models (CAM5-MAM3, CAM5-MAM4, CAM5.3-Oslo, the three ECHAM models, GEOS-Chem-TOMAS, GISS-E2-TOMAS models and TM4-ECPL) do not account for particulate nitrate at all or in the CCN calculations (Table S2). TM4-ECPL, however, computes the ${\text{NO}}_{3}^{-}$ and ${\text{NH}}_{4}^{+}$ mass distribution in fine and coarse modes with the ISORROPIA II module (Fountoukis and Nenes, 2007). Similarly, TM5 uses EQSAM (Metzger et al., 2002b, a) to calculate, using a bulk aerosol approach, the partitioning of ammonium nitrate between the gaseous and particulate phase with the particulate mass assumed to reside in the soluble accumulation mode.

Both dry deposition and wet deposition of aerosol particles are taken into account in the participating models as shown in Table S4. For dry deposition, models account for gravitational settling and for turbulence, and thus these processes depend on the aerosol particle size. The omission of super-coarse particle sources associated with dust and sea-salt particles results in discrepancies between models and between model results and observations (Myriokefalitakis et al., 2016). Wet deposition parameterizations account for both in-cloud scavenging, which is sensitive to the solubility of aerosol particles, and below-cloud scavenging by convective and large-scale precipitation (Seinfeld and Pandis, 2006). In addition, while all models account for in-cloud scavenging of aerosols and for aerosol release from the evaporation of droplets, a few models also account for melting and sublimation of ice crystals. For the calculation of CCN concentrations from the aerosol number and mass distributions, models need to specify their hygroscopicity from the volume-weighted hygroscopicities of each constituent (Table 1) following the approach of Petters and Kreidenweis (2007).

Furthermore, most of the participating models (Table S4) follow the AEROCOM recommendation of biomass burning emission heights, which in the boreal regions extend above 2 km and up to 6 km for the Canadian boreal fires (Dentener et al., 2006). ECHAM6-HAM2 and ECHAM6-HAM2-AP use a slightly different vertical distribution of biomass burning emissions, with 75% within the planetary boundary layer (PBL), 17% in the first and 8% in the second level above the PBL (Tegen et al., 2019). EMAC assumes biomass burning emissions at 140 m and GEOS-Chem-APM well mixed in the boundary layer.

In addition to these 15 models, we include the results from perturbed parameter ensemble (PPE) simulations using HadGEM3-UKCA (Yoshioka et al., 2019; see details in Sect. S1). The PPE consists of 235 atmosphere-only simulations for the year 2008 with 26 parameters controlling aerosol emissions and processes perturbed simultaneously. Simulations were nudged to ERA-Interim wind and temperature and all aerosol feedbacks to atmospheric dynamics are turned off. Therefore, all simulations share the same meteorology. CCN number concentrations were calculated globally for all member simulations and taken at geographical locations and elevations of observation stations. These simulations were then used to create Gaussian process emulators at each station location from which 260 000 “model variants” were generated that densely sample the 26-dimension parameter space. The emulator was validated against additional model simulations to show that the emulator uncertainty is much smaller than the model parametric uncertainty.

### 2.2 Observational data for model evaluation

**Figure 1.**
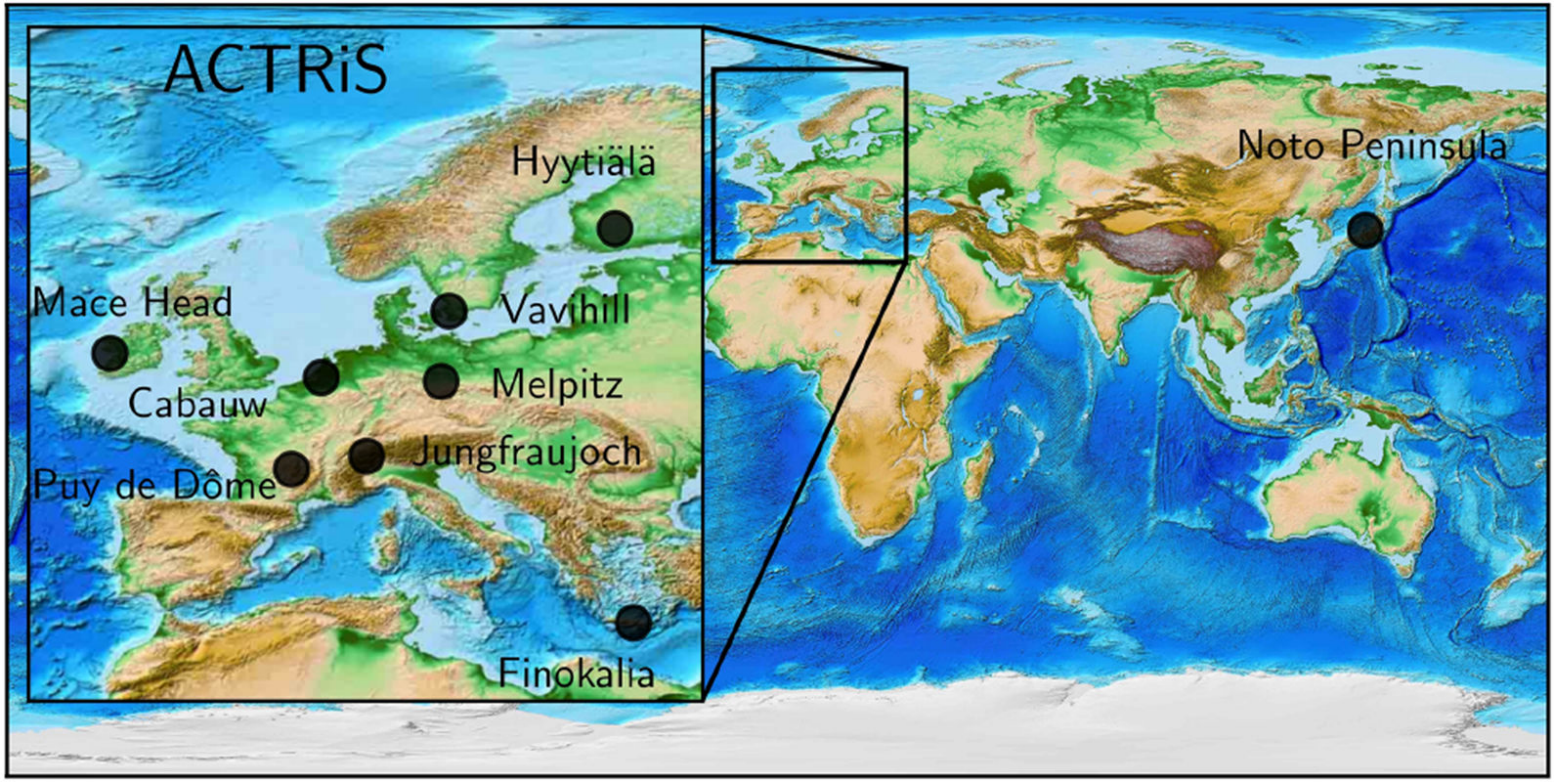
Map showing the location of the measurement sites used in this study.

Datasets for CCN at various supersaturations, particle number concentrations, size distributions and particle chemical compositions measured at one atmospheric monitoring station in Japan and eight Aerosols, Clouds, and Trace gases Research InfraStructure (ACTRIS) atmospheric monitoring stations in Europe (Schmale et al., 2017) were used in the present study (Fig. 1) for the evaluation of model results. The observatories are representative of different environments (Pacific, Atlantic and Mediterranean marine atmospheres, high alpine and boreal forest continental atmospheres). A brief site description of the observatories is provided in Table S5, while more technical details are given by Schmale et al. (2017). While in general measurement data are available from the period 2011 to 2015, each station covered only a subperiod of those 5 years but at least one entire year (Schmale et al., 2017). Despite using point measurements, the long period of observations allows for the evaluation of global models without biases associated with the model resolution (Schutgens et al., 2016). Six out of the nine stations provided non-refractory chemical composition data on submicron particles (based on aerosol mass spectrometry), while all stations recorded submicron particle number size distributions and CCN number concentrations over a variety of supersaturations. A detailed discussion of the observational results can be found in Schmale et al. (2018).

For this study, the observations of CCN concentrations at supersaturations spanning between 0.1% and 1.0%, the number concentrations of aerosols with dry diameters larger than 50, 80 and 120 nm (denoted hereafter as N_50_, N_80_ and N_120_, respectively), and PM_1_ (particles with dry diameters less than 1 μm) chemical composition (mainly sulfate (${\text{SO}}_{4}^{2-}$, hereafter SO4) and organic aerosol – OA) from the nine stations are used. The CCN data for these stations cover at least 75% of each year (Schmale et al., 2017). Observational data have been further filtered so that there is a minimum data requirement, which means that daily averages are calculated from hourly data only for days with at least six hourly measurements. Monthly averages follow a similar method, whereby the average is calculated only for months with at least 10 daily averages. When fewer data are available, the data are not considered representative of this quantity and are not included in the comparisons with the model results.

### 2.3 Design of the experiment

This model experiment has been designed within the BACCHUS EU project and has been opened for participation to the entire AEROCOM global modeling community. Global simulations have been performed for the years 2010–2015 (2010 is used as a spin-up). SO4, BC, OA, SS and DU are the aerosol components that are considered here. Models provided hourly values for N_50_, N_80_, N_120_ and CCN number concentrations for 13 supersaturations ranging from 0.05% up to 1.0% (these are 0.05%, 0.075%, 0.1%, 0.15% and from 0.2% to 1.0% in increments of 0.1%, denoted hereafter as CCN_*i*_, where *i* is the supersaturation value), as well as the chemical composition of PM_1_ particles at the station locations (Table S5). The large number of different supersaturations at which CCN are computed allows for direct comparisons with all available observations of CCN for the nine stations as well as for the calculation of CDNC (Sect. 2.4). Among the models that participated in the present study GISS-E2-TOMAS and HadGEM3-UKCA did not provide any results for the stations due to meteorology not corresponding to the measurement time period (free running for the first one and 2008 for the second); therefore, all multi-model medians (MMMs) for the stations presented below have been computed excluding these models.

Beyond station data, the global annual mean surface distribution of CCN_0.2_, the particle numbers N_3_, N_50_ and N_120_, and the mass composition of the PM_1_ particles for the year 2011 are provided by 15 models (HadGEM3-UKCA did not provide such results). The MMM has been computed as the median of the contributing models.

In addition to the data provided by the 15 global models, the results of the PPE using HadGEM3-UKCA (Yoshioka et al., 2019) are used in this study to quantify the model parametric uncertainty in CCN and to perform a sensitivity analysis to quantify how each parameter contributes to the overall uncertainty.

### 2.4 Data interpretation methodology

#### 2.4.1 CCN persistence

To investigate the duration for which the CCN number concentration remains similar to its earlier concentration, the so-called persistence, the autocorrelation function (ACF) of the CCN time series, has been calculated as in Schmale et al. (2018) (see also Sect. S2). This ACF may provide valuable information about the drivers of the variability of the CCN number concentration in the atmosphere. In the present study, we chose to compute the ACF based on model results of CCN_0.2_ at the nine sampling sites and compare them with the corresponding ACF obtained from observations (Schmale et al., 2018). For a direct comparison, we use the same time periods as for the observations, which vary among the sampling sites. For all ACF calculations, hourly data on CCN_0.2_ were used for both the observations and model results.

#### 2.4.2 CDNC calculations

While GCMs calculate CDNC using a variety of approaches, for the present study CDNC is calculated offline using a common parameterization for CCN spectra derived from the models or from the observations. This approach allows for an understanding of the importance of differences in modeled and observed CCN spectra by expressing them as differences in CDNC that would form in a given type of cloud. We have calculated CDNC for two different updraft velocities: one characteristic for stratiform clouds (*w* = 0.3 ms^−1^) and the second characteristic for cumulus clouds (*w* = 0.6 ms^−1^), where *w* is the width of the vertical velocity distribution assuming a zero mean Gaussian. Similar calculations have been performed using the observed CCN spectra at the stations where such information is available to enable comparison of model results with observations. The ability of the modeled CCN spectra to reproduce the observed sensitivity of CDNC to aerosol or to updraft velocity is also evaluated. Note that evaluation of the differences in CDNC calculations by the different models that are derived from both the parameterizations used and from their input variables would require a different model intercomparison design than here and is planned for the future. Morales Betancourt and Nenes (2014a) provide a good example in which the source of CDNC prediction discrepancy for two state-of-the-art parameterizations in the CAM5 global model was unraveled using adjoint sensitivity analysis. That study pointed to exactly which aspects of the parameterization (i.e., water uptake from large CCN) were not captured adequately, leading to the highly improved droplet parameterizations (Morales-Betancourt and Nenes, 2014b) that were used in the current study.

The calculation of CDNC is based on the parameterization of Nenes and Seinfeld (2003) with the mass transfer augmentations proposed by Fountoukis and Nenes (2005), Barahona and Nenes (2007), and Morales Betancourt and Nenes (2014b). Using the CCN at different supersaturations (Sect. 2.3) allows us to consistently construct the CCN spectrum function *F*(*s*) from each simulation, which provides the CCN number as a function of supersaturation, *s* (Sotiropoulou et al., 2006):

(1) $$F(s)=\frac{N}{1+(\frac{s}{b}{)}^{a}},$$

where *N* is the total number of particles, and *a* and *b* are parameters determined using a nonlinear fitting procedure for each of the participating models. *F*(*s*) is then computed for each station’s grid point and time step of the model outputs (with *b* and *a* being fitting parameters), and CDNC, denoted in the figures by *N*_d_, is computed from the parameterization for prescribed values of the vertical velocity. This fitting approach has also been applied to the CCN observations since they are available only for a limited number of supersaturations and thus cannot be directly used for accurate calculation of CDNC. A well-constrained CCN spectrum requires concentrations for at least five different supersaturations at the same time instance (Sotiropoulou et al., 2006). Such information was available only at five stations (Cabauw, Finokalia, Jungfraujoch, Mace Head and Vavihill), which is subsequently used for deriving CDNC based on observations and compared against model-derived CDNC.

The CDNC parameterization uses as input *F*(*s*), cloud-base pressure and temperature, and the vertical velocity characterizing the cloud updraft (either as a single updraft or a “characteristic” value that provides a distribution-averaged value; Morales and Nenes, 2010). It determines the value of maximum supersaturation, *s*_max_, that develops in the cloudy updrafts using the concept of “population splitting” (Nenes and Seinfeld, 2003). *s*_max_ is achieved during the cloud parcel ascent and is calculated considering the water vapor balance between its availability from cooling and its loss from condensational growth of the CCN (Fountoukis and Nenes, 2005). CDNC is then obtained from the CCN spectrum as *N*_d_ = *F* (*s*_max_). This approach works well for stratus and stratocumulus clouds (Morales and Nenes, 2010). CDNC calculated here is from primary activation and does not consider the influence of preexisting droplets, although modifications to the parameterization can account for this as well (e.g., Barahona et al., 2014).

#### 2.4.3 Ensemble modeling computation

The modeled hourly aerosol particle number concentrations, mass composition, CCN and CDNC at the nine stations have been used to calculate daily and monthly averages. Comparison of individual model results with observations is provided in Figs. S2 and S3 because it can be used to identify the strengths and weaknesses of each specific model and can serve as a guide for model improvements in the future. In Sect. 3, the multi-model median (MMM) is compared to observations. The diversity of the model results (defined as the ratio of standard deviation to mean) and the mean of the models, which in several cases significantly differs from the MMM, are also reported in these comparisons.

Annual averages of the global surface distributions of N_3_, N_50_, N_120_, CCN_0.2_ and PM_1_ mass concentrations of the major aerosol components have been provided by a total of 15 models. Global fields have first been re-gridded to a 5° × 5° grid for all models, which is close to the coarsest-resolved participating models (4° × 5°). Then the MMM and diversity are calculated, as described above, for the stations. Note that 5° × 5° is a very coarse grid size, which no doubt affects the model-to-observation comparison, particularly when comparing to sites within small heavily polluted areas where a large rural background is now also being added in and vice versa. Therefore, it is worth mentioning that the surface stations used for model comparison are representative of the larger area in which they are located and justify our choice for a relatively large grid to re-grid all model results. For the mountain stations, the appropriate model level has been considered that corresponds to the station’s altitude above sea level. Annual means of the individual models are also presented in Figs. S6-S14.

#### 2.4.4 Performance indexes

For the comparison of model results with observations, a number of statistics variables have been calculated and defined as shown in Sect. S3.2. Hereafter we discuss the following:

the index of agreement

$$\left(\text{IoA}=1-\frac{\sum_{i=1}^{N}(P_{i}-O_{i}{)}^{2}}{\sum_{i=1}^{N}(|O_{i}-\bar{O}|+|P_{i}-\bar{O}|{)}^{2}}\right),$$

the normalized mean bias

$$\left(\text{NMB}=\frac{\sum_{i=1}^{N}(P_{i}-O_{i})}{\sum_{i=1}^{N}O_{i}}\times 100\%\right)$$

and the normalized mean error

$$\left(\text{NME}=\frac{\sum_{i=1}^{N}|P_{i}-O_{i}|}{\sum_{i=1}^{N}O_{i}}\times 100\%\right),$$

where *M* represents model results, *O* represents observations, $\bar{O}$ stands for the mean of the observations, and normalized mean bias (NMB), normalized mean error (NME) and the index of agreement (IoA) are used to quantify the performance of the models to reproduce observations. The IoA is a measure of the agreement of model results with the observations. In this study we use all three for the evaluation of the capability of the models to reproduce the observations.

We calculate also

the Pearson linear regression coefficient,

$$\left(r=\left[\frac{\sum_{i=1}^{N}(P_{i}-\bar{P})\;(O_{i}-\bar{O})}{\sqrt{\sum_{i=1}^{N}(P_{i}-\bar{P}{)}^{2}\sqrt{\sum_{i=1}^{N}(O_{i}-\bar{O}{)}^{2}}}}\right]\right),$$

as a measure of the ability of the model results to represent the variability in the observations.

## 3 Evaluation against station observations

### 3.1 CCN number concentration comparisons with multi-model median

**Figure 2.**
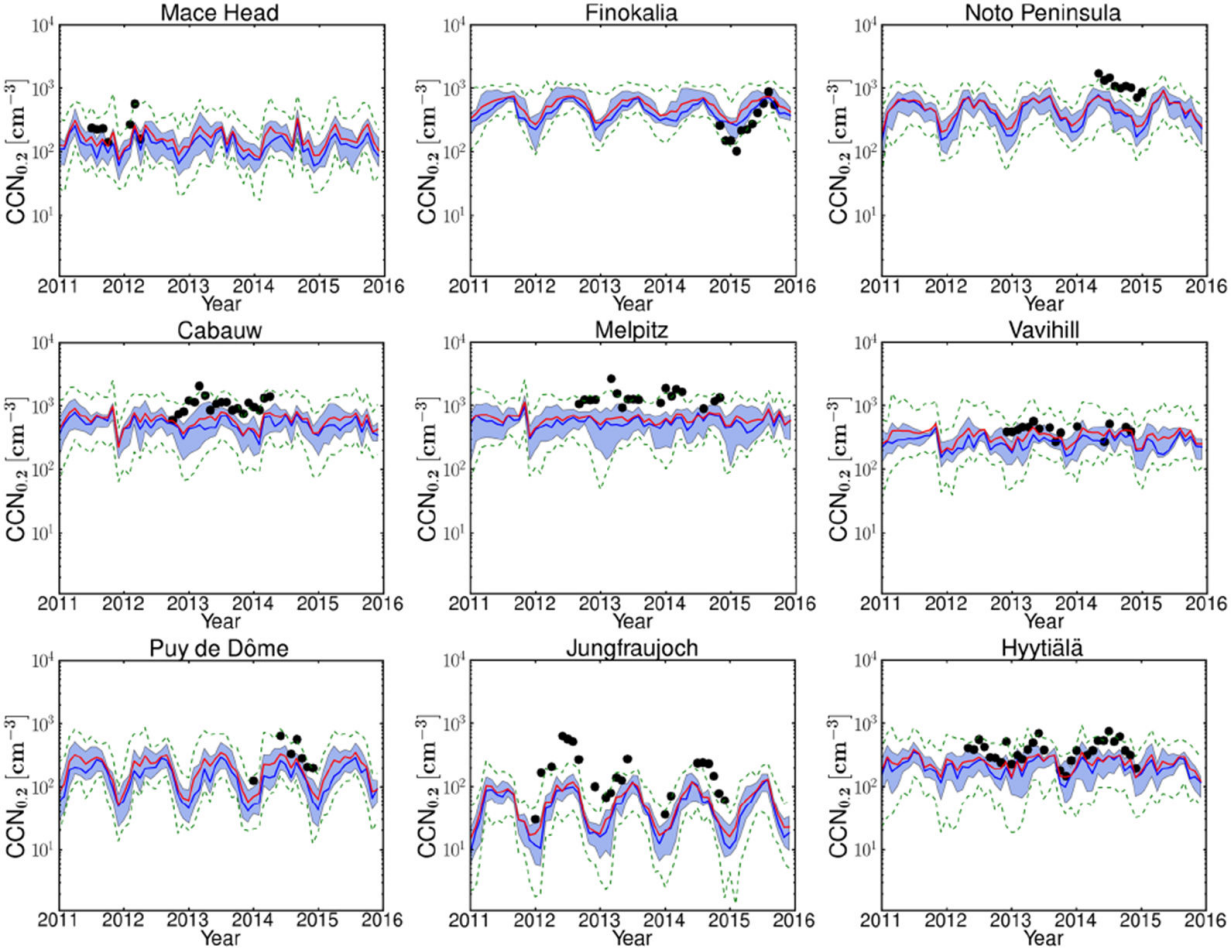
Monthly ensembles for the years 2011–2015 of the CCN number concentration for supersaturation 0.2% (CCN_0.2_). The CCN_0.2_ obtained from observational data is shown with symbols. The continuous bold blue and red lines show the monthly median and mean of all models, respectively. The shaded area shows 25% and 75% of the model results, while the green dashed lines show the minimum and maximum values of all models.

The models tend to underestimate the monthly CCN_0.2_ number concentration in the lowest model level at all sites (Figs. 2 and S2) for the years 2011–2015: the average NMB of all models and for the nine sites is as low as −36%, and the NME is 69%, while among individual models and stations NMB and NME vary from −88% to 145% and from 40% to 159%, respectively (see Sect. S3.2 for definitions and Table S6 for results). The Finokalia station is an exception, where most models overestimate CCN_0.2_ (average NMB around 47%) with eight models showing significant overestimation (NMB > 10%) and six models smaller deviations from observations (−10% < NMB < 10%). Among the studied locations, Finokalia is the station with the highest observed critical diameter (~ 200 nm at a supersaturation of 0.2% according to Schmale et al., 2018); therefore, potential inaccuracies in the model determination of the critical size may be responsible for the model overestimate of CCN_0.2_ at this station.

Such a hypothesis is supported by earlier studies that have observed a large size dependence of sensitivity in the activation fraction at low supersaturations and in the size ranges between 60 and 100 nm (Bougiatioti et al., 2011). Deng et al. (2013) reported inferred critical diameters varying by factors of 2–3 for low supersaturations from 0.06% to 0.2% and suggested the use of size-resolved particle number concentrations with inferred critical diameters or size-resolved activation ratios to predict CCN. Errors in CCN predictions have been shown to exceed 50% only at very low supersaturations (Reutter et al., 2009), reaching a factor of 2.4, while at high supersaturations CCN overestimates can be less than 5% (Ervens et al., 2007). The global near-surface mean CCN prediction error has been estimated at about 9%, and regionally the maximum error can reach 40% (Sotiropoulou et al., 2007). The largest CCN prediction error was found in regions with low in-cloud *s*_max_, like those affected by long-range transport of pollution or industrial pollution plumes. Lower CCN prediction error was found in regions where in-cloud *s*_max_ is high, which is typical for pristine areas. Sotiropoulou et al. (2007) also found that the assumption of a size-invariant chemical composition of internally mixed aerosol increases the error by a factor of 2.

The underestimation of the observed CCN_0.2_ by the models is largest at the high alpine site of Jungfraujoch (mean NMB of all models: −73%), where none of the models are able to capture the maximum observed values of CCN_0.2_ (~ 300–600 cm^−3^) during summer. Deficiencies in the models’ representation of the boundary layer and mixing of air between the boundary layer and the free troposphere in complex terrain like the Alps, as well as the sampling of the models based on the station’s altitude, might be reasons for this systematic underestimation by the models (D’Andrea et al., 2016). Despite the quantitative differences in the estimation of the CCN_0.2_ concentrations, models are able to qualitatively capture the relative differences in CCN_0.2_ concentrations between stations and their seasonal variations. Comparing the CCN_0.2_ as calculated from the observations and as computed from the daily MMM for the days with available observations for the stations, we find a Pearson linear correlation coefficient (*r*) that varies between 0.44 (for Melpitz) and 0.83 (for Mace Head), showing significant covariation of model results with observations. Furthermore, ranking the stations based on the observed mean CCN_0.2_ levels (Fig. S17) we find that the corresponding MMM mean follows this station ranking with the exception of Finokalia where, as further discussed, the models overestimate the observed CCN_0.2_, although they capture (*r* = 0.76) the observed temporal variability well. The MMM index of agreement (IoA) varies between 0.44 and 0.82 for the different stations, with the best for the Finokalia remote coastal station and the worst for the Jungfraujoch alpine station. The largest difference in performance among models is found for the Mace Head station with an IoA varying between 0.20 and 0.89 for the individual models (Table S6).

**Figure 3.**
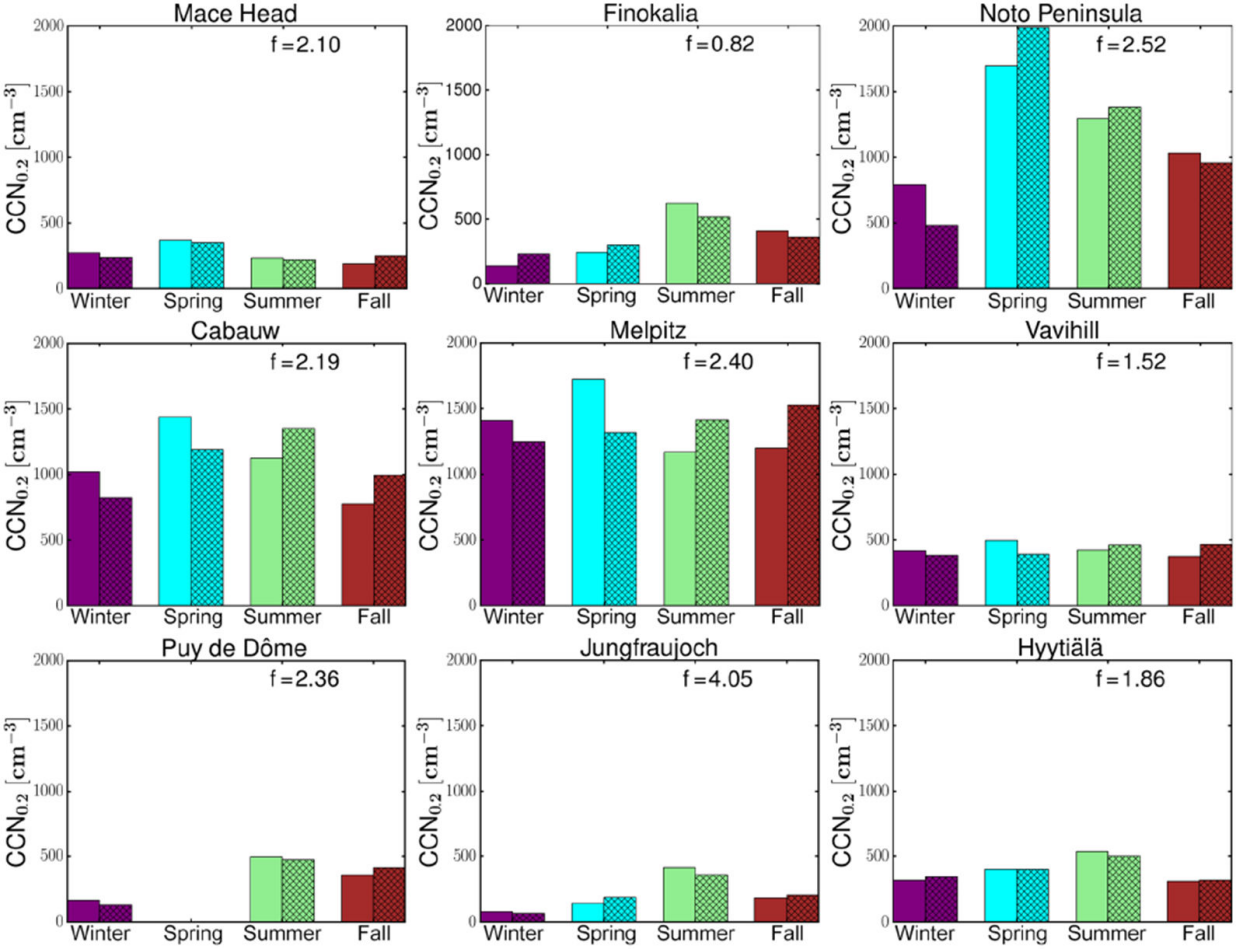
Comparison of the seasonal variations of the observed and model median computed CCN_0.2_. The solid bars show the average of the observed CCN_0.2_ during each season and the shaded bars the corresponding averages of the model results. The simulated CCN_0.2_ concentrations have been scaled by a factor, *f* (denoted in each graph), so that the four-season mean is the same as the observed one. For Puy de Dôme the normalization is based on the mean of three seasons (winter, summer and fall) due to data availability.

To compare the calculated MMM and the observed seasonal variability of CCN_0.2_ for each station (Fig. 3), the monthly model results have been temporally co-located with monthly mean observations. Furthermore, to increase clarity in Fig. 3, for each station, the MMM CCN_0.2_ has been multiplied by a scaling factor, *f*, so that the four-season mean of the simulated MMM CCN_0.2_ concentrations becomes equal to the corresponding observed value. The factor *f* is denoted for each station inside the frame. Overall, the seasonal pattern is nicely captured by the models, although the absolute values are underestimated everywhere (*f* > 1.50) except at Finokalia (*f* = 0.82) as discussed earlier.

For the high-altitude continental background sites (Puy de Dôme, Jungfraujoch) low number concentrations with high seasonal variability are observed (winter (DJF) minimum and summer (JJA) maximum with observed summer-to-winter ratios of 2.17 and 5.37, respectively, while the simulated MMM ratios are 3.19 and 5.58). This strong seasonality is attributed to changes in the height of the boundary layer that can affect these sites during summer but not during winter when the sites are mostly in the free troposphere (Schmale et al., 2018; Jurányi et al., 2011). At Jungfraujoch the boundary layer virtually never reaches up to the site. Instead, increased concentrations are caused by injections of boundary layer air into the lower free troposphere over the mountainous terrain. The free tropospheric background concentration of CCN is very low such that increases in the number concentration of CCN-sized particles (90 nm in diameter) are a good indicator for boundary layer influence (Herrmann et al., 2015).

On the other hand, high CCN_0.2_ number concentrations but low seasonal variability are found for the rural background stations of Cabauw and Melpitz, indicative of the elevated air pollution background in these regions. At these stations the highest CCN_0.2_ number concentrations are observed during spring, which are underestimated by the MMM. Furthermore, observations show a monotonous decrease from spring to summer and fall, while models calculated higher summertime values than in spring and fall at Cabauw and a monotonous increase from spring to fall at Melpitz. This could indicate that the models are not following the observed changes in the aerosol particle number concentration and/or the critical diameter at these stations (Schmale et al., 2018), possibly also associated with the adopted sizes in the primary aerosol emissions at these locations. At the other rural background station (Vavihill), both models and observations show lower CCN_0.2_ concentrations and seasonal variability than at Cabauw or Melpitz. In addition, observations indicate a higher critical diameter at Vavihill (around 120 nm) than at the other two stations (around 90 nm) (Schmale et al., 2018).

Different seasonal cycles are also observed among the three coastal sites Mace Head, Finokalia and the Noto Peninsula: at the Mace Head site, due to the clean marine conditions over the Atlantic Ocean (Ovadnevaite et al., 2014), low CCN_0.2_ concentrations are observed through the year. There, the highest concentrations are observed and simulated during spring. Both Finokalia and the Noto Peninsula are impacted by long-range transport that occurs through the free troposphere and affects the surface by mixing down into the boundary layer, and the models qualitatively reproduce the observed seasonal cycles, simulating a high variation in the number concentration over the year. At Finokalia the observed and simulated summer seasonal maximum is also attributed to biomass burning plumes from northeastern Europe (Bougiatioti et al., 2016), while high CCN_0.2_ concentrations peaking in spring (observations available only for May) over the Noto Peninsula are due to pollutants originating from East Asia (Iwamoto et al., 2016; Schmale et al., 2018). However, the observed sharp decline of CCN_0.2_ during the spring (May) to summer transition over the Noto Peninsula is also reproduced by the models. At Finokalia the models qualitatively follow the observed seasonality, although the observed summer-to-winter ratio (4.6) is underestimated by the models (2.3; Fig. 3). This can be due to the CCN sensitivity to loss by deposition during winter and to OA formation and hygroscopicity during summer that combined weaken the simulated seasonality (further discussion in Sect. 5).

Finally, at Hyytiälä, on average the models calculate relatively small CCN_0.2_ number concentrations and a low seasonal variability with a maximum in concentrations in summer, in agreement with observations, although they slightly underestimate the observed summer-to-winter ratio (1.5 modeled versus 1.7 observed). As discussed further in Sect. 5, at Hyytiälä the modeled CCN_0.2_ is very sensitive to errors in OA hygroscopicity and in secondary organic aerosol (SOA) formation from biogenic organic precursors during summer. Therefore, uncertainties in OA in the models and in particular underestimates of OA are expected to affect the summer-to-winter ratio.

**Figure 4.**
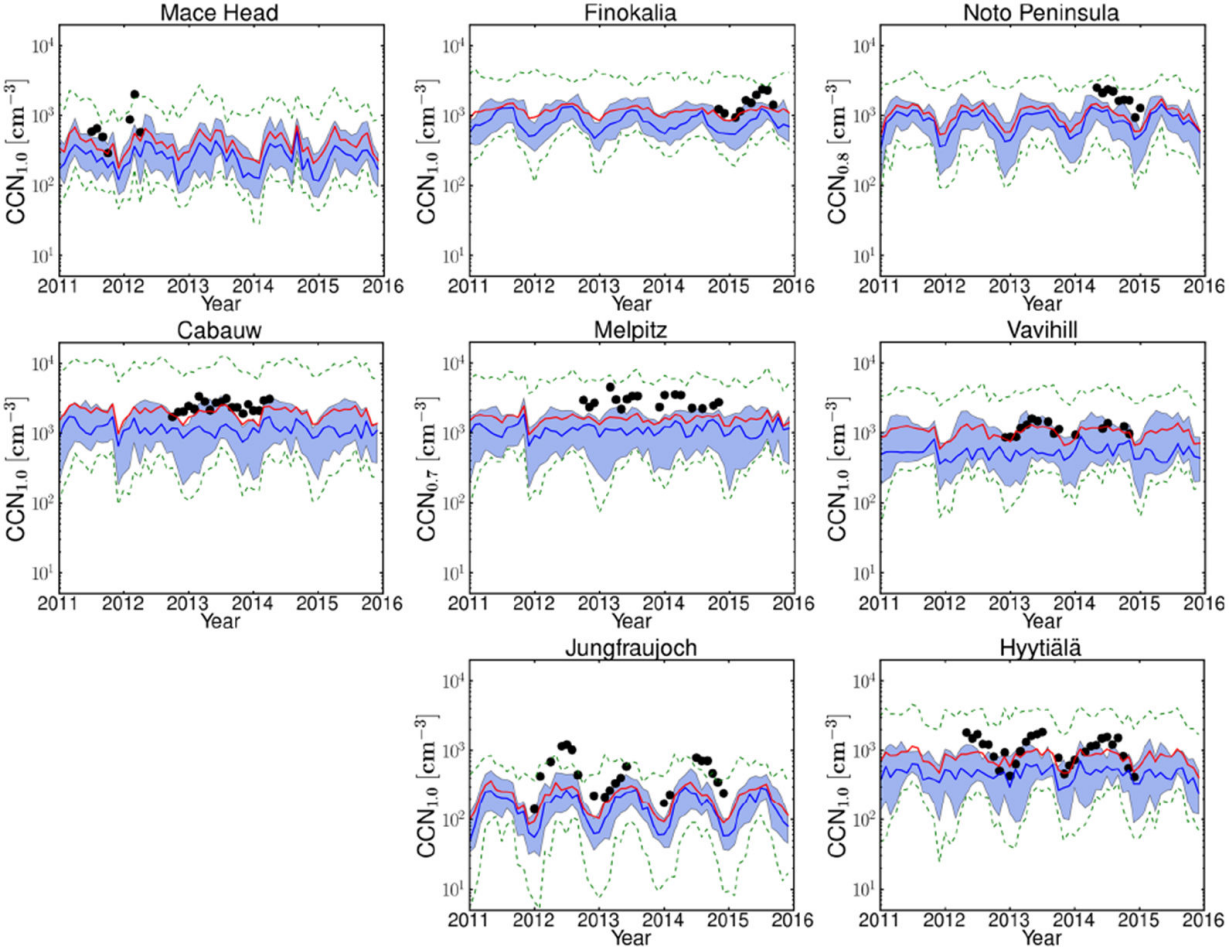
Same as Fig. 2 for the CCN at the maximum supersaturation with available measurements at each station. For Puy de Dôme only CCN_0.2_ data are available and are shown in Fig. 2.

Observed CCN number concentrations at the maximum supersaturation ratios measured at each station (which vary among stations, ranging from 0.7% to 1.0%) are compared to model results in Fig. 4. CCN at various supersaturation ratios provides insights into the size distribution and the chemical composition in the models, since at high supersaturations smaller and less hygroscopic particles also activate. Most models underestimate CCN at high supersaturation at all stations with available observations (Fig. 4), indicating that an insufficient number of small particles are predicted to activate in the model. However, observations are captured by the maximum and minimum of the 14 models (dashed green line) except for the alpine Jungfraujoch station. Overall, the average NMB and NME of all models and for all stations with available observations are −34% and 78%, respectively, while among individual models and stations NMB varies from about −89% to about 253% (Table S6).

Comparing model performance for CCN at low supersaturation (CCN_0.2_; Fig. 2) and at high supersaturation (CCN_1.0_; Fig. 4), CCN_1.0_ is systematically underestimated by the models across all stations. The NME of MMM for CCN_0.2_ ranges from 45% (Finokalia) to 81% (Jungfraujoch) for the different stations with significant correlation coefficients between 0.44 (Melpitz) and 0.86 (Mace Head), indicating that the MMM model is able to simulate the temporal variability in the observations. For CCN at the highest supersaturation with available observations the NME varies from 50% (Finokalia) to 74% (Mace Head) and the correlation coefficients from 0.37 (Melpitz) to 0.78 (Mace Head) (see also Table S6). These results indicate that CCN_0.2_ is in general better captured than CCN at higher supersaturations, both in absolute values and in temporal variability. Since the number concentration of CCN depends on both the chemical composition and the number of aerosol particles, it is worth investigating the role of these two factors separately.

### 3.2 CCN number concentration comparisons with PPE

**Figure 5.**
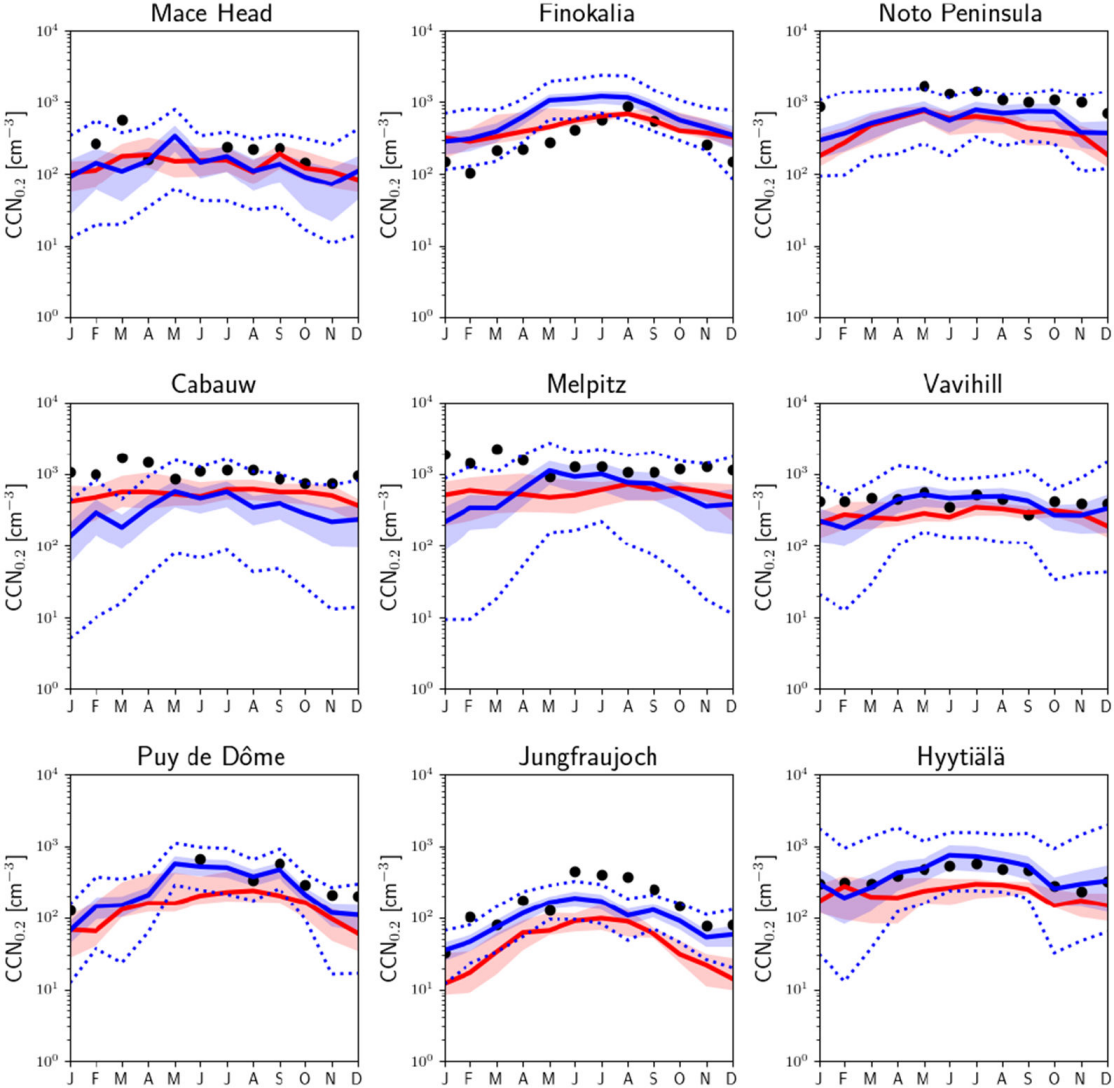
Monthly average CCN_0.2_ based on HadGEM3-UKCA perturbed parameter ensemble simulations for the year 2008. The solid blue line shows the mean of the sample of 260 000 model variants from the emulator for each month and station. The shaded blue area shows the range of this mean plus and minus 1 standard deviation, while the blue dashed lines show the minimum and maximum sampled values. The red line shows the MMM results (mean of the years 2011–2015 shown in Fig. 2), and the shaded red area corresponds to the 25% and 75% quartiles. The CCN_0.2_ values obtained from observational data are shown by symbols (mean of the available data).

CCN_0.2_ concentrations in perturbed parameter ensemble (PPE) simulations using HadGEM3-UKCA (Yoshioka et al., 2019) for 2008 at these stations are shown in Fig. 5, together with observations. The solid blue line shows the mean of the sample of 260 000 model variants that cover the multidimensional uncertainty of the PPE (sampled using an emulator). The blue shading shows the range of 1 standard deviation around the mean, and the dotted lines show the minimum and maximum sampled values. The range of 1 standard deviation either side of the mean value represents approximately 68% of all samples, and therefore the blue shading shows approximately the same relative range as for the multi-model comparison in Fig. 2 (25% and 75% quartiles). The MMM averaged for the years 2011–2015 is also plotted in this figure for comparison purposes together with the 25% and 75% quartile shaded area. The means of the available observations from the different years are shown by symbols. Since the interannual variability of simulated MMM CCN_0.2_ concentrations shown in Fig. 2 is generally small compared to inter-model variability, the difference in years between simulations and observations is not considered to undermine the model–data comparisons.

Except for Mace Head, the uncertainty ranges in the PPE are somewhat smaller than the 25% and 75% quartiles of the models shown in Fig. 2. This suggests that model structural differences and the emission inventories used in different models are more important sources of diversity of estimated CCN_0.2_ concentrations for the central 70% range than the fully sampled parametric uncertainty in a single model. However, the maximum–minimum ranges are much larger in the PPE than in the MMM at many locations. Therefore, the values of the sampled model variants from the PPE are more concentrated near the mean but have longer tails on their distribution compared to values from MMM. This is to be expected from such a relatively small sample of models in the MMM.

Model–data comparisons are qualitatively similar to the case with MMM. The PPE simulations underestimate the observed CCN_0.2_ concentrations at many stations and in many months. Exceptions are Puy de Dôme and Hyytiälä where PPE simulations reproduce the observations well for most of the months and Finokalia where, just like MMM, the PPE overestimates the observations. At Melpitz and Vavihill simulations capture the observed values in summer but underestimate them in winter and early spring. The PPE simulations fail to capture the observed peaks in winter and early spring at Mace Head and Cabauw as well. This is unlike the case with MMM, which does not show a distinct wintertime underestimate (Fig. 3). The qualitative agreement between PPE and MMM indicates that the perturbed parameters are those with significant control on aerosol processes and emissions and can be used for CCN uncertainty attribution in Sect. 5.

### 3.3 Particle number concentration and PM_1_ aerosol chemical composition

**Figure 6.**
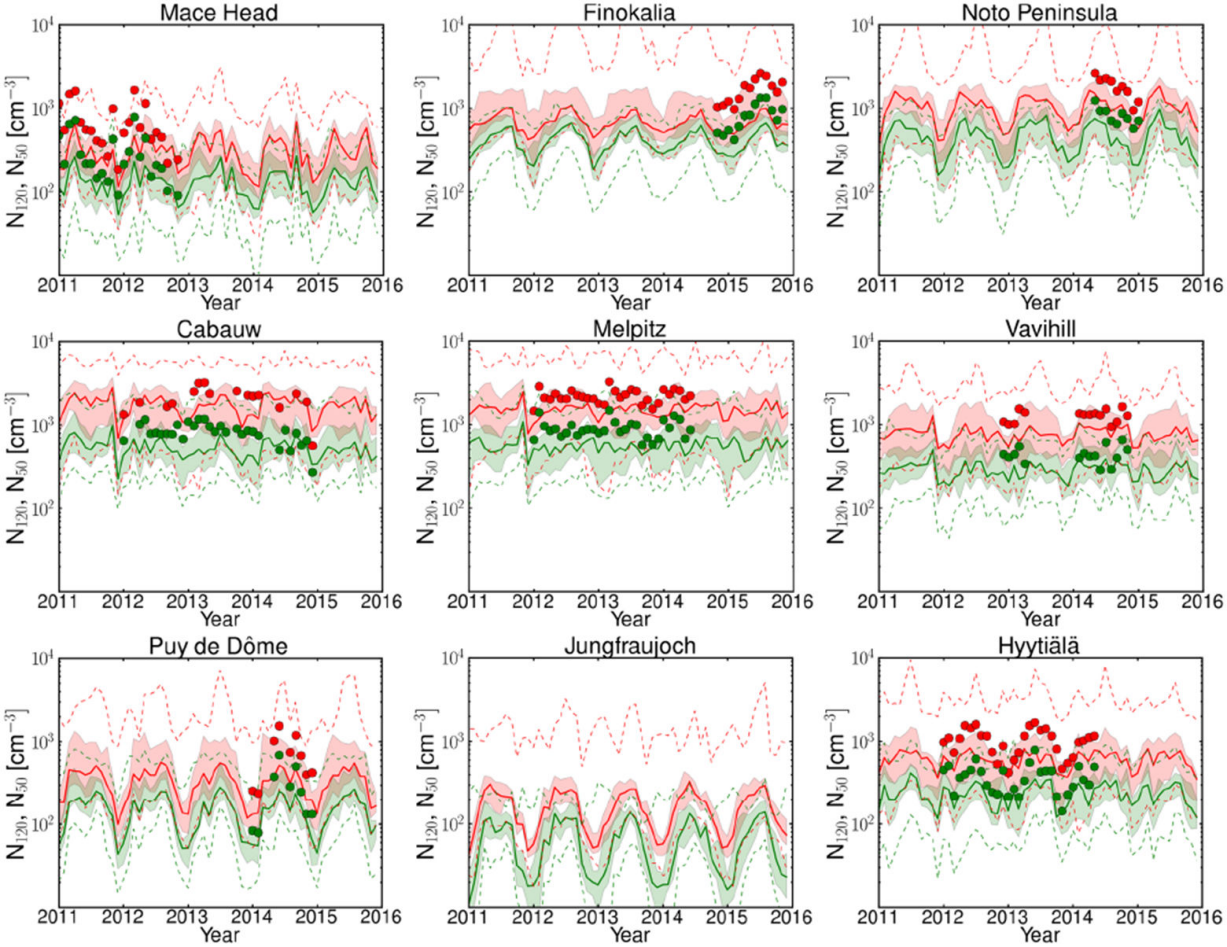
Monthly ensembles for the period 2011–2015 of the number concentration of particles with diameters larger than 50 nm (N_50_ – in red) and 120 nm (N_120_ – in green). The continuous lines correspond to the median of the models for each month; the shaded areas show the 25% and 75% quartiles and the dashed lines the minimum and maximum of all models for the N_50_ (red area) and N_120_ (green area). Observational data are available for all stations except Jungfraujoch and are shown with symbols of the corresponding color.

The observed critical diameter for particle activation into CCN at 0.2% supersaturation at most of the locations in this study is around 100 nm or larger, reaching about 200 nm in spring and summer at Finokalia (Schmale et al., 2018). Therefore, in Fig. 6, the MMMs of the simulated N_50_ and N_120_ are depicted together with the 25% and 75% quartiles of all models that provided station data and are compared with observations. N_120_ is expected to represent a significant portion of the activated particles at 0.2% or higher supersaturation. The MMM underestimates N_50_, and on average NMB is −51% and NME is 55% for all stations. N_80_ is not shown in this figure but follows a similar behavior as N_50_ and N_120_. It is not surprising that in almost all cases both the N_50_ and the N_120_ concentrations are underestimated (the average NMB for MMM for all stations is −50% and the NME is 54%) by a factor that is only slightly lower than the underestimation of the CCN_0.2_ concentration (−50% NMB and 60% NME). It may therefore be concluded that the quantitative differences of the models in the prediction of CCN originate from the underestimation of the number concentration of aerosol particles in the relevant size ranges. Note, however, that the aerosol number concentration cannot be used as a proxy for CCN levels since activation of aerosols to CCN depends not only on the size distribution but also on the chemical composition of the aerosols as well as on the supersaturation that develops in clouds (e.g., Seinfeld and Pandis, 2006; Kalkavouras et al., 2019).

Figure S1 is similar to Figs. 2 and 4 but shows particulate SO4, OA mass in PM_1_ particles at the nine stations, and model results for DU and SS. Strong seasonal variations of the SO4 mass of about 1 order of magnitude are observed and simulated at the alpine site, Jungfraujoch, and at the coastal background stations, Mace Head and Finokalia, although winter minima are overestimated by the models at these coastal sites. Smaller variation or no clear seasonal variation of SO4 is observed at the boreal forest environment of Hyytiälä, the rural background station Cabauw and at the highly polluted Melpitz station during the year. At these three stations, the MMM underestimates the observed annual mean concentration of SO4. Strong seasonal variations of the OA mass are observed and simulated at Mace Head, Finokalia, Jungfraujoch and Hyytiälä, while no distinct seasonal cycle in organic mass is seen at Cabauw and Melpitz. The MMM is underestimating OA concentrations at all sites. The IoA between the MMM and the observations is between 0.28 and 0.62 for all stations. A detailed analysis of each model separately (Table S6) shows that the OA mass concentration is underestimated (mean NMB is −37%) by nine of the models and overestimated by six of them (range of NMB −97% to 216%). Because different models are appearing as outliers at each station, it is difficult to conclude whether the parameterizations in one model are better than another. This, however, is consistent with the findings of a recent OA intercomparison study that considered 31 models (Tsigaridis et al., 2014) and several modeling studies that suggest a missing source of OA needed to reconcile observations with model results (Spracklen et al., 2011; Heald et al., 2011). It appears therefore that in addition to the aerosol number concentration discussed earlier, a possible source of error in the simulation of aerosol and CCN number concentrations in the present study originates from the underestimation of the submicron OA mass at the stations where a significant contribution of the submicron OA mass to the CCN_0.2_ levels has been observed (Schmale et al., 2018). The importance of the contribution of OA to the uncertainty of CCN is also supported by the PPE simulations further discussed in Sect. 5.

### 3.4 CCN persistence

**Figure 7.**
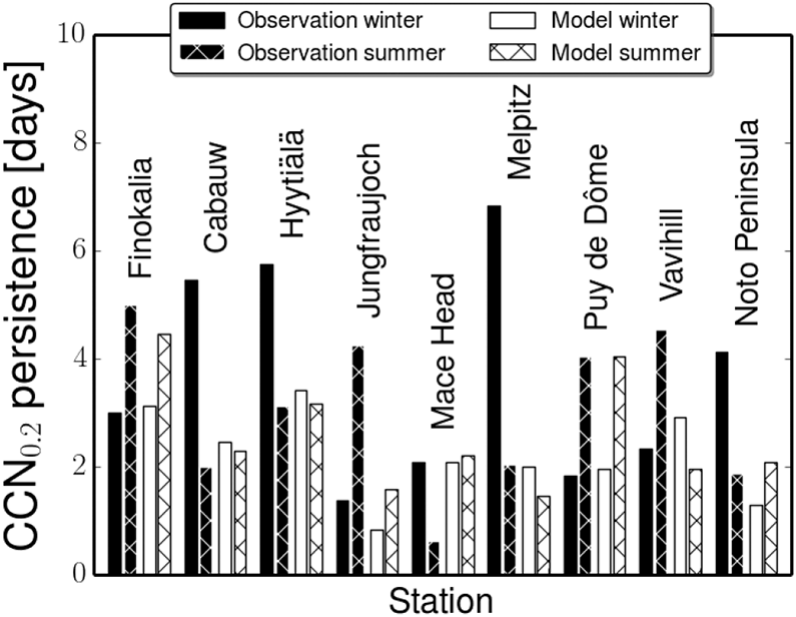
Comparison between the observed and the mean of the model-derived persistence (days) of CCN_0.2_ during winter (left bar) and summer (right shaded bar) for each station. The observed persistence times are shown in black for each station and the mean of the model-derived persistence times in white. The persistence times obtained from model simulations have been computed at the same time periods as the observed ones.

The above analysis of CCN and aerosol number concentrations shows that on average the models are able to simulate the seasonal variations in CCN concentrations, while the model-to-observation differences in the CCN concentrations can be attributed mainly to a systematic underestimation of the number of aerosol particles that are large enough to act as CCN. The ability of models to simulate short-term variations (order of days) of the CCN number concentration is examined based on the calculated persistence of CCN_0.2_ number concentrations during summer and winter (see Sect. 2.4) for all stations and for each model. The average persistence times for all models are compared in Fig. 7 with those derived from the observations (Schmale et al., 2018). Depending on the season and the station, the persistence time varies from a few hours (e.g., summer in Mace Head) to several days (e.g., winter in Melpitz).

Depending on the station, the persistence time is longer during winter (five stations) than during summer (four stations). The average persistence of the CCN_0.2_ number concentrations simulated by the individual models is consistent with the observed change between winter and summer at six among the nine stations. At all stations, the simulations display a much smaller change from winter to summer than indicated by the observations. Furthermore, the modeled change at Mace Head, the Noto Peninsula and Vavihill is opposite to the observed one. For the high-altitude stations, Puy de Dôme and Jungfraujoch, the models calculate longer persistence times during summer than during winter, in agreement with the observations. For these two high-altitude stations, a significant increase in the number concentration of CCN_0.2_ is observed during summer because the stations are subjected to the boundary layer air mass influence during that season, while during winter they are largely in the free troposphere. Therefore, despite the fact that the number concentration of CCN_0.2_ is overall underestimated, the models are able to reproduce the dynamical behavior of these continental background stations, most probably because they are able to simulate the local meteorological changes that drive CCN persistence (Fig. S4 and further discussion in Sect. S3.1).

Analyzing the factors that affect the persistence and then attributing the differences between the observed and the model-derived values to the underlying physical and/or chemical process parameterizations in each model is a demanding task, which is also likely to be model and case dependent. In addition to atmospheric transport patterns, dry and wet deposition processes are presumably affecting the persistence time. Because the present exercise was not focused on the deposition of aerosols, it does not have the necessary elements to elaborate on differences in the results associated with differences in the deposition parameterizations. However, earlier global model comparisons provide insight into such differences. The Tsigaridis et al. (2014) comparison of 31 global models, among which are those participating in the present study, has shown that the representation of aerosol microphysics in the models was important for dry deposition. In particular, they have shown that the use of the M7 aerosol microphysics module was associated with low dry deposition fluxes of organic aerosol, which is mainly fine aerosol in the models, and the dry deposition rate coefficient ranged from 0.005 to 0.13 d^−1^, i.e., with a max/min ratio of 26. They also found that the effective wet deposition rate coefficient in the 31 participating models ranged from 0.09 to 0.24 d^−1^, i.e., with a max/min ratio of 2.6 that is 10 times lower than for dry deposition, and found virtually no change between AEROCOM phase I and AEROCOM phase II models. Kim et al. (2014) compared the deposition of dust, which is mainly coarse aerosol, calculated by a smaller subset (five) of AEROCOM models. They pointed out that the size distribution of dust differs among these models and found a 30% difference in the effective dry deposition rate coefficient and about the same in the total deposition rate varying from 0.28 to 0.37 d^−1^. The Kim et al. (2014) analysis also revealed differences in the annual precipitation rate and in its seasonal distribution in the models, as well as factor of 2 differences in the fraction of wet to total deposition of dust among the models (ranging between 0.36 and 0.63). In addition, the PPE results (see Sect. 5) clearly show that dry deposition is one of the major factors of uncertainty in the calculations of CCN in 0.2% supersaturation. Kristiansen et al. (2016) investigated the causes of differences in aerosol lifetimes within 19 global models by making use of an observational constraint from radionuclide measurements and found largely underestimated accumulation-mode aerosol lifetimes due to removal in most models that is too fast. In particular, they found that the way aerosols are transported and scavenged in convective updrafts makes a large difference in aerosol vertical distribution and lifetimes, as revealed in their simulations from the same model (CAM5) but with different convective transport and wet removal treatments (Wang et al., 2013).

Furthermore, the size of the emitted OA and BC particles has been shown to be an important parameter to which the persistence time and in particular the summer-to-winter ratio of the persistence time of CCN is sensitive (see sensitivity runs performed with one (TM4-ECPL) among the participating models in Sect. S3.1 and Fig. S5). Section 5 further attributes CCN_0.2_ uncertainty to various parameters.

### 3.5 Cloud droplet number concentration from CCN spectra

Inside a cloudy updraft, *s*_max_ is reached when supersaturation generation from expansion cooling becomes equal to its depletion by the condensation of water vapor onto the growing droplets (Nenes and Seinfeld, 2003). Increasing updraft velocity enhances the cooling rate of the cloudy air parcels, which in turn allows for higher supersaturation and eventually increases *s*_max_ and CDNC (*N*_d_ in the following text and figures). Increases in CCN concentrations tend to increase *N*_d_ and associated water vapor depletion in the early stages of cloud formation; this in turn hinders the development of supersaturation and implies an eventual decrease in *s*_max_. This water vapor “competition effect” is especially strong when clouds form in the presence of large, hygroscopic particles such as sea-salt aerosol or large amounts of accumulation aerosol (Morales Betancourt and Nenes, 2014a; Ghan et al., 1998). Competition effects in turn explain why droplet number responses exhibit a sublinear response to modulations in CCN; only when CCN concentrations are very low (or updraft velocities very high) does *s*_max_ become high enough so that the sensitivity of *N*_d_ to CCN approaches unity.

**Figure 8.**
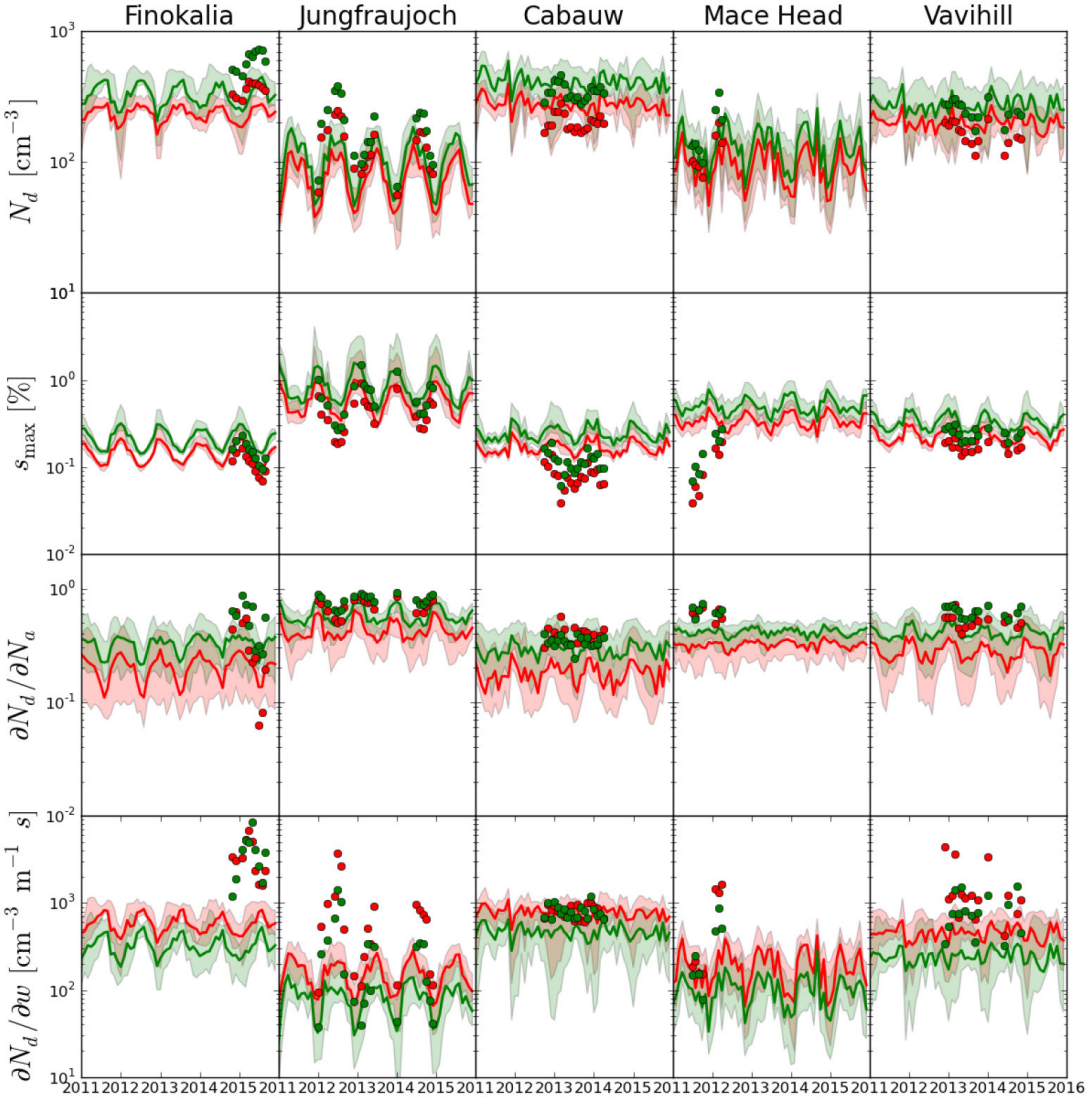
Comparison between the observed (symbols) and the monthly averages of all models (continuous lines) of the cloud droplet properties: in red for updraft velocity *w* = 0.3 ms^−1^ and in green for updraft velocity *w* = 0.6 ms^−1^. For each station from top to bottom the four graphs show (as indicated in the *y* axis label) the number of cloud droplets, *N*_d_, the maximum supersaturation, *s*_max_, the sensitivity of the *N*_d_ to the total number of aerosol particles, (*∂N*_d_/*∂N*_a_), and the sensitivity of the *N*_d_ to the updraft velocity (*∂N*_d_/*∂w*).

Based on the behavior described above, one can understand the *N*_d_ predicted from simulated and observed CCN spectra. This is straightforward for the Jungfraujoch and Mace Head stations. For Cabauw and Vavihill the observed-to-simulated ratio turns from a substantial overestimation in CCN_0.2_ to an underestimation in *N*_d_, and the opposite is found for Finokalia. This can be explained as follows. At both Cabauw and Finokalia, *s*_max_ derived from observations is very low (approaching in the summer 0.07% at Finokalia and 0.04% at Cabauw; Fig. 8). The models overestimate these low values of *s*_max_, and such values are indicative of the presence of large particles (> 250 nm) with sufficient hygroscopicity at these stations that are not captured by the models. Indeed, at Cabauw the available observations of CCN at 0.1% supersaturation show a larger underestimate by the models than for CCN_1.0_ and CCN_0.2_ (Fig. S16), also pointing to a model underestimate of the largest particles (> 250 nm) that induce the very low *s*_max_. The overestimate in *s*_max_ leads to an underestimate in *N*_d_ by the models for all seasons except winter at Cabauw when the models at high updraft velocity capture the observationally derived *N*_d_ levels. Furthermore, at Finokalia, CCN_1.0_ is underestimated year-round, indicating that, in addition to the largest particles, the very small particles (smaller than 50 nm) that activate at 1.0% supersaturation and/or their hygroscopicity are also underestimated by the models there. On the other hand, particles larger than 120 nm that activate at 0.2% supersaturation are overestimated, especially in winter, and slightly underestimated in summer. Therefore, the global models have significant difficulties in capturing the aerosol size distribution and hygroscopicity at Finokalia, which in turn translate into counterintuitive discrepancies in *N*_d_.

At Vavihill a somewhat different behavior is found; the underestimate of CCN at supersaturations of 0.2% and 0.7% changes to an overestimate at supersaturation 0.1% mainly in summer (Fig. S16), indicating an underestimate of fine particles and/or their hygroscopicity and an overestimate of the largest particles and/or their hygroscopicity, in particular during summer. This agreement of model results with observations during winter and the overestimate of CCN at 0.1% supersaturation during summer can explain the similar behavior of modeled *N*_d_.

The difference between model and observationally derived *∂N*_d_/∂*w* clearly supports the above statements. Since observations predict a suppressed *s*_max_ compared to model distributions (Fig. 8), water vapor competition effects in the observations are much more severe than in the model, indicating that observations are much more (positively) sensitive to updraft velocity. The opposite trends are seen for activation fraction (*∂N*_d_/*∂N*_a_), given that reductions in aerosol reduce competition effects. The reduced water vapor competition effects at higher updraft velocities and the trend in CCN error also generally explain why the sensitivities are smaller for the highest updraft velocity.

**Figure 9.**
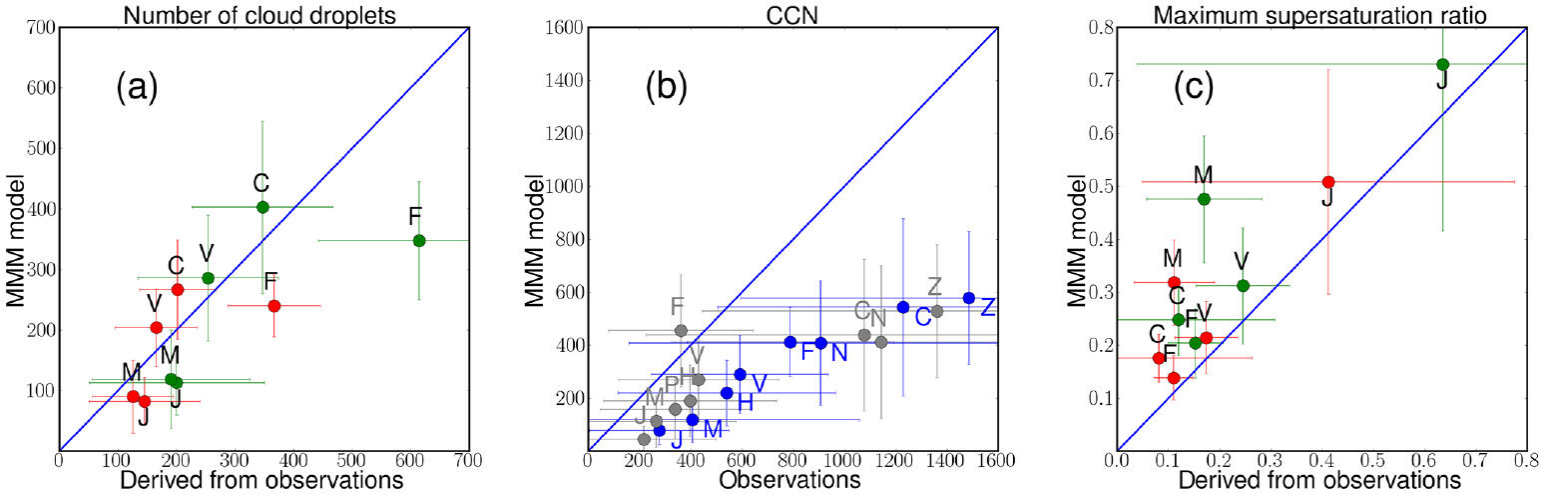
Scatter plot of the average of multi-model median results (*y* axis) versus observationally derived results (*x* axis) for **(a)** CDNC (*N*_d_) (cm^−3^; in red for updraft velocity *w* = 0.3 ms^−1^ and in green for updraft velocity *w* = 0.6 ms^−1^); **(b)** CCN at supersaturation 0.2% (gray) and CCN at maximum supersaturation (blue) with available data (cm^−3^). To fit the scale all CCN number concentrations at maximum supersaturation (blue symbols) have been divided by 2. Panel **(c)** is as panel **(a)** but for *s*_max_ (%). The letters close to the symbols indicate the station names (C – Cabauw, F – Finokalia, H – Hyytiälä, J – Jungfraujoch, M – Mace Head, N – Noto Peninsula, P – Puy de Dôme, V – Vavihill, Z – Melpitz).

As expected, both *s*_max_ values and *N*_d_ for all observations and simulations are higher for *w* = 0.6 ms^−1^ than for *w* = 0.3 ms^−1^. The response of *s*_max_ and *N*_d_ to increasing *w* also depends on the activated fraction (Fig. 8 third row). The calculated *N*_d_ increases progressively from the low values seen for the clean marine conditions at Mace Head and the high alpine atmospheric conditions of Jungfraujoch to the rural background conditions at Cabauw and Vavihill, while at Finokalia the observationally derived *N*_d_ values are the largest among the five stations (Fig. 9a). At Jungfraujoch, Finokalia and Mace Head, the seasonal variability of *N*_d_ is captured, despite the fact that the multi-model median tends to underestimate the observationally derived *N*_d_. However, the individual models show both overpredictions and underpredictions of the observations (Fig. S3). Owing to the water vapor competition effect, *s*_max_ decreases for increasing *N*_d_, meaning that clouds at a given location do not have a “characteristic *s*_max_”, but rather depend on the given set of aerosol and dynamical conditions that develop during the cloud formation.

For all stations except Finokalia, the agreement between the model and observationally derived *N*_d_ (Fig. 8) tends to be better than for CCN (Figs. 2, 4) and aerosol number concentrations (Fig. 6) (as expressed by the MMM’s NMB and NME for all stations provided in Table S6). Indeed, for all stations except Finokalia, NMB and NME of the MMM for *N*_d_ vary from −7% to −17% and 41% to 42%, respectively, with the lowest values calculated for the low updraft velocity. For CCN_0.2_ NMB is −59% and NME 63%, averaged over the same stations. This trend is a result of the competition effect of CCN on *s*_max_; if observed CCN concentrations are higher than predicted, then the “observed” *s*_max_ tends to be less than the “predicted” *s*_max_, which means the discrepancy in observed and predicted *N*_d_ is reduced compared to the CCN errors. The error reduction is substantial, especially under lower updraft velocity conditions. As a qualitative example we present here the ratio of the observed to the simulated average values of CCN_0.2_ number concentrations: 4.0 at Jungfraujoch, 2.2 at Cabauw, 2.1 at Mace Head, 1.5 at Vavihill and 0.8 at Finokalia (Fig. 3). In the case of *N*_d_ the corresponding ratios for *w* = 0.6 ms^−1^ are ~ 1.8 at Jungfraujoch, ~ 0.9 at Cabauw, ~ 1.5 at Mace Head, ~ 0.9 at Vavihill and ~ 1.8 at Finokalia (Fig. 9). All these ratios are inversely correlated with the observed to the simulated average values of *s*_max_ (Fig. 9), a clear indication of competition effects on *N*_d_ and prediction error mitigation.

In agreement with our finding, Sotiropoulou et al. (2006) used a similar approach applied to observations from the ICARTT field campaign and estimated that a 20%–50% error in CCN closure results in a 10%–25% error in *N*_d_, while global simulations suggest global average CCN prediction error between 10% and 20% and a smaller corresponding *N*_d_ error between 7% and 14% (Sotiropoulou et al., 2007). Such a reduction in error can be explained by self-regulation by *N*_d_ since *s*_max_ decreases with increasing aerosol number concentration, as discussed by many studies published to date (e.g., Twomey et al., 1959; Charlson et al., 2001; Nenes and Seinfeld, 2003; Feingold and Siebert, 2009), giving rise to regions where *N*_d_ is relatively insensitive to changes in CCN or updraft velocity (e.g., Rissman et al., 2004; Reutter et al., 2017). At very high CCN levels and in the presence of sufficiently large hygroscopic CCN, *N*_d_ may actually decrease with increases in aerosol amount (Ghan and Abdul-Razzak, 1998; Feingold, 2001; McFiggans et al., 2006; Reutter et al., 2017); parameterizations that do not fully capture these important aspects of the aerosol–droplet relationship may also give rise to biases in aerosol indirect forcing assessments (e.g., Morales-Betancourt et al., 2014a).

These results clearly indicate that the number of CCN at a prescribed supersaturation cannot be used as an indicator for the number of activated droplets. The maximum supersaturation that develops inside the cloud (hence droplet number) responds to changes in aerosol and vertical velocity levels and is thus dynamically determined and can vary considerably for a given site. This is even further complicated by the potential for model biases to change sign at cloud-relevant supersaturations. CCN-derived comparisons cannot even be used qualitatively, as the supersaturation levels can be so different from a prescribed value that even the error trend in *N*_d_ may not be reflected. For example, according to observationally derived data, CCN_0.2_ at Cabauw is significantly higher than at Finokalia, although at Finokalia *N*_d_ is larger for the observations but not for the model results. Our analysis, however, clearly shows that the models examined here do not exhibit the same level of *N*_d_ prediction error as CCN error – a robust trend that is a result of the physics of cloud droplet formation. Because of the discrepancy in the sensitivities *∂N*_d_/*∂N*_a_ and *∂N*_d_/*∂w*, models may be predisposed to be too “aerosol sensitive” or “aerosol insensitive” in aerosol–cloud–climate interaction studies, even if they may capture average droplet numbers well. This is a subtle but profound finding that only the sensitivities can clearly reveal and may explain inter-model biases on the aerosol indirect effect. Few published efforts (apart from Morales Betancourt and Nenes, 2014a, and Sullivan et al., 2016) can demonstrate this, none over a range of models and using a considerable aerosol dataset for evaluation as performed here.

## 4 Global distributions of surface CCN_0.2_ and particle number concentrations

**Figure 10.**
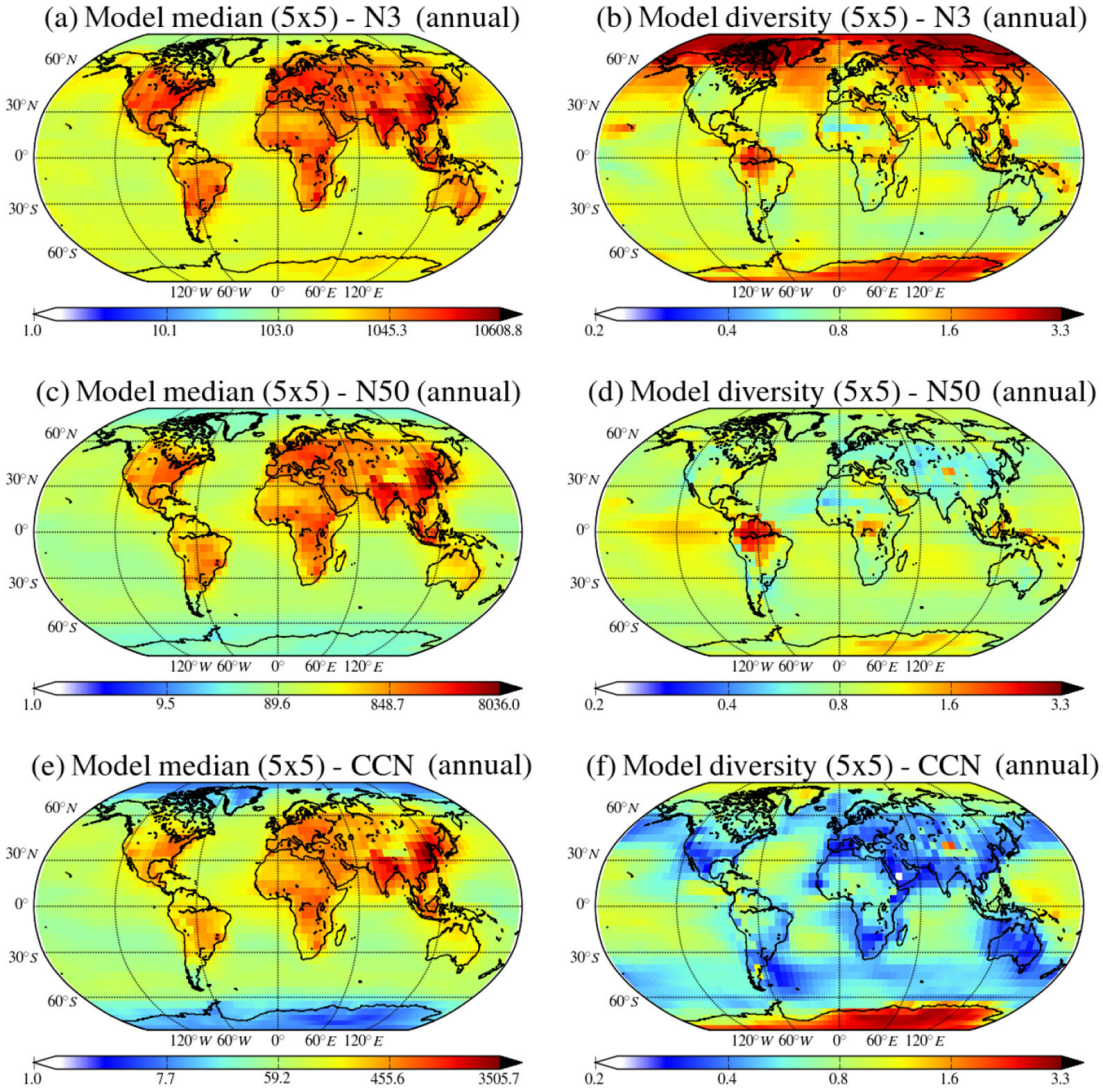
Global distributions of the annual multi-model median concentrations of N_3_, N_50_ and CCN_0.2_ (cm^−3^) for the year 2011 (**a, c, e**, respectively) and the corresponding diversities (**b, d, f**, respectively; calculated as the ratio of standard deviation to the mean of the models).

The global near-surface annual mean MMM distributions of the N_3_, N_50_ and CCN_0.2_ number concentrations for the year 2011 (Fig. 10) show similar patterns, i.e., larger concentrations over the continents due to the primary anthropogenic emissions over industrialized areas in the USA, Europe and Asia, as well as dust and biomass burning emissions in the tropics.

Multi-model median near-surface N_3_ number concentrations over continental regions vary between 1000 and 10 600 cm^−3^, while over the marine boundary layer (MBL) they vary between 100 and 2000 cm^−3^, rarely exceeding 300 cm^−3^ (Fig. 10a). The MMM N_3_ surface distribution is similar to the results by Spracklen et al. (2010) and Gordon et al. (2017), who computed maximum N_3_ concentrations of ~ 10 000 cm^−3^. The concentration of N_3_ is directly related to new particle formation and growth as well as to primary emitted particles. Since models use different nucleation mechanisms and emission inventories it is expected that the diversity of the model results is higher for N_3_ than for particle number concentrations with a larger (low-end) cutoff diameter. The largest diversities in the model results (Fig. 10b) are found in the polar regions, where concentrations are relatively low, and in the continental boundary layer with high values (about 2) observed in the tropics and particularly in South America and over the boreal regions in Asia. Diversities of up to 1.5 are computed for the Mediterranean, Arabian Peninsula, Central Africa, Indonesia and Southeast Asia, indicating differences between models in the representation of primary and secondary aerosol sources in these regions. Over the oceans the diversity is lower (< 1) except in the high latitudes of the Northern Hemisphere where it exceeds 1.5. Even lower model diversity (around 0.8) is found in highly polluted areas over North America and Europe, indicating consistency between models in the representation of aerosols in these regions. In addition to new particle formation, our results point mainly to biomass burning emissions as a major source of uncertainty in the model calculations, resulting in high model divergence in areas like southern Europe, tropical Africa and America, southern Asia and Indonesia. Assumption of emission injection height is also a source of discrepancy between models, leading to differences in the calculated lifetimes (up to 30%) and in the tropospheric columns (up to 25%) of pollutants (Daskalakis et al., 2015), while differences of an order of magnitude in their concentrations are computed for the middle troposphere (Jian and Fu, 2014). Thus, differences in the emission injection heights in the participating models, as outlined in Sect. 2.1 and Table S4, contribute to the model result divergence. The highest maximum N_3_ concentrations in a 5° × 5° grid box (Fig. S6) were computed by the GISS-E2.1-MATRIX model (~ 176 000 cm^−3^) and the TM4-ECPL model (~ 102 000 cm^−3^), while the lowest were from the ECHAM6_HAM2-AP model (~ 6400 cm^−3^). A sensitivity simulation was performed by a single model (TM4-ECPL; discussed in Sects. 3.3 and S3.1 and Fig. S5) assuming the same primary emissions of carbonaceous aerosol in terms of mass to be emitted at larger particle sizes. This additional simulation shows the importance of the assumptions on size distribution of the emissions in the models since the results of this simulation are very close to the average of the other models. In agreement with these findings, Spracklen et al. (2010) concluded that the sensitivity of N_3_ to the size of emitted particles originating from anthropogenic activities is significantly higher in regions close to anthropogenic sources and significantly lower at remote boundary layer sites.

The annual global mean distribution of near-surface N_50_ particle number concentrations (Fig. 10c) is similar to that of the N_3_ particles, but the number concentrations are lower for these larger particle sizes that are more relevant for CCN. The spatial distributions of N_50_ are similar, but their concentrations are reduced by about a factor of 2.5 compared to N_3_. The highest values of N_50_ are found over or close to industrialized regions due to anthropogenic emissions and over Central Africa and South America due to strong biomass burning emissions. Over marine regions, N_50_ is higher in the Northern Hemisphere than in the Southern Hemisphere due to the outflow from continental anthropogenic sources. Despite the similarities of the global MMM distributions, the models’ diversity and spatial pattern of N_50_ (Fig. 10d) differ significantly from that of N_3_. Excluding polar regions as for N_3_, the highest model diversities for N_50_ (~ 2) are observed in regions with strong biomass burning emissions (southern America, Central Africa and Indonesia), and high diversities are also found over the tropical Pacific, which might be associated with marine emission representation in the models. In all other regions the diversity of N_50_ simulations does not exceed 1, even over the remaining tropical and southern oceans where sea salt is important.

The near-surface MMM concentrations of CCN_0.2_ do not exceed 3500 cm^−3^ over polluted areas in Europe, Asia and the United States, as shown in Fig. 10e. This value is in the range of the 3162–10 000 cm^−3^ CCN_0.2_ concentrations simulated by Spracklen et al. (2011) over China and attributed to carbonaceous aerosols acting as CCN. In the present study, only one model (EMAC) computes CCN_0.2_ levels that exceed 10 000 cm^−3^ over the Taklimakan Desert in Asia, while the remaining 14 models show maximum surface CCN_0.2_ concentrations < 5000 cm^−3^ (see Fig. S9). The surface distribution and magnitude of CCN_0.2_ are similar to N_120_ (Fig. S8), with the maximum CCN_0.2_ concentrations only slightly lower than the N_120_ values for most models, indicating that most of the N_120_ particles activate, implying a global mean kappa of ~ 0.2 for 120 nm particles. However, analysis of the individual model results over the polluted areas shows that the number concentration of N_120_ can, in most cases, be either 50% lower or higher than that of CCN_0.2_. The modeled CCN_0.2_ diversity is lower than the diversity for N_50_ with values < 0.5 for midlatitude continental regions and around 1 over the tropical oceans, where the CCN_0.2_ number concentration is usually lower than 60 cm^−3^, but also over tropical southern Africa and Central Africa where CCN_0.2_ number concentration is a few hundred cubic centimeters. CCN_0.2_ model diversity is also lower than that of N_3_ simulations. The maximum reduction of the model diversity in CCN_0.2_ simulations compared to that in N_3_ simulations is found to exceed a factor of 9 and maximizes over the high latitudes of the Northern Hemisphere and the south Arabian Peninsula where new particle formation is high. Overall, a global mean reduction of a factor of about 2 is found, as shown in Fig. S18, that provides the ratio of N_3_ model diversity (Fig. 10b) over CCN_0.2_ model diversity (Fig. 10f).

Some of the differences in global near-surface distributions of CCN (Fig. S9) can be associated with the corresponding differences in the computed SO4 and OA surface distributions (Figs. S10 and S11, respectively). For instance, in China and South America, models that are biased low in SO4 and high in OA are also biased low in CCN. Significant differences are also found for black carbon, sea salt and dust PM_1_ components (Figs. S12-S14). In particular, for all models near-surface BC distributions maximize over China, while individual models differ by a factor of 3 to 4. Simulated SS distributions maximize over the southern oceans where the models show the largest differences of up to 2 orders of magnitude, reflecting large differences in the parameterized emissions of SS (see also Table S3). Finally, DU distributions show the largest spread among models with near-surface values that differ by up to a factor of 40. The global surface distributions of the MMM of the chemical compound (SO4, BC, OA, SS and DU) concentrations that contribute to PM_1_ are shown in Fig. S15 (left column) together with the corresponding model diversities (right column). For all simulated PM_1_ components diversities maximize south of 60° S and north of 60° N, similarly to N_3_, which reflects the challenges of the models in simulating atmospheric transport, deposition and chemistry close to the poles.

## 5 Causes of uncertainty in CCN

In this section we use the HadGEM-UKCA perturbed parameter ensemble (PPE) to identify some potential causes of model diversity and bias compared to the observations. We performed a variance-based sensitivity analysis at each measurement site using the 260 000 HadGEM-UKCA model variants sampled from the emulator following the methodology described in previous studies (Lee et al., 2013; Johnson et al., 2018).

**Figure 11.**
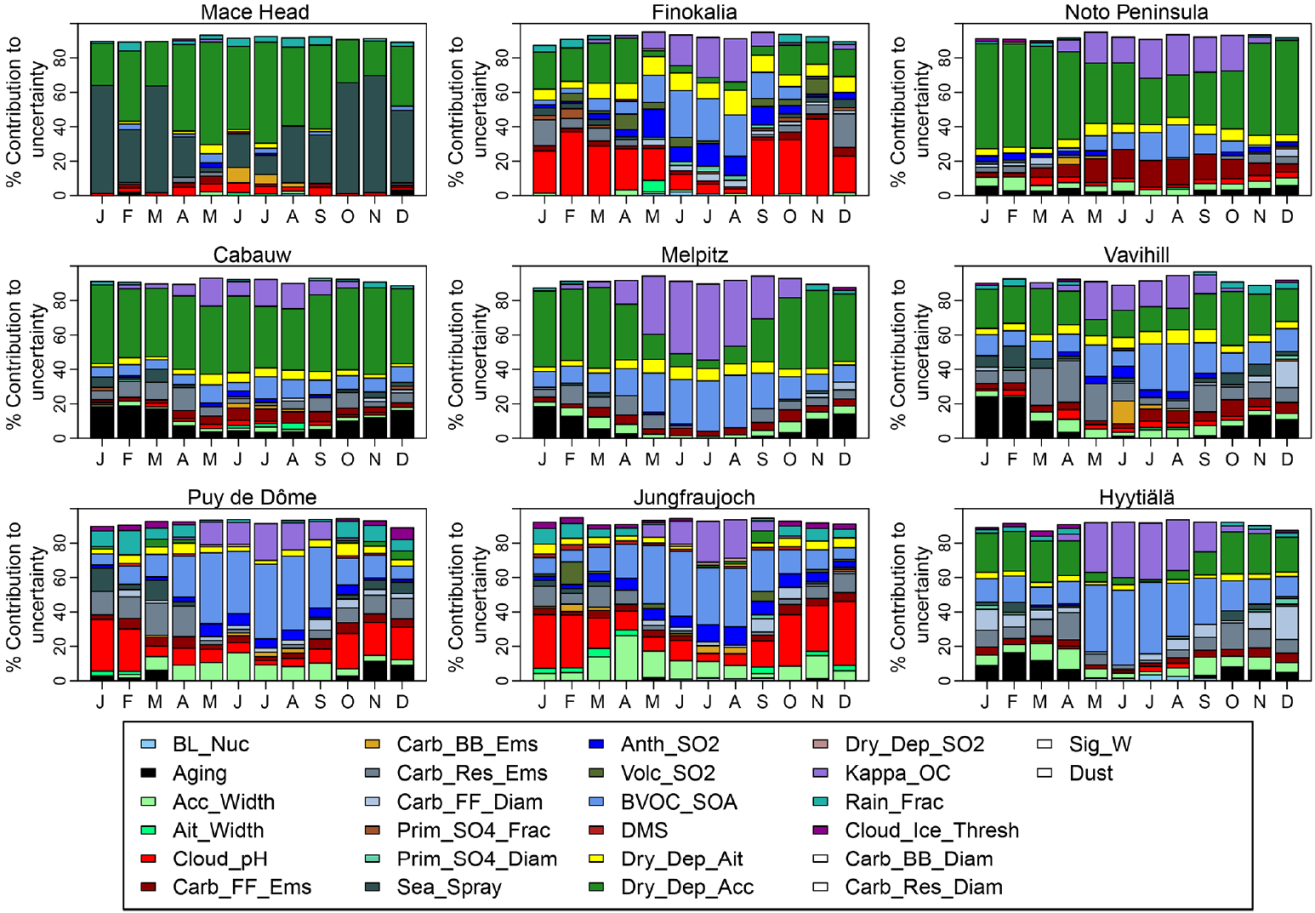
Contribution to the uncertainty in monthly average CCN_0.2_ based on HadGEM3-UKCA perturbed parameter ensemble simulations for the year 2008. Each color refers to 1 of the 26 perturbed parameters as indicated in the legend of the figure. The uncertainty is shown as the percentage contribution of the parameter to the CCN_0.2_ variance. The assumed parameter uncertainty ranges are given in Yoshioka et al. (2019). All contributions smaller than 1% are not shown. Abbreviations are as follows. BL_Nuc: boundary layer nucleation; Aging: aging “rate” from insoluble to soluble; Acc_Width: modal width (accumulation soluble–insoluble); Ait_Width: modal width (Aitken soluble–insoluble); Cloud_pH: pH of cloud drops; Carb_FF_Ems: particle mass emission rate for BC and OC (fossil fuel); Carb_BB_Ems: particle mass emission rate for BC and OC (biomass burning); Carb_Res_Ems: particle mass emission rate for BC and OC (biofuel); Carb_FF_Diam: particle emitted mode diameter for BC and OC (fossil fuel); Carb_BB_Diam: particle emitted mode diameter for BC and OC (biomass burning); Carb_Res_Diam: particle emitted mode diameter for BC and OC (biofuel); Prim_SO4_Frac: mass fraction of SO_2_ converted to new ${\text{SO}}_{4}^{-2}$ particles in sub-grid power plant plumes; Prim_SO4_Diam: mode diameter of new sub-grid ${\text{SO}}_{4}^{-2}$ particles; Sea_Spray: sea spray mass flux (coarse / accumulation); Anth_SO2: SO_2_ emission flux (anthropogenic); Volc_SO2: SO_2_ emission flux (volcanic); BVOC_SOA: biogenic monoterpene production of SOA; DMS: DMS emission flux; Dry_Dep_Ait: dry deposition velocity of Aitken mode aerosol; Dry_Dep_Acc: dry deposition velocity of accumulation-mode aerosol; Dry_Dep_SO2: dry deposition velocity of SO_2_; Kappa_OC: hygroscopicity parameter kappa for organic aerosols. Default value in UKCA is 0.06; see Petters and Kreidenweis (2007). Sig_W: standard deviation of updraft velocity (this affects the activation of aerosol particles to form cloud droplets). Dust: dust emission flux; Rain_Frac: the fraction of the cloudy part of the grid box in which rain is forming and hence scavenging takes place; Cloud_Ice_Thresh: scavenging (by both cloud liquid and ice water) is suppressed in dynamic clouds when cloud ice fraction is higher than this value. The parameters with no color in the legend do not contribute to the uncertainty in CCN_0.2_ (less than 1 %) at any station in any month.

Figure 11 shows the fraction of variance in CCN_0.2_ that can be attributed to each of the perturbed parameters. Here we draw attention to the main parameter effects and refer to Yoshioka et al. (2019) for a full description of all parameters. The list of these parameters is provided in the caption of Fig. 11. In the summer, the largest contributions to uncertainty in CCN_0.2_ at most sites come from the biogenic volatile organic compound (BVOC) emission flux and the assumed hygroscopicity of the organic matter in the particles (*κ*_OA_). The BVOC emissions in this model are assumed to be *α*-pinene and to have an uncertainty range of 12–225 Tg SOA production per year. The *κ*_OA_ is assumed to have a range of 0.1–0.6 and to be fixed during the simulation time (i.e., the hygroscopicity does not change due to within-particle oxidation). Together, these two mostly biogenic-related parameters account for up to 90% of the CCN variance in summer, ranging from about 0% at Mace Head, 20% at Cabauw, 40% at Finokalia and 70% at Melpitz to 90% at Hyytiälä. These results show that at Hyytiälä the organic fraction of CCN-active aerosol is highest, while at other locations, like Mace Head, the inorganic fraction dominates the total hygroscopicity. Except at the Mace Head coastal site, the other important parameters in summer are dry deposition of aerosol, anthropogenic SO_2_ emissions (at Finokalia, Puy de Dôme and Jungfraujoch), the fossil fuel emission flux (at the Noto Peninsula, Cabauw and Melpitz) and the assumed width of the accumulation mode (at Jungfraujoch and Puy de Dôme).

In winter, aerosol dry deposition is an important cause of uncertainty in CCN_0.2_ at all sites except Jungfraujoch and Puy de Dôme. At most sites (except Mace Head and the Noto Peninsula) the emissions fluxes (and the assumed particle sizes) of carbonaceous aerosol from fossil fuel and residential combustion sources account for 10%–20% of the uncertainty. Aging of aerosol through the uptake of sulfuric acid and SOA is also important at these sites. Finally, the production of sulfate through in-cloud oxidation by ozone (perturbed parameter marked as “Cloud pH”) accounts for 30%–40% of the uncertainty at Finokalia, Puy de Dôme and Jungfraujoch.

In summary, the PPE results suggest that the production of SOA from biogenic emissions combined with the hygroscopic properties of the OA should be investigated as a source of differences in predicted CCN between models in summer. In winter, dry deposition, aging and in-cloud sulfate production are the dominant sources of CCN uncertainty. Given that the importance of CCN prediction uncertainty may not always translate to CDNC uncertainty – especially if cloud formation occurs in a velocity-limited regime – any future analysis should place CCN uncertainty within the context of CDNC uncertainty.

## 6 Summary and conclusions

Within the BACCHUS–AEROCOM multi-model CCN intercomparison initiative, a total of 16 global aerosol–climate and chemistry transport models were compared to each other and to observations. Among them 14 provided results for particle and CCN number concentrations and PM_1_ component mass concentrations, which have been compared to surface observations at eight sites in Europe and one in Japan to evaluate the skill of the simulations.

In this inter-model comparison, models used different meteorology and emissions (e.g., CMIP5 and 6), as well as datasets and parameterizations. Most models (including the multi-model median) tend to underestimate the observed aerosol number concentrations N_50_, N_80_ and N_120_, as well as the CCN concentrations, suggesting an incomplete understanding of the underlying processes. In particular, emissions and the size distribution of emitted particles, injection heights of biomass burning emissions, atmospheric aging and particularly aqueous-phase chemistry, the hygroscopicity of organic aerosol, and dry and wet deposition have been pointed out as main sources of uncertainties in model simulations. Models are, however, reproducing between 45% and 86% of the seasonal variability of N_50_, N_80_, N_120_ and CCN_0.2_ number concentrations, as well as SO4 and OA PM_1_ component mass concentrations, with the exception of Hyytiälä where only 36% of the SO4 variability is captured by the MMM, as indicated by the correlation coefficient of the MMM with the observations (Table S6). While models have improved since the 2014 AEROCOM organic aerosol intercomparison (Tsigaridis et al., 2014), most continue to underestimate the organic submicron aerosol mass concentrations. Thus, the MMM underestimates observed OA PM_1_ mass concentrations by 36% (for Hyytiälä) to 77% (for Jungfraujoch).

The simulated N_3_ number concentrations, which are generally higher over land, show high diversity among models over the Northern Hemisphere continents, while the simulated CCN are less diverse. Overall, a global mean reduction of a factor of about 2 is found in the model diversity in CCN_0.2_ simulations compared to that in N_3_ simulations, maximizing over regions where new particle formation is important. This finding points to differences in the size distribution of primary emissions and/or in the formation and growth of new particles as important sources of the inter-model diversity in CCN.

CCN number concentrations are generally underestimated at all supersaturations by the MMM by at least 34% (Fig. 9, Table S6), with the exception of very low supersaturations, indicating that models have most difficulty in capturing the largest particles (> 250 nm) that activate at very low supersaturations. There is no model that performs best at all stations. The models on average qualitatively capture the strong seasonal variabilities of CCN observed at Finokalia, the Noto Peninsula, Puy de Dôme and Jungfraujoch, as well as the very weak seasonality observed at the other stations. The production of SOA from biogenic emissions combined with the hygroscopic properties of the OA in summer and dry deposition, aging and in-cloud sulfate production in winter have been identified by PPE simulations as dominant sources of CCN uncertainty and should be investigated in the future.

The short-term variability of CCN_0.2_ at the measurement sites has been examined by comparing the CCN_0.2_ persistence time computed from the observed data and the model results. Because persistence time is a normalized timescale driven by the processes that “set” the CCN concentrations, it is more sensitive to air mass changes and the formation–removal rates of atmospheric particles than to the exact number concentration of CCN. With the exception of two models that estimate very large persistence times (about 16 d) during summertime at Finokalia, the modeled persistence times of near-surface CCN_0.2_ are between 0.5 and 9 d depending on the model, location and season (Fig. S4), a range similar to that derived from observations that vary between about 0.5 and 7 d. At six out of nine stations the average relative change in modeled persistence time between winter and summer is in agreement with observations. These persistence times of CCN_0.2_ are sensitive to assumptions on the size of the emitted particles, as shown by a sensitivity simulation with the TM4-ECPL model.

A novel aspect of this study is the comparison of ensemble global aerosol climate model near-surface results with experimentally derived CDNC from surface measurements of CCN at different levels of supersaturation. Note that CDNC is not calculated by each participating model, but a common methodology has been followed to derive the CDNC from the modeled and observed CCN spectra. Despite the large differences between models and observations found in the number concentration of aerosol particles and CCN, the CDNC estimates based on the CCN spectra are in significantly better agreement than the CCN for the stations examined here. In addition, the inter-model spread of CDNC is smaller than that of particle and CCN number concentrations. These trends are robust and a result of the physics of cloud droplet response to aerosol perturbations and show self-regulation by CDNC.

As for CCN number concentrations, in several cases models underestimate CDNC when compared to the observationally derived CDNC (Sect. 3.5). At high aerosol number concentrations, the maximum supersaturation is computed to be low, limiting the fraction of particles that can activate and form CDNC. As a result, the sensitivity of CDNC to updraft velocity prevails. In contrast, at high updraft velocities, CDNC is controlled by the variability in the aerosol number concentration. An anticorrelation is found between the sensitivity of CDNC to the number of aerosols and that to the updraft velocity, showing that the variability of these two parameters can explain the variability in CDNC and limit CDNC formation.

Our results are in agreement with previous studies showing that CDNCs are sensitive to the uncertainties in the CCN number concentrations, mainly in regions where aerosol number concentrations are low and support the concept of the existence of two distinct regimes (“aerosol limited” and “updraft limited”). Unlike previous studies, however, we show that for a large number of models, persistent and substantial CCN prediction biases are considerably reduced when expressed as droplet number concentrations for boundary-layer-type clouds. Biases in CDNC are found to be qualitatively different from the biases in CCN_0.2_ and are attributed to the ability of models to capture the levels of the largest particles that activate at very low cloud-relevant supersaturations. These results point to the need for observations that cover the CCN spectra down to very low supersaturation levels and demonstrate that model–observation comparisons of CCN at a prescribed supersaturation may be misleading in the error evaluation for CDNC, since supersaturation is dynamically determined and can vary considerably for a given site. The methodology proposed here, however, overcomes this limitation and considers the dynamic nature of supersaturation adjustment to CCN variations, thus determining appropriate supersaturation levels for model–observation comparison. Such a methodology can help better guide modeling efforts to focus on regions where CDNC predictions are most biased and sensitive to CCN perturbations (e.g., in the southern oceans).

## Supplementary Material

Supplementary text

Supplementary figures

Table-S6

## Figures and Tables

**Table 1.** Hygroscopicity parameters used by the participating models for water uptake calculations.

Model	SO4	OA	SS	DU	BC	NO_3_
CAM5-Chem-APM	0.9	0.1	1.28	0	0	0.9
CAM5-Chem-ATRAS2	0.61	0.1	1.16	0.001	1 × 10^−6^	0.61
CAM5_MAM3	0.507	0.1	1.16	0.068	0	N/A
CAM5_MAM4	0.507	0	1.16	0.068		N/A
CAM5.3-Oslo	0.507^(1)^	0.14	1.2	0.069	5 × 10^−7^	N/A
ECHAM5.5-HAM2-ELVOC_UH	0.6	0.06	1.12	0		
ECHAM6-HAM2^(2)^	0.7	0	1.3	0	0	N/A
ECHAM6-HAM2-AP^(2)^	0.7	0	1.3	0	0	N/A
EMAC^(3)^		0.1	1.12	0	0	N/A
GEOS-Chem-APM	0.9	0.1	1.28	0	0	0.9
GEOS-Chem-TOMAS	1.0	0.1^(4)^	1.2	0.01	0	N/A
GISS-E2.1-MATRIX	0.507	0.141	1.335	0.14	5 × 10^−7^	0.507
GISS-E2-TOMAS	0.7	0.15^(5)^	1.3	0	0	N/A
TM4-ECPL	0.6	0.1	1.0	0	0	N/A
TM5	0.6	0.1	1.0^(6)^	0	0	0.6

1 In CAM5.3-Oslo the hygroscopicity parameters *κ* for pure ammonium sulfate or sulfuric acid are 0.507 and 0.534, respectively. For internal mixtures, *κ* is a mass-weighted average of the aerosol components, except for particles coated (> 2 nm) with SO4, OA and/or SS, where *κ* is a mass-weighted average of the components of the coating (Kirkevåg et al., 2018).

2 ECHAM6-HAM2 and ECHAM6-HAM2-AP use the Abdul-Razzak and Ghan (AR-G) activation scheme (Abdul Razzak and Ghan, 2000). The reported values are approximated using the number of ions and osmotic coefficients used in the AR-G scheme.

(3) EMAC model simulates the effective hygroscopicity parameter *κ* of each aerosol size mode in order to describe the influence of chemical composition on the CCN activity of aerosol particles (Pringle et al., 2010). These values are the internally mixed *κ* calculated across the nucleation, Aitken, accumulation and coarse modes. The effective aerosol hygroscopicity parameter *κ* is calculated according to the simple mixing rule proposed by Petters and Kreideweis (2007) using the volume fraction and hygroscopicity parameter of each chemical component (23 salts from ISORROPIA-II and 4 bulk species) taken from Petters et al. (2007) and Sullivan et al. (2009)

(4) for hydrophilic OA *κ* = 0.1, for hydrophobic OA *κ* = 0.01 and

(5) for hydrophilic ORG (Lee et al., 2015). For hydrophobic, *κ* = 0.

(6) for NaCl *κ* = 1, for Na_2_SO_4_ *κ* = 0.95. N/A: not considered in this study.

## Data Availability

The data used for this study are available online at https://doi.org/10.5281/zenodo.3265866 (Fanourgakis et al., 2019) (contact: Maria Kanakidou at mariak@uoc.gr). CDNC analysis tools for model and observational data can be obtained from athanasios.nenes@epfl.ch upon request.

## References

[R1] Abdul Razzak H and Ghan SJ: A parameterization of aerosol activation: 2. Multiple aerosol types, J. Geophys. Res.-Atmos, 105, 6837–6844, 10.1029/1999JD901161, 2000.

[R2] Adams PJ and Seinfeld JH: Predicting global aerosol size distributions in general circulation models, J. Geophys. Res.-Atmos, 107, 1–23, 10.1029/2001JD001010, 2002.

[R3] Barahona D and Nenes A: Parameterization of cloud droplet formation in large-scale models: Including effects of entrainment, J. Geophys. Res.-Atmos, 112, 1–14, 10.1029/2007JD008473, 2007.

[R4] Barahona D, Molod A, Bacmeister J, Nenes A, Gettelman A, Morrison H, Phillips V, and Eichmann A: Development of two-moment cloud microphysics for liquid and ice within the NASA Goddard Earth Observing System Model (GEOS-5), Geosci. Model Dev, 7, 1733–1766, 10.5194/gmd-7-1733-2014, 2014.

[R5] Bauer SE, Wright DL, Koch D, Lewis ER, McGraw R, Chang L-S, Schwartz SE, and Ruedy R: MATRIX (Multiconfiguration Aerosol TRacker of mIXing state): an aerosol microphysical module for global atmospheric models, Atmos. Chem. Phys, 8, 6003–6035, 10.5194/acp-8-6003-2008, 2008.

[R6] Bian H, Chin M, Hauglustaine DA, Schulz M, Myhre G, Bauer SE, Lund MT, Karydis VA, Kucsera TL, Pan X, Pozzer A, Skeie RB, Steenrod SD, Sudo K, Tsigaridis K, Tsimpidi AP, and Tsyro SG: Investigation of global particulate nitrate from the AeroCom phase III experiment, Atmos. Chem. Phys, 17, 12911–12940, 10.5194/acp-17-12911-2017, 2017.

[R7] Boucher O, Randall D, Artaxo P, Bretherton C, Feingold G, Forster P, Kerminen V-M, Kondo Y, Liao H, Lohmann U, Rasch P, Satheesh SK, Sherwood S, Stevens B, and Zhang XY: Clouds and Aerosols, in: Climate Change 2013 – The Physical Science Basis, edited by: Intergovernmental Panel on Climate Change, 571–658, Cambridge University Press, Cambridge, 2013.

[R8] Bougiatioti A, Fountoukis C, Kalivitis N, Pandis SN, Nenes A, and Mihalopoulos N: Cloud condensation nuclei measurements in the marine boundary layer of the Eastern Mediterranean: CCN closure and droplet growth kinetics, Atmos. Chem. Phys, 9, 7053–7066, 10.5194/acp-9-7053-2009, 2009.

[R9] Bougiatioti A, Nenes A, Fountoukis C, Kalivitis N, Pandis SN, and Mihalopoulos N: Size-resolved CCN distributions and activation kinetics of aged continental and marine aerosol, Atmos. Chem. Phys, 11, 8791–8808, 10.5194/acp-11-8791-2011, 2011.

[R10] Bougiatioti A, Bezantakos S, Stavroulas I, Kalivitis N, Kokkalis P, Biskos G, Mihalopoulos N, Papayannis A, and Nenes A: Biomass-burning impact on CCN number, hygroscopicity and cloud formation during summertime in the eastern Mediterranean, Atmos. Chem. Phys, 16, 7389–7409, 10.5194/acp-16-7389-2016, 2016.

[R11] Charlson RJ, Seinfeld JH, Nenes A, Kulmala M, Laaksonen A, and Facchini MC: Reshaping the Theory of Cloud Formation, Science, 292, 2025–2026, 2001. 11408648 10.1126/science.1060096

[R12] Cubison MJ, Ervens B, Feingold G, Docherty KS, Ulbrich IM, Shields L, Prather K, Hering S, and Jimenez JL: The influence of chemical composition and mixing state of Los Angeles urban aerosol on CCN number and cloud properties, Atmos. Chem. Phys, 8, 5649–5667, 10.5194/acp-8-5649-2008, 2008.

[R13] D’Andrea SD, Häkkinen SAK, Westervelt DM, Kuang C, Levin EJT, Kanawade VP, Leaitch WR, Spracklen DV, Riipinen I, and Pierce JR: Understanding global secondary organic aerosol amount and size-resolved condensational behavior, Atmos. Chem. Phys, 13, 11519–11534, 10.5194/acp-13-11519-2013, 2013.

[R14] D’Andrea SD, Ng JY, Kodros JK, Atwood SA, Wheeler MJ, Macdonald AM, Leaitch WR, and Pierce JR: Source attribution of aerosol size distributions and model evaluation using Whistler Mountain measurements and GEOS-Chem-TOMAS simulations, Atmos. Chem. Phys, 16, 383–396, 10.5194/acp-16-383-2016, 2016.

[R15] Daskalakis N, Myriokefalitakis S, and Kanakidou M: Sensitivity of tropospheric loads and lifetimes of short lived pollutants to fire emissions, Atmos. Chem. Phys, 15, 3543–3563, 10.5194/acp-15-3543-2015, 2015.

[R16] Deng ZZ, Zhao CS, Ma N, Ran L, Zhou GQ, Lu DR, and Zhou XJ: An examination of parameterizations for the CCN number concentration based on in situ measurements of aerosol activation properties in the North China Plain, Atmos. Chem. Phys, 13, 6227–6237, 10.5194/acp-13-6227-2013, 2013.

[R17] Dentener F, Kinne S, Bond T, Boucher O, Cofala J, Generoso S, Ginoux P, Gong S, Hoelzemann JJ, Ito A, Marelli L, Penner JE, Putaud J-P, Textor C, Schulz M, van der Werf GR, and Wilson J: Emissions of primary aerosol and precursor gases in the years 2000 and 1750 prescribed data-sets for AeroCom, Atmos. Chem. Phys, 6, 4321–4344, 10.5194/acp-6-4321-2006, 2006.

[R18] Dusek U, Frank GP, Hildebrandt L, Curtius J, Schneider J, Walter S, Chand D, Drewnick F, Hings S, Jung D, Borrmann S, and Andreae MO: Size Matters More Than Chemistry Aerosol Particles, Science, 80, 1375–1378, 10.1126/science.1125261, 2006. 16741120

[R19] Ervens B, Cubison M, Andrews E, Feingold G, Ogren JA, Jimenez JL, DeCarlo P, and Nenes A: Prediction of cloud condensation nucleus number concentration using measurements of aerosol size distributions and composition and light scattering enhancement due to humidity, J. Geophys. Res, 112, D10S32, 10.1029/2006JD007426, 2007.

[R20] Ervens B, Cubison MJ, Andrews E, Feingold G, Ogren JA, Jimenez JL, Quinn PK, Bates TS, Wang J, Zhang Q, Coe H, Flynn M, and Allan JD: CCN predictions using simplified assumptions of organic aerosol composition and mixing state: a synthesis from six different locations, Atmos. Chem. Phys, 10, 4795–4807, 10.5194/acp-10-4795-2010, 2010.

[R21] Fan J, Wang Y, Rosenfeld D, Liu X, Fan J, Wang Y, Rosenfeld D, and Liu X: Review of Aerosol – Cloud Interactions: Mechanisms, Significance, and Challenges, J. Atmos. Sci, 73, 4221–4252, 10.1175/JAS-D-16-0037.1, 2016.

[R22] Fanourgakis G, Kanakidou M, Nenes A, Bauer SE, Bergman T, Carslaw KS, Grini A, Hamilton DS, Johnson JS, Karydis VA, Kirkevåg A, Kodros JK, Lohmann U, Luo G, Makkonen R, Matsui H, Neubauer D, Pierce JR, Schmale J, Stier P, Tsigaridis K, van Noije T, Wang H, Watson-Parris D, Westervelt DM, Yang Y, Yoshioka M, Daskalakis N, Decesari S, Gysel-Beer M, Kalivitis N, Liu X, Mahowald NM, Myriokefalitakis S, Schrödner R, Sfakianaki M, Tsimpidi AP, Wu M, and Yu F: Data for the “Evaluation of global simulations of aerosol particle and cloud condensation nuclei number, with implications for cloud droplet formation”, Zenodo, Version v1, Data set, 10.5281/zenodo.3265866, 2019. PMC7709872 33273898

[R23] Feingold G: Analysis of smoke impact on clouds in Brazilian biomass burning regions: An extension of Twomey’s approach, J. Geophys. Res, 106, 22907–22922, 2001.

[R24] Feingold G and Siebert H: Cloud–Aerosol Interactions from the Micro to the Cloud Scale, in: Clouds in the Perturbed Climate System: Their Relationship to Energy Balance, Atmospheric Dynamics, and Precipitation, edited by: Heintzenberg J and Charlson RJ, MIT Press, 2009.

[R25] Fountoukis C and Nenes A: Continued development of a cloud droplet formation parameterization for global climate models, J. Geophys. Res.-Atmos, 110, 1–10, 10.1029/2004JD005591, 2005.

[R26] Fountoukis C and Nenes A: ISORROPIA II: a computationally efficient thermodynamic equilibrium model for $K^{+}-{\text{Ca}}^{2+}-{\text{Mg}}^{2+}-{\text{NH}}_{4}^{+}-{\text{Na}}^{+}$ $-{\text{SO}}_{4}^{2-}-{\text{NO}}_{3}^{-}-{\text{Cl}}^{-}-H_{2}O$ aerosols, Atmos. Chem. Phys, 7, 4639–4659, 10.5194/acp-7-4639-2007, 2007.

[R27] Ghan SJ, Gutzman G and Abdul-Razzak H: Competition between Sea Salt and Sulfate Particles as Cloud Condensation Nuclei, J. Atmos. Sci, 55, 3340–3347, 1998.

[R28] Gordon H, Kirkby J, Baltensperger U, Bianchi F, Breitenlechner M, Curtius J, Dias A, Dommen J, Donahue NM, Dunne EM, Duplissy J, Ehrhart S, Flagan RC, Frege C, Fuchs C, Hansel A, Hoyle CR, Kulmala M, Kürten A, Lehtipalo K, Makhmutov V, Molteni U, Rissanen MP, Stozkhov Y, Tröstl J, Tsagkogeorgas G, Wagner R, Williamson C, Wimmer D, Winkler PM, Yan C, and Carslaw KS: Causes and importance of new particle formation in the present-day and preindustrial atmospheres, J. Geophys. Res.-Atmos, 122, 8739–8760, 10.1002/2017JD026844, 2017.

[R29] Heald CL, Coe H, Jimenez JL, Weber RJ, Bahreini R, Middlebrook AM, Russell LM, Jolleys M, Fu T-M, Allan JD, Bower KN, Capes G, Crosier J, Morgan WT, Robinson NH, Williams PI, Cubison MJ, DeCarlo PF, and Dunlea EJ: Exploring the vertical profile of atmospheric organic aerosol: comparing 17 aircraft field campaigns with a global model, Atmos. Chem. Phys, 11, 12673–12696, 10.5194/acp-11-12673-2011, 2011.

[R30] Herrmann E, Weingartner E, Henne SL,V, Bukowiecki N, Steinbacher M, Conen F, Collaud Coen M, Hammer E, Jurányi Z, Baltensperger U, and Gysel M: Analysis of long-term aerosol size distribution data from Jungfraujoch with emphasis on free tropospheric conditions, cloud influence, and air mass transport. J. Geophys. Res, 120, 9459–9480, 10.1002/2015JD023660, 2015.

[R31] Huneeus N, Schulz M, Balkanski Y, Griesfeller J, Prospero J, Kinne S, Bauer S, Boucher O, Chin M, Dentener F, Diehl T, Easter R, Fillmore D, Ghan S, Ginoux P, Grini A, Horowitz L, Koch D, Krol MC, Landing W, Liu X, Mahowald N, Miller R, Morcrette J-J, Myhre G, Penner J, Perlwitz J, Stier P, Takemura T, and Zender CS: Global dust model intercomparison in AeroCom phase I, Atmos. Chem. Phys, 11, 7781–7816, 10.5194/acp-11-7781-2011, 2011.

[R32] Iwamoto Y, Kinouchi K, Watanabe K, Yamazaki N, and Matsuki A: Simultaneous measurement of CCN activity and chemical composition of fine-mode aerosols at Noto peninsula, Japan, in autumn 2012, Aerosol Air Qual. Res, 16, 2107–2118, 2016.

[R33] Jian Y and Fu T-M: Injection heights of springtime biomass-burning plumes over peninsular Southeast Asia and their impacts on long-range pollutant transport, Atmos. Chem. Phys, 14, 3977–3989, 10.5194/acp-14-3977-2014, 2014.

[R34] Jimenez JL, Canagaratna MR, Donahue NM, Prevot ASH, Zhang Q, Kroll JH, DeCarlo PF, Allan JD, Coe H, Ng NL, Aiken AC, Docherty KS, Ulbrich IM, Grieshop AP, Robinson AL, Duplissy J, Smith JD, Wilson KR, Lanz VA, Hueglin C, Sun YL, Tian J, Laaksonen A, Raatikainen T, Rautiainen J, Vaattovaara P, Ehn M, Kulmala M, Tomlinson JM, Collins DR, Cubison MJ, Dunlea EJ, Huffman JA, Onasch TB, Alfarra MR, Williams PI, Bower K, Kondo Y, Schneider J, Drewnick F, Borrmann S, Weimer S, Demerjian K, Salcedo D, Cottrell L, Griffin R, Takami A, Miyoshi T, Hatakeyama S, Shimono A, Sun JY, Zhang YM, Dzepina K, Kimmel JR, Sueper D, Jayne JT, Herndon SC, Trimborn AM, Williams LR, Wood EC, Middlebrook AM, Kolb CE, Baltensperger U, and Worsnop DR: Evolution of organic aerosols in the atmosphere, Science, 80, 1525–1529, 10.1126/science.1180353, 2009. 20007897

[R35] Johnson JS, Regayre LA, Yoshioka M, Pringle KJ, Lee LA, Sexton DMH, Rostron JW, Booth BBB, and Carslaw KS: The importance of comprehensive parameter sampling and multiple observations for robust constraint of aerosol radiative forcing, Atmos. Chem. Phys, 18, 13031–13053, 10.5194/acp-18-13031-2018, 2018.

[R36] Jurányi Z, Gysel M, Weingartner E, Bukowiecki N, Kammermann L, and Baltensperger U: A 17 month climatology of the cloud condensation nuclei number concentration at the high alpine site Jungfraujoch, J. Geophys. Res, 116, D10204, 10.1029/2010JD015199, 2011.

[R37] Kalivitis N, Kerminen V-M, Kouvarakis G, Stavroulas I, Bougiatioti A, Nenes A, Manninen HE, Petäjä T, Kulmala M, and Mihalopoulos N: Atmospheric new particle formation as a source of CCN in the eastern Mediterranean marine boundary layer, Atmos. Chem. Phys, 15, 9203–9215, 10.5194/acp-15-9203-2015, 2015.

[R38] Kalkavouras P, Bougiatioti A, Kalivitis N, Stavroulas I, Tombrou M, Nenes A, and Mihalopoulos N: Regional new particle formation as modulators of cloud condensation nuclei and cloud droplet number in the eastern Mediterranean, Atmos. Chem. Phys, 19, 6185–6203, 10.5194/acp-19-6185-2019, 2019.

[R39] Kanakidou M, Seinfeld JH, Pandis SN, Barnes I, Dentener FJ, Facchini MC, Van Dingenen R, Ervens B, Nenes A, Nielsen CJ, Swietlicki E, Putaud JP, Balkanski Y, Fuzzi S, Horth J, Moortgat GK, Winterhalter R, Myhre CEL, Tsigaridis K, Vignati E, Stephanou EG, and Wilson J: Organic aerosol and global climate modelling: a review, Atmos. Chem. Phys, 5, 1053–1123, 10.5194/acp-5-1053-2005, 2005.

[R40] Karydis VA, Capps SL, Russell AG, and Nenes A: Adjoint sensitivity of global cloud droplet number to aerosol and dynamical parameters, Atmos. Chem. Phys, 12, 9041–9055, 10.5194/acp-12-9041-2012, 2012.

[R41] Kerminen V-M, Petäjä T, Manninen HE, Paasonen P, Nieminen T, Sipilä M, Junninen H, Ehn M, Gagné S, Laakso L, Riipinen I, Vehkamäki H, Kurten T, Ortega IK, Dal Maso M, Brus D, Hyvärinen A, Lihavainen H, Leppä J, Lehtinen KEJ, Mirme A, Mirme S, Hõrrak U, Berndt T, Stratmann F, Birmili W, Wiedensohler A, Metzger A, Dommen J, Baltensperger U, Kiendler-Scharr A, Mentel TF, Wildt J, Winkler PM, Wagner PE, Petzold A, Minikin A, Plass-Dülmer C, Pöschl U, Laaksonen A, and Kulmala M: Atmospheric nucleation: highlights of the EUCAARI project and future directions, Atmos. Chem. Phys, 10, 10829–10848, 10.5194/acp-10-10829-2010, 2010.

[R42] Kerminen V-M, Paramonov M, Anttila T, Riipinen I, Fountoukis C, Korhonen H, Asmi E, Laakso L, Lihavainen H, Swietlicki E, Svenningsson B, Asmi A, Pandis SN, Kulmala M, and Petäjä T: Cloud condensation nuclei production associated with atmospheric nucleation: a synthesis based on existing literature and new results, Atmos. Chem. Phys, 12, 12037–12059, 10.5194/acp-12-12037-2012, 2012.

[R43] Kim D, Chin M, Yu H, Diehl T, Tan Q, Kahn RA, Tsigaridis K, Bauer SE, Takemura T, Pozzoli L, Bellouin N, Schulz M, Peyridieu S, Chédin A, and Koffi B: Sources, sinks, and transatlantic transport of North African dust aerosol: A multimodel analysis and comparison with remote sensing data, J. Geophys. Res.-Atmos, 119, 6259–6277, 10.1002/2013JD021099. 2014.

[R44] Kirkevåg A, Grini A, Olivié D, Seland Ø, Alterskjær K, Hummel M, Karset IHH, Lewinschal A, Liu X, Makkonen R, Bethke I, Griesfeller J, Schulz M, and Iversen T: A production-tagged aerosol module for Earth system models, OsloAero5.3 – extensions and updates for CAM5.3-Oslo, Geosci. Model Dev, 11, 3945–3982, 10.5194/gmd-11-3945-2018, 2018.

[R45] Köhler H: The nucleus in and the growth of hygroscopic droplets, Trans. Faraday Soc, 32, 1152–1161, 10.1039/TF9363201152, 1936.

[R46] Kristiansen NI, Stohl A, Olivié DJL, Croft B, Søvde OA, Klein H, Christoudias T, Kunkel D, Leadbetter SJ, Lee YH, Zhang K, Tsigaridis K, Bergman T, Evangeliou N, Wang H, Ma P-L, Easter RC, Rasch PJ, Liu X, Pitari G, Di Genova G, Zhao SY, Balkanski Y, Bauer SE, Faluvegi GS, Kokkola H, Martin RV, Pierce JR, Schulz M, Shindell D, Tost H, and Zhang H: Evaluation of observed and modelled aerosol lifetimes using radioactive tracers of opportunity and an ensemble of 19 global models, Atmos. Chem. Phys, 16, 3525–3561, 10.5194/acp-16-3525-2016, 2016.

[R47] Kulmala M and Kerminen VM: On the formation and growth of atmospheric nanoparticles, Atmos. Res, 90, 132–150, 10.1016/j.atmosres.2008.01.005, 2008.

[R48] Kulmala M, Laaksonen A, and Pirjola L: Parameterizations for sulfuric acid/water nucleation rates, J. Geophys. Res, 103, 8301, 10.1029/97JD03718, 1998.

[R49] Laaksonen A, Kulmala M, O’Dowd CD, Joutsensaari J, Vaattovaara P, Mikkonen S, Lehtinen KEJ, Sogacheva L, Dal Maso M, Aalto P, Petäjä T, Sogachev A, Yoon YJ, Lihavainen H, Nilsson D, Facchini MC, Cavalli F, Fuzzi S, Hoffmann T, Arnold F, Hanke M, Sellegri K, Umann B, Junkermann W, Coe H, Allan JD, Alfarra MR, Worsnop DR, Riekkola M-L, Hyötyläinen T, and Viisanen Y: The role of VOC oxidation products in continental new particle formation, Atmos. Chem. Phys, 8, 2657–2665, 10.5194/acp-8-2657-2008, 2008.

[R50] Lee LA, Pringle KJ, Reddington CL, Mann GW, Stier P, Spracklen DV, Pierce JR, and Carslaw KS: The magnitude and causes of uncertainty in global model simulations of cloud condensation nuclei, Atmos. Chem. Phys, 13, 8879–8914, 10.5194/acp-13-8879-2013, 2013.

[R51] Lee YH, Adams PJ, and Shindell DT: Evaluation of the global aerosol microphysical ModelE2-TOMAS model against satellite and ground-based observations, Geosci. Model Dev, 8, 631–667, 10.5194/gmd-8-631-2015, 2015.

[R52] Liu X, Easter RC, Ghan SJ, Zaveri R, Rasch P, Shi X, Lamarque J-F, Gettelman A, Morrison H, Vitt F, Conley A, Park S, Neale R, Hannay C, Ekman AML, Hess P, Mahowald N, Collins W, Iacono MJ, Bretherton CS, Flanner MG, and Mitchell D: Toward a minimal representation of aerosols in climate models: description and evaluation in the Community Atmosphere Model CAM5, Geosci. Model Dev, 5, 709–739, 10.5194/gmd-5-709-2012, 2012.

[R53] Liu X, Ma P-L, Wang H, Tilmes S, Singh B, Easter RC, Ghan SJ, and Rasch PJ: Description and evaluation of a new four-mode version of the Modal Aerosol Module (MAM4) within version 5.3 of the Community Atmosphere Model, Geosci. Model Dev, 9, 505–522, 10.5194/gmd-9-505-2016, 2016.

[R54] Makkonen R, Asmi A, Korhonen H, Kokkola H, Järvenoja S, Räisänen P, Lehtinen KEJ, Laaksonen A, Kerminen V-M, Järvinen H, Lohmann U, Bennartz R, Feichter J, and Kulmala M: Sensitivity of aerosol concentrations and cloud properties to nucleation and secondary organic distribution in ECHAM5-HAM global circulation model, Atmos. Chem. Phys, 9, 1747–1766, 10.5194/acp-9-1747-2009, 2009.

[R55] Mann GW, Carslaw KS, Ridley DA, Spracklen DV, Pringle KJ, Merikanto J, Korhonen H, Schwarz JP, Lee LA, Manktelow PT, Woodhouse MT, Schmidt A, Breider TJ, Emmerson KM, Reddington CL, Chipperfield MP, and Pickering SJ: Intercomparison of modal and sectional aerosol microphysics representations within the same 3-D global chemical transport model, Atmos. Chem. Phys, 12, 4449–4476, 10.5194/acp-12-4449-2012, 2012.

[R56] Matsui H: Development of a global aerosol model using a two-dimensional sectional method?: 1, Model design, J. Adv. Model. Earth Syst, 9, 1921–1947, 10.1002/2017MS000936, 2017.

[R57] McFiggans G, Artaxo P, Baltensperger U, Coe H, Facchini MA, Feingold G, Fuzzi S, Gysel M, Laaksonen A, Lohmann U, Mentel TF, Murphy DM, O’Dowd CD, Snider JR, and Weingartner E: The effect of physical and chemical aerosol properties on warm cloud droplet activation, Atmos. Chem. Phys, 6, 2593–2649, 10.5194/acp-6-2593-2006, 2006.

[R58] Metzger S, Dentener F, Pandis S, and Lelieveld J: Gas/aerosol partitioning: 1. A computationally efficient model, J. Geophys. Res.-Atmos, 107, 16-1–24, 10.1029/2001JD001102, 2002a.

[R59] Metzger S, Dentener F, Krol M, Jeuken A, and Lelieveld J: Gas/aerosol partitioning 2. Global modeling results, J. Geophys. Res.-Atmos, 107, 1–23, 10.1029/2001JD001103, 2002b.

[R60] Moore RH, Karydis VA, Capps SL, Lathem TL, and Nenes A: Droplet number uncertainties associated with CCN: an assessment using observations and a global model adjoint, Atmos. Chem. Phys, 13, 4235–4251, 10.5194/acp-13-4235-2013, 2013.

[R61] Morales Betancourt R and Nenes A: Characteristic updrafts for computing distribution-averaged cloud droplet number and stratocumulus cloud properties, J. Geophys. Res.-Atmos, 115, 2–9, 10.1029/2009JD013233, 2010.

[R62] Morales Betancourt R and Nenes A: Understanding the contributions of aerosol properties and parameterization discrepancies to droplet number variability in a global climate model, Atmos. Chem. Phys, 14, 4809–4826, 10.5194/acp-14-4809-2014, 2014a.

[R63] Morales Betancourt R and Nenes A: Droplet activation parameterization: the population-splitting concept revisited, Geosci. Model Dev, 7, 2345–2357, 10.5194/gmd-7-2345-2014, 2014b.

[R64] Myhre G, Samset BH, Schulz M, Balkanski Y, Bauer S, Berntsen TK, Bian H, Bellouin N, Chin M, Diehl T, Easter RC, Feichter J, Ghan SJ, Hauglustaine D, Iversen T, Kinne S, Kirkevåg A, Lamarque J-F, Lin G, Liu X, Lund MT, Luo G, Ma X, van Noije T, Penner JE, Rasch PJ, Ruiz A, Seland Ø, Skeie RB, Stier P, Takemura T, Tsigaridis K, Wang P, Wang Z, Xu L, Yu H, Yu F, Yoon J-H, Zhang K, Zhang H, and Zhou C: Radiative forcing of the direct aerosol effect from AeroCom Phase II simulations, Atmos. Chem. Phys, 13, 1853–1877, 10.5194/acp-13-1853-2013, 2013.

[R65] Myriokefalitakis S, Nenes A, Baker AR, Mihalopoulos N, and Kanakidou M: Bioavailable atmospheric phosphorous supply to the global ocean: a 3-D global modeling study, Biogeosciences, 13, 6519–6543, 10.5194/bg-13-6519-2016, 2016.

[R66] Nenes A and Seinfeld JH: Parameterization of cloud droplet formation in global climate models, J. Geophys. Res.-Atmos, 108, 4415, 10.1029/2002JD002911, 2003.

[R67] Ovadnevaite J, Ceburnis D, Leinert S, Dall’Osto M, Canagaratna M, O’Doherty S, Berresheim H, and O’Dowd C: Submicron NE Atlantic marine aerosol chemical composition and abundance: Seasonal trends and air mass categorization, J. Geophys. Res.-Atmos, 119, 11850–11863, 10.1002/2013JD021330, 2014.

[R68] Petters MD and Kreidenweis SM: A single parameter representation of hygroscopic growth and cloud condensation nucleus activity, Atmos. Chem. Phys, 7, 1961–1971, 10.5194/acp-7-1961-2007, 2007.

[R69] Pierce JR, Riipinen I, Kulmala M, Ehn M, Petäjä T, Junninen H, Worsnop DR, and Donahue NM: Quantification of the volatility of secondary organic compounds in ultrafine particles during nucleation events, Atmos. Chem. Phys, 11, 9019–9036, 10.5194/acp-11-9019-2011, 2011.

[R70] Pringle KJ, Tost H, Pozzer A, Pöschl U, and Lelieveld J: Global distribution of the effective aerosol hygroscopicity parameter for CCN activation, Atmos. Chem. Phys, 10, 5241–5255, 10.5194/acp-10-5241-2010, 2010.

[R71] Pruppacher HR and Klett JD: Microphysics of clouds and precipitation, 2nd ed., Kluwer Academic Publishers, Dordrecht, The Netherlands, 1997.

[R72] Reutter P, Su H, Trentmann J, Simmel M, Rose D, Gunthe SS, Wernli H, Andreae MO, and Pöschl U: Aerosol- and updraft-limited regimes of cloud droplet formation: influence of particle number, size and hygroscopicity on the activation of cloud condensation nuclei (CCN), Atmos. Chem. Phys, 9, 7067–7080, 10.5194/acp-9-7067-2009, 2009.

[R73] Riccobono F, Schobesberger S, Scott CE, Dommen J, Ortega IK, Rondo L, Almeida J, Amorim A, Bianchi F, Breitenlechner M, David A, Downard A, Dunne EM, Duplissy J, Ehrhart S, Flagan RC, Franchin A, Hansel A, Junninen H, Kajos M, Keskinen H, Kupc A, Kürten A, Kvashin AN, Laaksonen A, Lehtipalo K, Makhmutov V, Mathot S, Nieminen T, Onnela A, Petäjä T, Praplan AP, Santos FD, Schallhart S, Seinfeld JH, Sipilä M, Spracklen DV, Stozhkov Y, Stratmann F, Tomé A, Tsagkogeorgas G, Vaattovaara P, Viisanen Y, Vrtala A, Wagner PE, Weingartner E, Wex H, Wimmer D, Carslaw KS, Curtius J, Donahue NM, Kirkby J, Kulmala M, Worsnop DR, and Baltensperger U: Oxidation Products of Biogenic Emissions Contribute to Nucleation of Atmospheric Particles, Science, 80, 717–721, 2014. 10.1126/science.1243527 24833386

[R74] Rissman T, Nenes A, and Seinfeld JH: Chemical amplification (or dampening) of the Twomey effect: Conditions derived from droplet activation theory, J. Atmos. Sci, 61, 919–930, 2004.

[R75] Schmale J, Henning S, Henzing B, Keskinen H, Sellegri K, Ovadnevaite J, Bougiatioti A, Kalivitis N, Stavroulas I, Jefferson A, Park M, Schlag P, Kristensson A, Iwamoto Y, Pringle K, Reddington C, Aalto P, Äijälä M, Baltensperger U, Bialek J, Birmili W, Bukowiecki N, Ehn M, Fjæraa AM, Fiebig M, Frank G, Fröhlich R, Frumau A, Furuya M, Hammer E, Heikkinen L, Herrmann E, Holzinger R, Hyono H, Kanakidou M, Kiendler-Scharr A, Kinouchi K, Kos G, Kulmala M, Mihalopoulos N, Motos G, Nenes A, O’Dowd C, Paramonov M, Petäjä T, Picard D, Poulain L, Prévôt ASH, Slowik J, Sonntag A, Swietlicki E, Svenningsson B, Tsurumaru H, Wiedensohler A, Wittbom C, Ogren JA, Matsuki A, Yum SS, Myhre CL, Carslaw K, Stratmann F, and Gysel M: Collocated observations of cloud condensation nuclei, particle size distributions, and chemical composition, Sci. Data, 4, 170003, 10.1038/sdata.2017.3, 2017. 28291234 PMC5349251

[R76] Schmale J, Henning S, Decesari S, Henzing B, Keskinen H, Sellegri K, Ovadnevaite J, Pöhlker ML, Brito J, Bougiatioti A, Kristensson A, Kalivitis N, Stavroulas I, Carbone S, Jefferson A, Park M, Schlag P, Iwamoto Y, Aalto P, Äijälä M, Bukowiecki N, Ehn M, Frank G, Fröhlich R, Frumau A, Herrmann E, Herrmann H, Holzinger R, Kos G, Kulmala M, Mihalopoulos N, Nenes A, O’Dowd C, Petäjä T, Picard D, Pöhlker C, Pöschl U, Poulain L, Prévôt ASH, Swietlicki E, Andreae MO, Artaxo P, Wiedensohler A, Ogren J, Matsuki A, Yum SS, Stratmann F, Baltensperger U, and Gysel M: Long-term cloud condensation nuclei number concentration, particle number size distribution and chemical composition measurements at regionally representative observatories, Atmos. Chem. Phys, 18, 2853–2881, 10.5194/acp-18-2853-2018, 2018.

[R77] Schutgens NAJ, Gryspeerdt E, Weigum N, Tsyro S, Goto D, Schulz M, and Stier P: Will a perfect model agree with perfect observations? The impact of spatial sampling, Atmos. Chem. Phys, 16, 6335–6353, 10.5194/acp-16-6335-2016, 2016.

[R78] Seinfeld J and Pandis S: Atmospheric Chemistry and Physics: From Air Pollution to Climate Change, J. Wiley, New York, 2006.

[R79] Seinfeld JH, Bretherton C, Carslaw KS, Coe H, DeMott PJ, Dunlea EJ, Feingold G, Ghan S, Guenther AB, Kahn R, Kraucunas I, Kreidenweis SM, Molina MJ, Nenes A, Penner JE, Prather KA, Ramanathan V, Ramaswamy V, Rasch PJ, Ravishankara AR, Rosenfeld D, Stephens G, and Wood R: Improving our fundamental understanding of the role of aerosol–cloud interactions in the climate system, P. Natl. Acad. Sci. USA, 113, 5781–5790, 10.1073/pnas.1514043113, 2016. PMC4889348 27222566

[R80] Sotiropoulou R-EP, Medina J, and Nenes A: CCN predictions: Is theory sufficient for assessments of the indirect effect?, Geophys. Res. Lett, 33, L05816, 10.1029/2005GL025148, 2006.

[R81] Sotiropoulou REP, Nenes A, Adams PJ, and Seinfeld JH: Cloud condensation nuclei prediction error from application of Köhler theory: Importance for the aerosol indirect effect, J. Geophys. Res.-Atmos, 112, D12202, 10.1029/2006JD007834, 2007.

[R82] Spracklen DV, Bonn B, and Carslaw KS: Boreal forests, aerosols and the impacts on clouds and climate, Philos. Trans. A. Math. Phys. Eng. Sci, 366, 4613–26, 10.1098/rsta.2008.0201, 2008. 18826917

[R83] Spracklen DV, Carslaw KS, Pöschl U, Rap A, and Forster PM: Global cloud condensation nuclei influenced by carbonaceous combustion aerosol, Atmos. Chem. Phys, 11, 9067–9087, 10.5194/acp-11-9067-2011, 2011.

[R84] Spracklen DV, Carslaw KS, Merikanto J, Mann GW, Reddington CL, Pickering S, Ogren JA, Andrews E, Baltensperger U, Weingartner E, Boy M, Kulmala M, Laakso L, Lihavainen H, Kivekäs N, Komppula M, Mihalopoulos N, Kouvarakis G, Jennings SG, O’Dowd C, Birmili W, Wiedensohler A, Weller R, Gras J, Laj P, Sellegri K, Bonn A, Krejci R, Laaksonen A, Hamed A, Minikin A, Harrison RM, Talbot R, and Sun J: Explaining global surface aerosol number concentrations in terms of primary emissions and particle formation, Atmos. Chem. Phys, 10, 4775–4793, 10.5194/acp-10-4775-2010, 2010.

[R85] Sullivan RC, Moore MJK, Petters MD, Kreidenweis SM, Roberts GC, and Prather KA: Effect of chemical mixing state on the hygroscopicity and cloud nucleation properties of calcium mineral dust particles, Atmos. Chem. Phys, 9, 3303–3316, 10.5194/acp-9-3303-2009, 2009.

[R86] Sullivan SC, Lee D, Oreopoulos L, and Nenes A: Role of updraft velocity in temporal variability of global cloud hydrometeor number, P. Natl. Acad. Sci. USA, 113, 5791–5796, 10.1073/pnas.1514039113, 2016. PMC4889347 27185952

[R87] Tegen I, Neubauer D, Ferrachat S, Siegenthaler-Le Drian C, Bey I, Schutgens N, Stier P, Watson-Parris D, Stanelle T, Schmidt H, Rast S, Kokkola H, Schultz M, Schroeder S, Daskalakis N, Barthel S, Heinold B, and Lohmann U: The global aerosol–climate model ECHAM6.3–HAM2.3 – Part 1: Aerosol evaluation, Geosci. Model Dev, 12, 1643–1677, 10.5194/gmd-12-1643-2019, 2019.

[R88] Textor C, Schulz M, Guibert S, Kinne S, Balkanski Y, Bauer S, Berntsen T, Berglen T, Boucher O, Chin M, Dentener F, Diehl T, Easter R, Feichter H, Fillmore D, Ghan S, Ginoux P, Gong S, Grini A, Hendricks J, Horowitz L, Huang P, Isaksen I, Iversen I, Kloster S, Koch D, Kirkevåg A, Kristjansson JE, Krol M, Lauer A, Lamarque JF, Liu X, Montanaro V, Myhre G, Penner J, Pitari G, Reddy S, Seland Ø, Stier P, Takemura T, and Tie X: Analysis and quantification of the diversities of aerosol life cycles within AeroCom, Atmos. Chem. Phys, 6, 1777–1813, 10.5194/acp-6-1777-2006, 2006.

[R89] Tröstl J, Chuang WK, Gordon H, Heinritzi M, Yan C, Molteni U, Ahlm L, Frege C, Bianchi F, Wagner R, Simon M, Lehtipalo K, Williamson C, Craven JS, Duplissy J, Adamov A, Almeida J, Bernhammer AK, Breitenlechner M, Brilke S, Dias A, Ehrhart S, Flagan RC, Franchin A, Fuchs C, Guida R, Gysel M, Hansel A, Hoyle CR, Jokinen T, Junninen H, Kangasluoma J, Keskinen H, Kim J, Krapf M, Kürten A, Laaksonen A, Lawler M, Leiminger M, Mathot S, Möhler O, Nieminen T, Onnela A, Petäjä T, Piel FM, Miettinen P, Rissanen MP, Rondo L, Sarnela N, Schobesberger S, Sengupta K, Sipilä M, Smith JN, Steiner G, Tomè A, Virtanen A, Wagner AC, Weingartner E, Wimmer D, Winkler PM, Ye P, Carslaw KS, Curtius J, Dommen J, Kirkby J, Kulmala M, Riipinen I, Worsnop DR, Donahue NM, and Baltensperger U: The role of low-volatility organic compounds in initial particle growth in the atmosphere, Nature, 533, 527–531, 10.1038/nature18271, 2016. 27225126 PMC8384036

[R90] Tsigaridis K, Koch D, and Menon S: Uncertainties and importance of sea spray composition on aerosol direct and indirect effects, J. Geophys. Res.-Atmos, 118, 220–235, 10.1029/2012JD018165, 2013.

[R91] Tsigaridis K, Daskalakis N, Kanakidou M, Adams PJ, Artaxo P, Bahadur R, Balkanski Y, Bauer SE, Bellouin N, Benedetti A, Bergman T, Berntsen TK, Beukes JP, Bian H, Carslaw KS, Chin M, Curci G, Diehl T, Easter RC, Ghan SJ, Gong SL, Hodzic A, Hoyle CR, Iversen T, Jathar S, Jimenez JL, Kaiser JW, Kirkevåg A, Koch D, Kokkola H, Lee YH, Lin G, Liu X, Luo G, Ma X, Mann GW, Mihalopoulos N, Morcrette J-J, Müller J-F, Myhre G, Myriokefalitakis S, Ng NL, O’Donnell D, Penner JE, Pozzoli L, Pringle KJ, Russell LM, Schulz M, Sciare J, Seland Ø, Shindell DT, Sillman S, Skeie RB, Spracklen D, Stavrakou T, Steenrod SD, Takemura T, Tiitta P, Tilmes S, Tost H, van Noije T, van Zyl PG, von Salzen K, Yu F, Wang Z, Wang Z, Zaveri RA, Zhang H, Zhang K, Zhang Q, and Zhang X: The AeroCom evaluation and intercomparison of organic aerosol in global models, Atmos. Chem. Phys, 14, 10845–10895, 10.5194/acp-14-10845-2014, 2014.

[R92] Twomey S: The nuclei of natural cloud formation. II The supersaturation in natural clouds and the variation of cloud droplet concentration, Geofis. Pura Appl, 43, 243–249, 1959.

[R93] Twomey S: The Influence of Pollution on the Shortwave Albedo of Clouds, J. Atmos. Sci, 34, 1149–1152, 10.1175/1520-0469(1977)034<1149:TIOPOT>2.0.CO;2, 1977.

[R94] Vehkamäki H: An improved parameterization for sulfuric acid–water nucleation rates for tropospheric and stratospheric conditions, J. Geophys. Res, 107, 4622, 10.1029/2002JD002184, 2002.

[R95] Vignati E, Wilson J, and Stier P: M7: An efficient size-resolved aerosol microphysics module for large-scale aerosol transport models, J. Geophys. Res.-Atmos, 109, D22202, 10.1029/2003JD004485, 2004.

[R96] Wang H, Easter RC, Rasch PJ, Wang M, Liu X, Ghan SJ, Qian Y, Yoon J-H, Ma P-L, and Vinoj V: Sensitivity of remote aerosol distributions to representation of cloud–aerosol interactions in a global climate model, Geosci. Model Dev, 6, 765–782, 10.5194/gmd-6-765-2013, 2013.

[R97] Yoshioka M, Regayre LA, Pringle KJ, Johnson JS, Mann GW, Partridge DG, Sexton DMH, Lister GMS, Schutgens N, Stier P, Kipling Z, Bellouin N, Browse J, Booth BBB, Johnson CE, Johnson B, Mollard JDP, Lee L, and Carslaw KS: Ensembles of Global Climate Model Variants Designed for the Quantification and Constraint of Uncertainty in Aerosols and their Radiative Forcing, J. Adv. Model. Earth Syst., under review, 2019.

[R98] Yu F: A secondary organic aerosol formation model considering successive oxidation aging and kinetic condensation of organic compounds: global scale implications, Atmos. Chem. Phys, 11, 1083–1099, 10.5194/acp-11-1083-2011, 2011.

[R99] Yu F and Luo G: Simulation of particle size distribution with a global aerosol model: contribution of nucleation to aerosol and CCN number concentrations, Atmos. Chem. Phys, 9, 7691–7710, 10.5194/acp-9-7691-2009, 2009.

[R100] Yu F, Nadykto AB, Herb J, Luo G, Nazarenko KM, and Uvarova LA: H_2_SO_4_─H_2_O─NH_3_ ternary ion-mediated nucleation (TIMN): kinetic-based model and comparison with CLOUD measurements, Atmos. Chem. Phys, 18, 17451–17474, 10.5194/acp-18-17451-2018, 2018.

