# Supplementary material for: Evaluation of global simulations of aerosol particle and cloud condensation nuclei number, with implications for cloud droplet formation
Source: Atmos Chem Phys. Author manuscript; Available in PMC 2020 Dec 2. (PMC7709872; doi:10.5194/acp-19-8591-2019)
Supplement: Supplementary text [file NIHMS1643828-supplement-Supplementary_text.pdf]

Supplement of Atmos. Chem. Phys., 19, 8591–8617, 2019
https://doi.org/10.5194/acp-19-8591-2019-supplement
© Author(s) 2019. This work is distributed under
the Creative Commons Attribution 4.0 License.

Supplement of

Evaluation of global simulations of aerosol particle and cloud condensation nuclei number, with implications for cloud droplet formation

George S. Fanourgakis et al.

Correspondence to: Maria Kanakidou (mariak@uoc.gr) and Athanasios Nenes (athanasios.nenes@epfl.ch)

The copyright of individual parts of the supplement might differ from the CC BY 4.0 License.

Table of Contents

Appendix S1. Models and measurements description 2
Table S1. Host model description 6
Table S2. Details about the microphysics schemes employed by the models. 7
Table S3. Emission inventories and schemes used in the models. 9
Table S4. Biomass burning emission's injection height and dry and wet deposition parameterization in the models. 12
Table S5. Measurement sites included in this study. 17
Appendix S2 18
S2.1.Particle numbers, CCN calculations 18
S2.2. Persistence – autocorrelation function 18
Appendix S3. Results 19
S3.1 CCN persistence- Individual model's behavior and sensitivity to size of the emitted particles 19
S3.2. Statistics for the comparisons between model results and observations 21
S3.3. Surface PM1 composition - Global distributions 21
References 23
Supplementary figures captions 31

Appendix S1. Models and measurements description

Details on aerosol microphysics representation in the participating models (Tables S1, S2, S3):

The original M7 scheme (Vignati et al., 2004) uses seven log-normal distributions to represent aerosol populations. Four of them are describing mixed (water-soluble) particles, in the nucleation, Aitken, accumulation and coarse modes, while the remaining three modes include insoluble particles in the Aitken, accumulation and coarse modes. Each mode is represented by the total number concentration of particles and the mass mixing ratio of each aerosol component. Soluble particles are formed from insoluble ones by coagulation and condensation.

With respect to the original M7 scheme, ECHAM5.5-HAM2-ELVOC_UH and TM4-ECPL are using an improved numerical scheme (Kokkola et al., 2009) to compute the formation of sulfuric acid by oxidation of SO_2, and its removal by nucleation and condensation on pre-existing particles. The EMAC model uses the Global Modal-aerosol eXtension (GMXe) microphysics model (Pringle et al., 2010a), which is also based on M7 and allows the treatment of additional aerosol species (sodium, chloride, magnesium, calcium, and potassium).

The ECHAM5.5-HAM2-ELVOC_UH, ECHAM6-HAM2-AP and ECHAM6-HAM2 models are using the same aerosol module HAM2 (Zhang et al., 2012; Tegen et al, 2018). Secondary organic aerosols (SOA) produced by oxidation of isoprene, monoterpene, and anthropogenic precursors and primary organic aerosols (POA) are treated as different tracers within the HAM2-ELVOC-UH module, while in ECHAM6-HAM2 and ECHAM6-HAM2-AP SOA is added to POA (see Tables S2 and S3). ECHAM6-HAM2-AP is identical to ECHAM6-HAM2 with the difference that the aerosol processing (AP) scheme in stratiform clouds by Neubauer et al. (2014) is used, which is adapted from Hoose et al. (2008a, 2008b). In addition to the seven modes in ECHAM6-HAM2, ECHAM6-HAM2-AP has an explicit representation of aerosol particles in cloud droplets and ice crystals in stratiform clouds, which are represented by 5 tracers (sulphate – SO4, black carbon - BC, organic aerosol - OA, dust- DU and sea-salt- SS). Nucleation and impaction scavenging of aerosols, freezing and evaporation of cloud droplets, and melting and sublimation of ice crystals are treated explicitly. Aerosol particles from evaporating precipitation are released to modes, which correspond to their size and can therefore act as CCN or INP again.

The TM4-ECPL model also treats POA and SOA separately. The latter are produced from semi-volatile organics formed by oxidation of isoprene, terpene (a- and b- pinenes) and aromatic (benzene, toluene and xylene) compounds (Tsigaridis and Kanakidou, 2003; Tsigaridis and Kanakidou, 2007) as well as by aqueous phase chemistry (Myriokefalitakis et al., 2011). For this study, although TM4-ECPL uses ISORROPIA II thermodynamic model (Fountoukis and Nenes, 2007) to calculate the partitioning between HNO_3 and NO_3^-, the contribution of particulate NO_3^- to aerosol formation and growth is not taken into account.

TM5 uses a similar host model as TM4-ECPL. TM5 considers SOA formation from isoprene and monoterpenes. SOA formation mechanism is implemented according to Jokinen et al. (2015). Both monoterpenes and isoprene produce extremely low volatility organic compounds (ELVOCs) and semi-volatile products with prescribed yields (from Jokinen et al., 2015). Oxidation by OH and O_3 is considered. The ELVOCs participate in aerosol nucleation, which is parameterized

according to Riccobono et al. (2014) accounting for sulfuric acid and organic vapors. In addition, ELVOCs in TM5 provide early growth for nucleation mode particles, as they are distributed to particle phase according to condensation sink. The semi-volatile products are distributed to particle phase according to particle-phase organic mass, as in Jokinen et al. (2015). Hence, after oxidation, no SOA products remain in the gas-phase, but immediate condensation to aerosol-phase is assumed. The approach provides dynamics for aerosol mass and number formation, but does not consider e.g. particle-phase reactions, evaporation of organic material, or aqueous-phase SOA formation. More detailed description can be found in Bergman et al. (in prep.)

CAM5-MAM3 employs the three-mode modal aerosol module MAM3 (Liu et al., 2012). Within each of the three modes (Aitken, accumulation, and coarse mode) all types of aerosols are internally mixed, while particles between different modes are externally mixed. SO4, BC, POA, SOA, SS, DU are the aerosol components considered using a total of 15 aerosol tracers. CAM5-MAM4 has an extra primary-carbon mode to treat freshly emitted POA and BC, amounting to in total 18 aerosol tracers (Liu et al., 2016). The MAM3 and MAM4 aerosol modules describe the following microphysical processes: nucleation, condensation, coagulation, ageing, dry (gravitational and turbulent) deposition, wet (in-cloud and below-cloud scavenging) deposition, in-cloud activation, and release from the evaporation of clouds and raindrops.

Two of the global models (GEOS-Chem-APM and CAM5-Chem-APM) are using the sectional Advanced Particle Microphysics (APM) package (Yu and Luo, 2009). The APM package is optimized to accurately simulate secondary particles (composed of SO4, NO3, NH4, and SOA) formation and their growth to CCN sizes, with a higher size resolution for the size range of importance (1.2–120 nm: 30 bins, 10 additional bins for 120 nm–12 μm). 20 sectional bins represent sea salt, covering dry diameters from 0.012 to 12 μm, and 15 sectional bins represent dust for 0.03–50 μm. Two modes (Aitken mode and accumulation mode) are used in this study to represent hydrophobic and hydrophilic BC and POC in the model. APM also considers the aging of BC and OA that turns the hydrophobic BC and OA to hydrophilic. Coating of SO4 and other secondary aerosols on primary particles such as BC, OA, SS, and DU are considered. The contribution of nitrate, ammonium, and SOA to SO4 particle growth are considered. Via the coating process caused by coagulation, condensation, and in-cloud oxidation, some secondary species attach to primary particles and are transported and scavenged with these primary particles. The kinetic condensation of low volatile secondary organic gas (LV-SOG) in addition to sulfuric acid on nucleated particles is calculated based on a scheme that considers the SOG volatility changes arising from the oxidation aging (Yu, 2011).

TwO-Moment Aerosol Sectional (TOMAS) microphysics package (Adams and Seinfeld, 2002; Lee and Adams, 2012) was used by the GISS-E2-TOMAS (Lee et al., 2015) and GEOS-Chem-TOMAS (Kodros et al., 2016) global models. Both models simulate SO4, SS, OA, BC and DU. In GEOS-Chem-TOMAS, TOMAS tracks two independent moments (number and mass) within each of 15 size sections for size-resolved condensation, coagulation, nucleation, dry deposition, wet deposition, emissions, and aqueous chemistry. Condensation includes sulfuric acid and SOA (including both biogenic SOA and anthropogenically enhanced SOA as described in D'Andrea et al., 2013). GEOS-Chem-TOMAS accounts for binary (Vehkamaki et al., 2002) and ammonia ternary (Napari et al., 2002) nucleation, where ternary nucleation rates are scaled

down by a factor of 105 to match observed nucleation rates (Westervelt et al., 2013). TOMAS has also been integrated into GISS-E2 (Lee et al., 2015), a fully coupled Earth System Model with interactive atmosphere, land, ocean, and sea ice components (Schmidt et al., 2014). TOMAS in GISS-E2 consists of 15 size sections covering 3 nm to 10 μm. Condensation in GISS-E2-TOMAS includes both sulfuric acid and a simpler treatment of SOA than is present in GEOS-Chem-TOMAS, in which 10% of terpene emissions are condensed as hydrophilic organic aerosol, resulting in a production rate of 17.1 Tg a^{-1}. GISS-E2-TOMAS uses a physically-based aerosol-cloud activation parameterization from Nenes and Seinfeld (2003), using both modeled large-scale and sub-grid updraft velocity.

The Multiconfiguration Aerosol TRacker of mIXing state (MATRIX) module (Bauer et al., 2008; 2010; Bauer and Menon, 2012) is used by GISS-E2.1-MATRIX. MATRIX is an aerosol microphysics scheme based on the quadrature method of moments (QMOM; McGraw, 1997). MATRIX represents new particle formation (binary and ternary nucleation), particle emissions, gas-particle mass transfer, aerosol phase chemistry, condensational growth, and coagulation within and between particle populations. Condensational growth is calculated for the sulphate-ammonium-nitrate-water system. Unique to MATRIX is the ability to explicitly simulate aerosol mixing state (Bauer et al., 2013). For this study MATRIX is set up with 16 aerosol populations, as defined by their mixing state. The tracked variables vary by population but can include number concentration and mass concentration of SO4, NO3, NH4, aerosol water, BC, OA, mineral dust, and SS. MATRIX is coupled to the NASA GISS climate model, GISS-E2.1, an updated version of GISS-E2 model (Schmidt et al., 2014). Sea-salt, dust and isoprene emission fluxes are calculated interactively, and additional natural and anthropogenic fluxes are from the CMIP6 inventory (Hoesly et al., 2017; van Marle et al., 2017).

The CAM5-chem-ATRAS2 is using the Aerosol Two-dimensional bin module for foRmation and Aging Simulation version 2 (ATRAS2) (Matsui, 2017; Matsui and Mahowald, 2017). The ATRAS2 model uses 12 size bins from 0.001 to 10 μm in aerosol dry diameter. BC mixing state is resolved with 8, 3, or 1 bins for each size bin. In this study, we perform simulations with a single mixing state representation. Mass concentrations of eight aerosol species (SO4, NO3, NH4, DU, SS, OA (POA+SOA), BC, and water) and number concentrations are calculated for each bin in the model. The number of advected species (chemistry and aerosol) is 215 in this study. The CAM5-chem-ATRAS2 model considers emissions, gas-phase chemistry, condensation and evaporation of SO4, NO3, NH4, and OA, coagulation, nucleation, activation of aerosol and evaporation from cloud, aerosol formation in clouds, dry and wet deposition, aerosol optical properties, aerosol-radiation interactions, and aerosol-cloud interactions. OA formation is calculated by a volatility basis-set approach which considers oxidations of POA (gas-phase), alkanes, alkenes, aromatics, isoprene, and monoterpene (Matsui et al., 2014; Matsui, 2017). Nucleation is calculated by the activation-type theory (Kulmala et al., 2006) within the planetary boundary layer and by a binary homogeneous nucleation scheme (Vehkamäki et al., 2002) within the free troposphere.

CAM5.3-Oslo (Kirkevåg et al., 2018; Karset et al., 2018) includes a “production tagged” aerosol life-cycle module (OsloAero5.3), and comes with an offline microphysics scheme (AeroTab5.3), which is used to make look-up tables for aerosol optics and dry sizes. In AeroTab5.3, the size distributions of number and mass concentrations are estimated by solving the respective continuity equations, using 44 size bins with radii ranging from 0.001 to 20 μm. The size modes are

assumed to be log-normally distributed at the point of emission or nucleation, but are modified by condensation, coagulation, and cloud processing. The term “production-tagged” is used since the tracers that change the aerosol size distributions are tagged according to their production pathway (e.g., condensation). In the aerosol life-cycle module, scavenging and dry deposition also change the total size distribution, but not the shape/radius-dependence of each of the size modes. The size modes are here assumed to be log-normal, either with prescribed typical sizes, or with log-normal fits (through look-up tables, for use in the CCN activation) to the respective size distributions calculated offline. In total 21 aerosol tracers are taken into account, distributed onto 10 internally mixed and 2 externally mixed (except with water through hygroscopic growth) size-modes, consisting of SO4, SS, OC, BC, and DU.

HadGEM3-UKCA includes the GLOMAP modal aerosol scheme, which is a two-moment scheme representing seven (7) lognormal modes; soluble nucleation, Aitken, accumulation and coarse modes and insoluble Aitken, accumulation and coarse modes. Each of these modes carries particle numbers and masses for 5 aerosol components; SO4, BC, OC (including both primary and secondary aerosols), DU and SS. Insoluble mode particles can be aged and become soluble when soluble secondary aerosol materials condense on them and form coating. In the lowermost atmosphere particles are subject to dry deposition. All particles are also subject to impaction scavenging by rainfall, while soluble particles are in addition subject to nucleation scavenging in large scale and convective rainfall.

Table S1. Host model description

**Model**	**Horizontal resolution** (latitude × longitude)	**Vertical resolution**	**Host Model**	**Meteorology**	**Model references**
CAM5-Chem-APM	1.875° × 2.5°	32	GCM	Online, Nudged with MERRA2	(Luo and Yu, 2018 in prep.)
CAM5-Chem-ATRAS2	1.875° × 2.5°	30 up to 3.6 hPa	GCM (nudged)	MERRA U and V (6h)	(Matsui, 2017) (Matsui and Mahowald, 2017)
CAM5_MAM3	1.875° × 2.5°	30 up to 3.6 hPa	GCM (nudg)	MERRA U and V (6h)	(Neale et al., 2012) (Wang et al., 2013), (Yang et al., 2017)
CAM5_MAM4	1.875° × 2.5°	56 up to 40 Km	GCM (nudged)	MERRA	(Neale et al., 2012) (Wang et al., 2013), (Yang et al., 2017)
CAM5.3-Oslo	0.938° × 1.25°	30 up to 3.6 hPa	GCM (nudg)	ERA-interim U, V and PS (6h)	(Kirkevåg et al., 2018); (Karset et al., 2018)
ECHAM5.5-HAM2-ELVOC_UH	1.875° × 1.875°	31 (hybrid sigma)	GCM (nudg)	ERA-Interim, U, V, PS	(Zhang et al., 2012); (Jokinen et al., 2015)
ECHAM6-HAM2	1.875° × 1.875°	31 (hybrid sigma) to 10 hPa	GCM (nudg)	ERA-interim DIV (48h), VOR (6h) and PS (24h)	(Stevens et al., 2013), (Tegen et al., 2019), (Lohman and Neubauer , 2018)
ECHAM6-HAM2-AP	1.875° × 1.875°	31 (hybrid sigma) to 10hPa	GCM (nudg)	ERA-interim DIV (48h), VOR (6h) and PS (24h)	(Stevens et al., 2013); (Tegen et al., 2019); (Neubauer et al., 2018)
EMAC	1.875° × 1.875°	31 (hybrid sigma)	GCM (nudg)	ERA-interim DIV (48h), VOR (6h) and PS (24h)	(Jöckel et al., 2010); (Karydis et al., 2017)
GEOS-Chem-APM	2° × 2.5°	38	CTM	MERRA2	(Yu and Luo, 2009), (Yu, 2011)
GEOS-Chem-TOMAS	4.0° × 5.0°	25	CTM	MERRA	(Bey et al., 2001a) (http://acmg.seas.harvard.edu/geos/)
GISS-E2.1-MATRIX	2° x 2.5°	40	GCM (nudg)	NCEP	(Bauer et al., 2008)
GISS-E2-Tomas	2° x 2.5°	40	GISS GCM (free-running)	Online	(Lee et al., 2015)
TM4-ECPL and TM4-ECPL/v	2° x 3°	34 (hybrid sigma) to 0.1 hPa	TM4, CTM	ECMWF ERA-interim	(Kanakidou et al., 2012), (Daskalakis et al., 2015)
TM5	2° x 3°	34 (hybrid sigma) to 0.1 hPa	TM5, CTM	ECMWF ERA-Interim	(Van Noije et al., 2014), (Huijnen et al., 2010); (Berg-man et al., 2018, in prep.)

Table S2. Details about the microphysics schemes employed by the models.

MODEL	Microphysics Scheme	Scheme Type	Number of bins or modes	Reference	Aerosol components	Nucleation Scheme
**CAM5-chem-APM**	APM	Sectional (2m)	40	To be published	SO4, NO3, NH4, SOA, SS, DU, BC, POA	Ternary ion-mediated nucleation (Yu et al., 2018)
**CAM5-chem-ATRAS2**	ATRAS2	Sectional (2m)	12 (47 in maximum)	(Matsui, 2017) (Matsui and Mahowald, 2017)	SO4, NO3, NH4, OA (POA, SOA), SS, DU, BC	(Kulmala et al., 2006) within the planetary boundary later (Vehkamäki et al. 2002) within the free troposphere
**CAM5_MAM3**	MAM3	Modal (2m)	3	(Liu et al., 2012)	SO4, BC, DU, SS, OA (POA+SOA)	(Vehkamäki, 2002)
**CAM5_MAM4**	MAM4	Modal (2m)	4	(Liu et al., 2012) (Liu et al., 2016)	SO4, BC, DU, SS, POA+SOA	(Vehkamäki, 2002) . The boundary layer parameterization, (only applied in PBL), uses the empirical first order (in H_2SO_4) nucleation rate from (Sihto et al., 2006)
**CAM5.3-Oslo**	OsloAero5.3 with look-up tables from AeroTab5.3 (offline)	Production-tagged (linked to an offline 2m Sectional scheme)	44 (offline)	{Kirkevåg et al., 2018}	SO4, BC, DU, SS, OA (POA+SOA), distributed over 12 external and internal mixtures (size-modes)	(Vehkamäki, 2002), (Paasonen et al., 2010)
**ECHAM5.5-HAM2-ELVOC_UH**	HAM2	Modal (2m)	7	(Zhang et al., 2012)	SO4, BC, DU, SS, OA (POA+SOA) SOA as in (Joniken et al., 2015)	(Vehkamäki, 2002) (Paasonen et al., 2010)
**ECHAM6-HAM2**	HAM2	Modal (2m)	7	(Tegen et al. 2019); (Neubauer et al, 2018), (Zhang et al., 2012)	SO4, BC, DU, SS, OA (POA+SOA) SOA: approximated as 15% of monoterpene emissions at the surface. SOA is assumed to condense immediately on existing aerosol particles and to have identical properties to primary organic aerosols	(Kazil et al., 2010);
**ECHAM6-HAM2-AP**	AP	Modal (2m)	7+2	(Tegen et al., 2019) ; (Neubauer et al., 2014) based on (Hoose et al., 2008a, 2008b)	SO4, BC, DU, SS, OA (POA+SOA) SOA: as in ECHAM6-HAM2	(Kazil and Lovejoy, 2007); (Kazil et al., 2010); (Kulmala et al., 2006)
**EMAC**	GMXe	Modal (2m)	7	(Pringle et al., 2010a) (Pringle et al., 2010b)	Thermodynamic equilibrium using ISORROPIA-II (Fountoukis and Nenes, 2007) Species considered: K^+-Ca^{2+}-Mg^{2+}-NH_4^+-Na^+-SO_4^{-2}-NO_3^--Cl^--H_2O-BC-OA-DU-SS	(Vehkamäki, 2002)
**GEOS-Chem-APM**	APM	Sectional (1m)	40 bins for inorganic aerosols & 2 modes for BC and OA	(Yu and Luo, 2009)	SO4, NO3, NH4, SOA, SS, DU, BC, POA	Ternary ion-mediated nucleation (Yu et al., 2018)
**GEOS-Chem-TOMAS**	TOMAS	Sectional (2m)	15	(Trivitayanurak et al., 2008),	SO4, NH4, BC, DU, SS, POA, SOA	Ternary (+ammonia) (Napari et al., 2002) scaled by 1E-5

				(Kodros et al., 2016)		If no ammonia (Vehkamäki, 2002)
**GISS-E2.1-MATRIX**	MATRIX	Modal (2m)	16 mixing states	(Bauer et al., 2008)	SO4, NO3, NH4, H2O, OA, BC, DU, SS	NAPARI ternary nucleation scheme (Napari et al., 2002)
**GISS-E2-Tomas**	TOMAS	Sectional (2m)	15	(Lee et al., 2015)	SO4, BC (hydrophilic), BC (hydrophobic), OC (hydrophilic), OC (hydrophobic), DU, SS, NH4, water	Binary (Vehkamäki, 2002)
**TM4-ECPL**	M7	Modal (2m)	7	(Vignati et al., 2004)	SO4. BC, DU, SS, OA (POA+SOA) SOA: Produced from oxidation of pinene, isoprene and aromatics (xylene, benzene and toluene) with O_3, OH, NO_3.	Binary (Vehkamäki, 2002) with (Kokkola et al., 2009) scheme for the treatment of the sulfuric acid gas
**TM5**	M7	Modal (2m)	7	(Vignati et al., 2004)	SO4, BC, DU, SS, POA, SOA, NO3, NH4, MSA	Organic+ sulfuric acid nucleation (Riccobono et al., 2014); binary homogeneous nucleation (Vehkamäki, 2002)

Table S3. Emission inventories and schemes used in the models.

Anthropogenic emissions are given for the year 2011. For interactive emissions, the references are for the model used to calculate the emissions.

Model	SOx (SO_2+SO4) SOx (total) Tg a^{-1}		SO4 (emit-ted) Tg a^{-1}	BC (Tg a^{-1})		POA (Tg a^{-1})		VOC (Tg a^{-1})		SS (Tg a^{-1})		DUST (Tg a^{-1})	
**CAM5-chem-APM**	(Keller et al., 2014)	55 Tg S a^{-1}	0	(Keller et al., 2014)	7	(Keller et al., 2014)	41 TgC a^{-1}	(Lamarque et al., 2012)	Isoprene & monoter-penes 500 TgC a^{-1}	(Gong, 2003)	11100	(Mahowald et al., 2006)	3620
**CAM5-chem-ATRAS2**	(Lamarque et al., 2010)	64.8	1.66	(Lamarque et al., 2010)	7.8	(Lamarque et al., 2010)	50.3	NMVOC derived from MOZART emissions for year 2000.	115 (SOA)	Calculated on line (Mårtensson et al., 2003) (Monahan et al., 1986)	5039 (wind depend-ent flux)	Calculated online (Zender et al., 2003) (Albani et al., 2014)	2677 (wind depend-ent flux)
**CAM5_MAM3**	CMIP6 (Hoesly et al., 2017), (van Marle et al., 2017)	70.17	1.75	CMIP6 (Hoesly et al., 2017), (van Marle et al., 2017)	9.32	CMIP6 (Hoesly et al., 2017), (van Marle et al., 2017)	44.66	CMIP6 (Hoesly et al., 2017), (van Marle et al., 2017) MOZART-2 (Horowitz et al., 2003)	75.36 (SOAg)	Calculated on line (Mårtensson et al., 2003) (Monahan et al., 1986)	2581.4	Calculated online (Zender et al., 2003)	3117.7
**CAM5_MAM4**	SO_2: ACCMIP (Lamarque et al., 2013) SO4: 2.5% of SO_2	64.8	1.62	ACCMIP (Lamarque et al., 2013)	7.76	ACCMIP (Lamarque et al., 2013)	50.2	NMVOC derived from MOZART emissions for year 2000.	SOA creat-ed=103.3	Calculated on line (Mårtensson et al., 2003) for aerosols with geometric diameter < 2.8 µm (Monahan et al., 1986) for aero-sols with geo-metric diameter > 2.8 µm	Wind depend-ent flux	Online (Scanza et al., 2015), (Albani et al., 2014)	Wind depend-ent flux
**CAM5.3-Oslo**	(Lamarque et al., 2010)	66.70	1.67	(Lamarque et al., 2010)	7.93	Kirkevåg et al.. (2018)	87.03	(Guenther et al., 2006)	499.5 (iso-prene and monoter-pene only, i.e. not including MSA contri-bution)	Calculated online (Salter et al., 2015; Kirkevåg et al., 2018))	1956	Calculated online (Zender et al., 2003)	3117
**ECHAM6-HAM2**	(Lamarque et al., 2010)	69.7 Tg-S a^{-1}	1.7 Tg S a-1 (only direct emitted SO4; SO4	(Lamarque et al., 2010)	8.0	(Lamarque et al., 2010)	66.7 Tg a^{-1}	(Dentener et al., 2006)	Included in POA	(Long et al., 2011); (Sofiev et al., 2011)	Wind depend-ent flux	(Tegen et al., 2002); (Cheng et al., 2008); (Heinold et al., 2016)	Wind depen-dent flux

			produced in clouds by wet chemistry: 51.9 Tg S a-1 is not included)										
**ECHAM5.5-HAM2-ELVOC_UH**	(Lamarque et al., 2010)	53.9 Tg S a^{-1}	1.3 Tg S a^{-1}	(Lamarque et al., 2010)	7.24	(Lamarque et al., 2010)	49.3 Tg C a^{-1}	(Guenther et al., 2006)	27	(Schultz et al., 2002)	5032	(Tegen et al., 2002), (Cheng et al., 2008)	931
**ECHAM6-HAM2-AP**	(Lamarque et al., 2010)	70.1 Tg S a^{-1}	1.7 Tg S a-1 (only direct emitted SO4; SO4 produced in clouds by wet chemistry: 51.9 Tg S a-1 is not included)	(Lamarque et al., 2010)	8.1	(Lamarque et al., 2010)	47.9 Tg C a^{-1}= 67.0 Tg OA a^{-1}	(Dentener et al., 2006)	Included in POA	Calculated online (Long et al., 2011); (Sofiev et al., 2011)	1161	Calculated online (Cheng et al., 2008); (Heinold et al., 2016)	926
**EMAC**	RCP 8.5 (Riahi et al., 2007)	63.6 Tg S a^{-1}	1.6 Tg S a^{-1}	RCP 8.5 (Riahi et al., 2007)	6.7	RCP 8.5 (Riahi et al., 2007)	41.1 Tg C a^{-1}	RCP 8.5 (Riahi et al., 2007)	Included in POA	Total Sea salt: (Dentener et al., 2006) chemical Composition of sea salt: (Seinfeld and Pandis, 2006)	7889	Total dust emissions: (Astitha et al., 2012) Chemical composition of dust: (Karydis et al., 2016); (Klingmüller et al., 2018)	4760
**GEOS-Chem-APM**	(Keller et al., 2014)	55 Tg S a^{-1}	0	(Keller et al., 2014)	7	(Keller et al., 2014)	41 Tg C a^{-1}	(Keller et al., 2014)	Isoprene & monoterpenes 760 TgC a^{-1}	(Gong, 2003)	10800	Calculated online (Zender et al., 2003) (Mahowald et al., 2006)	1180
**GEOS-Chem-TOMAS**	Mixture of global and regional inventories (described in Kodros et al.,	395.36	0.27 Tg S a-1	Anthropogenic: Bond et al. (2007) Biomass burning: (Wiedinmyer et al., 2011)	6.97	Anthropogenic: (Bond et al. 2007) Biomass burning:	44.72	Biogenic VOC emissions from (Guenther et al., 2006), Anthropogenically-influenced VOCs	Biogenic SOA created=19 Tg C a-1 Anthropogenically-	Calculated online (Jaeglé et al., 2011)	Wind dependent flux	Calculated online (Zender et al., 2003)	Wind dependent flux

	2016; Keller et al., 2014)					(Wiedinmyer et al. 2011)		from (D'Andrea et al., 2013)	enhanced SOA created=100 Tg a^{-1}				
**GISS-E2.1-MATRIX**	(Lamarque et al., 2010)	191.82(SO_2)	5.27	(Lamarque et al., 2010)	9.35	(Lamarque et al., 2010)	61.73	Isoprene: (Guenther et al., 1995). Monoterpenes and ORVOC: (Lathiere et al., 2005)	Isoprene: 524 Terpenes + ORVOC: 193 SOA production 17.1	(Schmidt et al., 2014)	2662.69	(Schmidt et al., 2014)	1275.60
**GISS-E2-Tomas**	1 % of SO_2 ACCMIP (Lamarque et al., 2013)	65.6	0.66	ACCMIP and GFEDv3 for biomass burning	7.4	ACCMIP and GFEDv3 for biomass burning	43.7	ACCMIP (Lamarque et al., 2013)	SOA production rate of 17	(Gong, 2003)	3231.9	(Ginoux et al., 2001)	705.8
**TM4-ECPL and TM4-ECPL/v**	2.5% of SO2 ACCMIP (Lamarque et al., 2013)	121.25(SO_2)	4.66 (SO4)	ACCMIP (Lamarque et al., 2013)	8.02	ACCMIP (Lamarque et al., 2013)	35.12 Tg C a^{-1}	Emissions of Biogenic VOCs are taken from (Sindelarova et al., 2014) Anthropogenic VOC ACCMIP, Ocean emissions POET	Isoprene: 525 monoterpenes: 104.3 other VOC: 572.3 SOA production: 32.6	(Vignati et al., 2010)	8521	(Dentener et al., 2006)	1169.5
**TM5**	CMIP6 (Hoesly et al., 2018); (van Marle et al., 2017); GEIA (Andres and Kasgnoc, 1998)	62.04	1.59	CMIP6 (Hoesly et al., 2018); (van Marle et al., 2017)	9.49	CMIP6 (Hoesly et al., 2018); (van Marle et al., 2017)	52.02	CMIP6 (Hoesly et al., 2018); (van Marle et al., 2017); MEGAN-MACC (Sinderalova et al., 2014)	109.32	Calculated online (Gong et al., 2000); (Salisbury et al., 2013)	5497.08	Calculated online (Tegen et al., 2002)	978.88

Table S4. Biomass burning emission's injection height and dry and wet deposition parameterization in the models.

Model	Biomass Burning Emissions injection height	Dry and Wet Deposition
**CAM-chem-APM**	Emission data are produced from OC forest fire and grass fire emissions from the file IPCC_GriddedBiomassBurningEmissions_OC_decadalmonthly mean by NCAR. In January forest fire emissions extent up to 4 km in the tropics and extratropical mid-latitudes of the south hemisphere and in June they extend to 6 km at around 60 N Lamarque et al. (2010)	**Dry deposition**: Gases (Wesely, 1989) "functions of solar radiation and/or time of day", Aerosols (Zhang et al., 2001) "Dry (gravitational and turbulent) deposition velocity" **Wet deposition:** Scheme from Neu and Prather (2012) for gases "Washout of highly soluble gases by rain in ambient air is modeled as an impact scavenging process, while scavenging of moderately soluble gases from the ambient air is limited by Henry's Law equilibrium with the falling precipitation. Washout of gases from interstitial and ambient air takes place in the Mixed Cloud (MC) and Ambient (AM) fractions. We assume a constant rate of evaporation in AM, which releases gases to the environment along with any large-scale evaporation" and the scheme from Rasch et al. (2000) for aerosols "Many aerosols act as cloud condensation nuclei. That is, water vapour condenses preferentially on soluble or wettable particles to form cloud drops subsequently removed via in-cloud scavenging. Aerosols can also be taken up directly on falling precipitate through a number of collection mechanisms. Larger aerosols are taken up by collision associated with their inertia. Smaller particles are collected by Brownian motion." Washout of size resolved aerosols (Henzing et al., 2006) "A size dependent parameterization for the removal of aerosol particles by falling rain droplets has been developed. For below-cloud scavenging a source for sea salt aerosol has been adopted. or particles with diameter larger than 1μm, below-cloud scavenging is as important as the removal in convective updrafts and that below-cloud scavenging accounts for 12% of the total yearly average removal. At mid-latitudes of both hemispheres the fractional contribution of below-cloud scavenging to the total removal is about 30% with regional maxima exceeding 50%. The maxima in relative importance of below-cloud scavenging coincide with maxima in emissions. Excluding the below-cloud scavenging process would result in an increase of global average aerosol lifetime from 2.16 days to2.47 days."
**CAM5-chem-ATRAS2**	Injection height profiles for forest fire and grass fire emissions are derived from the corresponding AeroCom profiles (Dentener et al., 2006), which give emissions in 6 altitude ranges (0-0.1, 0.1-0.5, 0.5-1, 1-2, 2-3, and 3-6 km).	**Dry deposition** velocity (gravitational and turbulent) (Zhang et al., 2001) **Wet deposition** is both in-cloud scavenging of cloud-borne aerosol and the below cloud scavenging of interstitial aerosol. Reference for deposition processes (Liu et al., 2012)*. The CAM5_MAM3 model here includes additional improved treatments of convective transport and wet removal of aerosols developed by Wang et al. (2013).
**CAM5_MAM3**		
**CAM5_MAM4**		
**CAM5.3-Oslo**	Dentener et al. (2006)	**Dry deposition:** Liu et al. (2012)* **Wet deposition:** Below-cloud scavenging, using below-cloud collection efficiencies as in Seland et al. (2008), and in-cloud scavenging (Liu et al., 2012)*.
**ECHAM5.5-HAM2-ELVOC_UH**	Dentener et al. (2006)	**Dry deposition:** big leaf approach, a function of aerodynamic resistance, quasi-laminar boundary layer resistance, and surface resistance (Ganzeveld and Lelieveld,1995; Ganzeveld et al.,1998). Turbulent dry deposition of aerosols (Ganzeveld et al., 1998). **Wet deposition:** Below-cloud scavenging (Seinfeld and Pandis, 1998; Stier et al., 2005). In-cloud and below-cloud scavenging of precursor gases: Henry's law (Seinfeld and Pandis, 1998)

		Reference for deposition processes Zhang et al. (2012)
**ECHAM6-HAM2**	Injection heights of biomass burning emissions follow the recommendations of Val Martin et al. (2010). 75% of the emissions are evenly distributed within the planetary boundary layer (PBL), 17% in the first level and 8% in the second level above the PBL (Tegen et al., 2019)	Tegen et al. (2018)<br>The aerosol **dry deposition** flux is computed as the product of tracer concentration, air density and deposition velocity, depending on the aerodynamic and surface resistances for each surface type considered by ECHAM6.3, and subsequently added up for the fractional surface areas.<br>Sedimentation of aerosol particles is computed for accumulation and coarse modes (i.e. large particles) using Stokes velocity and the Cunningham slip correction factor. To ensure numerical stability the sedimentation velocity is limited by the ratio of layer thickness to model timestep.<br>Dry deposition is computed for all modes except the nucleation mode<br>For **wet deposition** the in-cloud scavenging scheme from Croft et al. (2010) dependent on the wet particle size is used. The in-cloud scavenging scheme takes into account scavenging by droplet activation and impaction scavenging in different cloud types, distinguishing between stratiform and convective clouds and warm, cold, and mixed-phase clouds. Below clouds particles are scavenged by rain and snow using a size-dependent below-cloud scavenging scheme (Croft et al., 2009).<br>In-cloud (Croft et al., 2010) and below-cloud (Croft et al., 2009) wet scavenging depends on the size of the aerosol particles.
**ECHAM6-HAM2-AP**	Injection heights of biomass burning emissions follow the recommendations of Val Martin et al. (2010). 75% of the emissions are evenly distributed within the planetary boundary layer (PBL), 17% in the first level and 8% in the second level above the PBL (Tegen et al., 2019)	Tegen et al. (2018)<br>The aerosol **dry deposition** flux is computed as the product of tracer concentration, air density and deposition velocity, depending on the aerodynamic and surface resistances for each surface type considered by ECHAM6.3, and subsequently added up for the fractional surface areas.<br>Sedimentation of aerosol particles is computed for accumulation and coarse modes (i.e. large particles) using Stokes velocity and the Cunningham slip correction factor. To ensure numerical stability the sedimentation velocity is limited by the ratio of layer thickness to model timestep.<br>Dry deposition is computed for all modes except the nucleation mode<br>For **wet deposition** the aerosol processing scheme by Hoose et al. (2008a, 2008b) is used for in-cloud wet scavenging. The aerosol processing scheme explicitly tracks the aerosol mass within cloud droplets and ice crystals and computes online the processes which add (nucleation, impaction) and remove (evaporation, rain formation) aerosol mass inside cloud droplets and ice crystals. Below clouds particles are scavenged by rain and snow using a size-dependent below-cloud scavenging scheme (Croft et al., 2009).
**EMAC**	Injection height is 140 m	**Dry deposition:** DRYDEP submodel (Kerkweg et al., 2006) based on the big leaf approach (only one receptor surface is present in the model and that turbulent exchange within the forest canopy is neglected).<br>**Wet deposition:** SCAV submodel (Tost et al., 2006); The SCAV submodel is highly structured, and all calculations are performed in the smallest meaningful entity. For the physico-chemical process ‘scavenging’ this is a vertical column since the chemical composition of the rainwater that enters a grid box from above affects the scavenging in that particular layer as well as the layers below. Within the column, the scavenging process starts in the uppermost layer where a cloud occurs (Nucleation Scavenging - NS). In the

		layer below, the Impaction Scavenging (IS) by the incoming precipitation flux is calculated first, followed by NS in that particular layer. If there are no clouds in this layer, the NS is neglected and only IS is calculated.
**GEOS-Chem-APM**	Well mixed within the planetary boundary layer	**Dry deposition**: Gases (Wesely, 1989) "functions of solar radiation and/or time of day", Aerosols (Zhang et al., 2001) "Dry (gravitational and turbulent) deposition velocity"<br>**Wet deposition** (Jacob at al., 2000) "wet convective mass fluxes and precipitation fluxes. We use this information to implement two types of scavenging: (1) scavenging in subgrid wet convective updrafts, and (2) first-order rainout and washout in precipitating columns. The scavenging is applied to aerosols and to soluble gases of interest to tropospheric O3chemistry including HNO3,H2O2,CH3OOH, and CH2O.The methodology is readily extendable to other soluble gases" and (Wang et al., 2011) "Liu et al. (2001) includes scavenging in convective updrafts, as well as in-cloud and below-cloud scavenging from convective and large-scale precipitation. However, it does not distinguish between rain and snow. Wang et al. introduce such a distinction as well as other improvements to the scavenging scheme." Washout of size resolved aerosols (Henzing et al., 2006)
**GEOS-Chem-TOMAS**	Evenly distributed throughout the boundary layer	Size-resolved dry and wet deposition (Trivitayanurak et al., 2008). Dry deposition follows a resistance in series scheme. Wet deposition considers in cloud and below cloud scavenging separately for stratiform and convective anvils.
**GISS-E2.1-MATRIX**	Evenly distributed throughout the boundary layer	**Dry deposition**: Turbulent dry deposition is based on the resistence-in- series scheme described in Koch et al. (1999) and Chin et al. (1996). The scheme is coupled to the model boundary layer scheme of the GCM and depends on the mean diameter of the aerosol population. Gravitational settling depends on the aerosol population mean diameter and density and accounts for the effects of RH on density and size.<br>**Wet deposition**: The wet deposition schemes of the GISS modelE are described in Koch et al. (1999, 2006) and Bauer et al (2008). The model treats two types of clouds, convective and stratiform clouds. Tracer treatment in clouds follows the cloud processes, so that tracers are transported, dissolved, evaporated, and scavenged (with cloud-water autoconversion and by raindrop impaction beneath clouds). This parameterization requires information about aerosol size and solubility, which are calculated for each aerosol population by MATRIX. The averaged solubility per aerosol population is calculated by using a volume weighted approach, depending on the chemical composition of the aerosol particles. Number and mass concentrations are both treated in the model's advection and deposition schemes.
**GISS-E2-Tomas**	Evenly distributed throughout the boundary layer	The dry deposition scheme is based on a resistance-in-series method derived from the Harvard GISSCTM, which is applied between the surface layer (10 m) and the ground (Koch et al., 2006). Wet deposition is determined by several processes including rainout within clouds, washout below precipitating regions, scavenging within and below cloud updrafts, evaporation of falling precipitation, transport along with convective plumes, and detrainment and evaporation from convective plumes (Koch et al., 2006; Shindell et al., 2006). Both wet and dry deposition are fully size-resolved. For in-cloud scavenging, modified Kohler theory is used to calculate the critical supersaturation for activation of aerosols.
**TM4-ECPL & TM4-ECPL/v**	Dentener et al. (2006)	**Dry deposition** for all fine aerosol components is parameterized similarly to that of nss-$SO4^{-2}$, which follows Tsigaridis et al. (2006) and the resistance model by Gazenveld et al

		(1998). Gravitational settling (Seinfeld and Pandis, 1998) is applied to all aerosol components and is an important dry deposition process for coarse particles like dust and sea salt (Myriokefalitakis et al., 2015).<br>For **wet deposition**, both largescale and convective precipitation are considered. In-cloud and below-cloud scavenging is parameterized in TM4-ECPL as described in detail by Jeuken et al. (2001). In-cloud scavenging of water-soluble gases is calculated, accounting for the solubility of the gases (effective Henry law coefficients; Tsigaridis et al., 2006; Myriokefalitakis et al., 2011 and references there in)
**TM5**	Injection heights of biomass burning follow the recommendations of Dentener et al. (2006), as described in van Noije et al. (2014). In the current study, biomass burning emissions from the CMIP6 data set are applied, and no distinction is made between forest fires and grassland fires; all biomass burning emissions are distributed as forest fires.	Dry and wet depositions follow the description given in van Noije et al. (2014) and de Bruine et al. (2018). **Dry deposition** of gases and aerosols uses a standard resistance approach (e.g. Seinfeld and Pandis, 2006). For gases the deposition velocities are calculated as the inverse of the sum of an aerodynamic resistance, a quasi-laminar sub-layer resistance and a surface resistance, while for aerosols the velocities are determined by the aerodynamic resistance and quasi-laminar sublayer resistance, enhanced by gravitational settling.<br>The **wet deposition** of gases and aerosols by rain is calculated separately for convective and large-scale stratiform precipitation. Scavenging in convective clouds is part of the convective mass transport operator (Balkanski et al., 1993; Guelle et al. 1998). Scavenging in and below stratiform clouds is calculated following Roelofs and Lelieveld (1995) and Jeuken et al. (2001), with updated removal efficiencies for aerosols as given in de Bruine et al. (2018).

*** Liu et al., 2012**

Wet Removal

Aerosol wet removal is calculated using the CAM3.5 wet removal routine (Barth et al., 2000; Rasch et al., 2000) with modifications noted below. The routine treats in-cloud scavenging (the removal of cloud-borne AP) and below-cloud scavenging (the removal of interstitial AP by precipitation particles through impaction and Brownian diffusion). For in-cloud scavenging, the precipitation production rates (kg kg^{-1} s^{-1}) and cloud water mixing ratios (kg kg^{-1}) for the stratiform and convective clouds are used to calculate first-order loss rates (s^{-1}) for cloud water. These cloud-water first-order loss rates are multiplied by “wet removal adjustment factors” (or tuning factors) to obtain aerosol first-order loss rates, which are applied to activated aerosols within the non-ice cloudy fractions of a grid cell (i.e., cloudy fractions that contain some cloud water). The stratiform in-cloud scavenging only affects the explicitly treated stratiform-cloud-borne 12 AP, and the adjustment factor of 1.0 is currently used. It does not affect the interstitial AP. In-cloud scavenging in ice clouds (i.e., clouds with no liquid water) is not treated. For convective in-cloud scavenging of MAM aerosols, the cloud-borne aerosol mixing ratios within the convective clouds are needed. These are set to the product (lumped interstitial aerosol mixing ratio) × (convective-cloud activation fraction), and we again note that the model’s lumped interstitial aerosol mixing ratios include the truly interstitial AP and the convective cloud-borne AP. The convective-cloud activation fractions are currently set to 0.0 for the primary carbon mode, 0.4 for the fine and coarse dust modes, and 0.8 for other modes. The lower values reflect lower hygroscopicity. These factors are applied to both number and mass species within each mode, with one exception. In MAM3, different activation fractions are applied to the dust and sea salt of the coarse mode (0.4 and 0.8 respectively), and a weighted average is applied to the coarse mode sulfate and number. A wet-removal adjustment factor of 0.5 is used for the convective in-cloud scavenging. The stratiform-cloud-borne AP reside in the stratiform clouds and are assumed to not interact with convective clouds. For below-cloud scavenging of the interstitial aerosol, the first-order

removal rate is equal to the product (scavenging coefficient) × (precipitation rate). The scavenging coefficient is calculated using the continuous collection equation (e.g., Equation 2 of Wang et al., 2011), in which the rate of collection of a single aerosol particle by a single precipitation particle is integrated over the aerosol and precipitation particle size distributions, at a precipitation rate of 1 mm h^{-1}. Collection efficiencies from Slinn (1984) and a Marshall-Palmer precipitation size distribution are assumed. The scavenging coefficient varies strongly with particle size, with lowest values for the accumulation 13 mode. The wet removal adjustment factor is currently 0.1. There is no below-cloud scavenging of stratiform-cloud-borne aerosol. Aerosol that is scavenged at one altitude can be re-suspended at a lower altitude if precipitation evaporates. A fraction of the in-cloud scavenged aerosol is re-suspended, and the re-suspended fraction is equal to the fraction of precipitation that evaporates below cloud.

Dry Deposition

Aerosol dry deposition velocities are calculated using the Zhang et al. (2001) parameterization with the CAM5 land-use and surface layer information. Gravitational settling velocities are calculated at all vertical layers above the surface following Seinfeld and Pandis (1998). Both velocities depend on particle wet size, so average values for aerosol mass and number are calculated for each mode. The velocities for cloud-borne aerosols are calculated based on droplet sizes. Aerosol mixing ratio changes and fluxes from dry deposition and sedimentation throughout a vertical column are then calculated using the CAM3 dust deposition/sedimentation routine (Zender et al., 2003).

Table S5. Measurement sites included in this study.

The classification and location of the stations are denoted, the supersaturation values at which CCN concentrations are measured at each site and the aerosol components for which mass is measured in the PM_1. The followed notation is: CCN_x: CCN at *x* % supersaturation, SO4: sulphate, OA: organic aerosols, SS: sea salt, NO3: Nitrates, NH4: ammonium. Details on observations, methods and data coverage can be found in Schmale et al. (2017).

**Station name**	**Location/type**	**Geoposition**	**Observations**
**Cabauw**	Cabauw, The Netherlands near coast, rural-background	51°58'N, 04°56'E, -1 m	$CCN_{0.1}$, $CCN_{0.2}$, $CCN_{0.3}$, $CCN_{0.5}$, $CCN_{1.0}$, SO4, OA, SS, NH4, NO3
**Finokalia**	Finokalia, Crete, Greece, coastal background, Mediterranean	35°20'N, 25°40'E, 260 m	$CCN_{0.2}$, $CCN_{0.4}$, $CCN_{0.6}$, $CCN_{0.8}$, $CCN_{1.0}$, SO4, OA, SS, NH4, NO3
**Jungfraujoch**	Jungfraujoch, high alpine, back-ground Switzerland	46°33'N, 07°59'E, 3580 m	$CCN_{0.1}$, $CCN_{0.15}$, $CCN_{0.2}$, $CCN_{0.25}$, $CCN_{0.3}$, $CCN_{0.35}$, $CCN_{0.4}$, $CCN_{0.5}$, $CCN_{0.7}$, $CCN_{1.0}$, SO4, OA, NH4, NO3
**Mace Head**	Mace Head, Ireland, coastal back-ground	53°19'N, -9°54'E, 5 m	$CCN_{0.1}$, $CCN_{0.25}$, $CCN_{0.35}$, $CCN_{0.5}$, $CCN_{0.75}$, $CCN_{1.0}$, SO4, OA, SS, NH4, NO3
**Melpitz**	Melpitz, Germany, continental, background	51°32'N, 12°56'E, 89 m	$CCN_{0.1}$, $CCN_{0.2}$, $CCN_{0.3}$, $CCN_{0.5}$, $CCN_{0.7}$, SO4, OA, SS, NH4, NO3
**Noto Peninsula**	Noto Peninsula, Japan, coastal background	37°45'N, 137°36'E, 0 m	$CCN_{0.1}$, $CCN_{0.2}$, $CCN_{0.5}$, $CCN_{0.8}$
**Puy de Dôme**	Puy de Dôme, France, mountain continental background	45°46'N, 2°57'E, 1465 m	$CCN_{0.2}$
**Hyytiälä**	Hyytiälä, Finland, background boreal forest rural	61°51'N, 24°17'E, 181 m	$CCN_{0.1}$, $CCN_{0.2}$, $CCN_{0.3}$, $CCN_{0.5}$, $CCN_{1.0}$, SO4, OA, SS, NH4, NO3
**Vavihill**	Vavihill, Sweden, background rural	56°01'N, 13°09'E, 172 m	$CCN_{0.1}$, $CCN_{0.15}$, $CCN_{0.2}$, $CCN_{0.25}$, $CCN_{0.3}$, $CCN_{0.35}$, $CCN_{0.4}$, $CCN_{0.5}$, $CCN_{0.7}$, $CCN_{1.0}$, $CCN_{1.4}$

Appendix S2

S2.1.Particle numbers, CCN calculations

Number concentrations of particles of various sizes (N_{50}, N_{80} and N_{120}) are computed by 14 models for all observational sites. For the modal models, where lognormal distributions are used to describe internally or externally mixed particle populations, the total number of particles with dry diameters larger than a threshold diameter D_0 is given by

$$N(D > D_0) = \sum_{i=1}^{k} \frac{N_0^i}{2}(1 - \mathrm{erf}\left(\frac{\ln\left(D_0/\widetilde{D_n^i}\right)}{\sqrt{2}}\right)) \qquad (1)$$

where k is the number of modes, N_0^i and $\widetilde{D}_n^i$ are the number of particles and the particle number median diameter of mode i, respectively, while erf is the error function. In several models calculation of the CCN number concentration is based on the κ-Köhler theory as developed by (Petters et al, 2007), according to which, for a given supersaturation s, the critical dry diameter D_d of activated particles is given by

$$s(D) = \frac{D^3 - D_d^3}{D^3 - D_d^3(1-\kappa)} \exp\left(\frac{4\sigma_{s/a}M_w}{RT\rho_w}\right) \qquad (2)$$

Here D and D_d are the wet and dry diameters of particles, respectively, $\sigma_{s/a}$ is the surface tension of the solution/air interface, which is taken equal to 0.072 J m^{-2}, ρ_w is the density of water, M_w is the molecular weight of water, R is the ideal gas constant and T is the temperature. After having determined the critical dry diameter from Eq. (2), the number of CCN is calculated from Eq. (1) as the number of particles with dry diameters larger than this critical value.

The hygroscopicity, κ, for the particle as a whole, or just for the coating layer if it is sufficiently thick for models that take into account coated particles, is determined based on the volume weighted hygroscopicities of the individual components, κ_j, where ε_i is the fraction of the dry aerosol volume occupied by the individual components, j.

$$\kappa = \sum_j \varepsilon_j \kappa_j \qquad (3)$$

In the present study, different hygroscopicity parameters are used in each model as summarized in Table 1

S2.2. Persistence – autocorrelation function

According to the mathematical definition for a stationary time series of values Y_1, Y_2, ..., Y_N at the time instances t_1, t_2, ..., t_N, the autocorrelation function (ACF) at time lag k is given by

$$r_k = \frac{\sum_{i=1}^{N-k+1}(Y_i - \bar{Y})(Y_{i+k} - \bar{Y})}{\sum_{i=1}^{N+1}(Y_i - \bar{Y})^2} \quad (4)$$

where $\bar{Y}$ is the average value of Y_i. The large-lag standard error is computed as the square root of the variance given by:

$$Var(r_k) = \frac{1}{N}\left(1 + 2\sum_{i \simeq 1}^{k} r_i^2\right) \quad (5)$$

where r_i is the ACF at time i. The persistence time can then be defined as the time lag at which the ACF crosses the curve of the large-lag standard error, i.e. when the ACF is significant compared to the standard error (Schmale et al., 2018).

Appendix S3. Results

S3.1 CCN persistence- Individual model's behavior and sensitivity to size of the emitted particles

It is worth noting here that the number concentration of CCN is not directly related to the persistence time. For example, based on the observational data at the Melpitz station, the persistence time of $CCN_{0.2}$ during winter is 3.5 times longer than during summer (7 days and 2 days respectively; Fig. 7), although the $CCN_{0.2}$ number concentration during winter (~1500 cm^{-3}) is only 25% higher than during summer (1200 cm^{-3}) (Fig. 3). This is because the ACF and the subsequently computed persistence are related to the rate of change of the CCN number concentration due to the various formation, transport and removal processes, and not to the absolute level of CCN. As such, when the CCN population remains almost unchanged for a long time period, the persistence is high, independent of the CCN concentrations.

For the Vavihill station, in contrast to the observations, all models (except EMAC) calculate somewhat longer persistence during winter. However, the observed ratio at this station has been assumed to reflect a peculiarity of the observation period during 2013 and 2014 (Schmale et al., 2018). The same behavior is also observed at the Noto Peninsula station. Most of the models, in agreement with observations, simulate persistence of ~ 2 days during summer, but none of them is able to simulate the longer persistence observed during winter.

For the remaining stations, the comparisons show similar performances for all models in terms of the winter/summer persistence. For Melpitz, Cabauw and Hyytiälä, longer persistence is observed during winter and can be ascribed to the more stable weather conditions during this season. For these stations, it appears that only four out of the 14 models (GEOS-Chem-APM, CAM5-MAM3, CAM5-MAM4, and CAM5-chem-ATRAS2) correctly simulate the winter/summer relative order of persistence, although the model persistence is smaller than the observationally derived values. These models also all use MERRA, rather than ERA which is used in the other models. At Finokalia, the longer persistence observed during summer is attributed to the more stable weather conditions during summer. This behavior is reproduced by all models but five (CAM5-Chem-APM, CAM5.3-Oslo, EMAC, GISSE2.1-MATRIX, TM4-ECPL).

Analyzing the reasons that affect the persistence and then attributing the differences between the observed and the model-derived values to the underlying physical and/or chemical process parameterizations in each model is a demanding

task which most probably is model dependent. Here a first attempt is made by examining the sensitivity of the persistence to the size of the emitted OA and BC particles. For that, we first focus on the Finokalia station and we examine the results obtained from two different simulations using the TM4-ECPL model. The setups of the two simulations are almost identical differing only in the size of the emitted OA and BC particles (details in the caption of Figure S5). Since the total OA and BC emitted masses are kept constant, changing the emitted particle sizes affects the number of emitted particles. Overall, in the new sensitivity simulation larger but fewer particles are emitted compared to the base simulation. The $CCN_{0.2}$ persistences computed from the results of the two simulations are compared with the observations in Figure S5a. In the sensitivity simulation (green bars, larger particles) an increase of the persistence during summer by a factor > 2 is seen compared to the base simulation (red bars). This increase leads to an inversion of the winter/summer ratio of persistence since during winter the persistence time remained almost unchanged. The results of the sensitivity simulation are in better agreement with observations and demonstrate the importance of the size distribution of the emitted particles for the CCN simulations and also suggest that the size is probably underestimated in the base case simulation of TM4-ECPL.

In order to identify the factors that caused this change in the relative order of the persistence at Finokalia, we have calculated the ACF of the $CCN_{0.2}$ number concentrations (Figures S5b, S5c). The black lines show the observational data during summer (solid line) and winter (dashed line) and the red lines show the results of the base simulation with TM4-ECPL, obtained for the same time-periods with the observations (Fig. S5b), while the green lines show the results of the sensitivity simulation (Fig. S5c). It is seen that for short time-periods (<15 h) both the observational and model derived ACF (denoted as ACF^{obs} and ACF^{model}, respectively) smoothly decrease. Since the $ACF^{model} > ACF^{obs}$ during that time ($t < 15$ h) the simulated CCN population varies less than the observed one. The fact that both ACF^{model} in the base simulation and ACF^{obs} vary less during winter than during summer, suggests that both model- and observationally-computed persistence are higher in winter. However, during summer, after the first ~24 hours a local maximum is present in both ACF^{model} and ACF^{obs} that shows a periodicity of 24 hours. This peak indicates a daily periodic phenomenon and can be attributed to the almost constant weather conditions during summer that drive a diurnal cycle in the photochemical formation of H_2SO_4 in the gas phase in the Finokalia region (Mihalopoulos et al., 2007). During summer a significant difference between the ACF^{model} from the base simulations and ACF^{obs} is found at longer correlation times ($t > 15$ h), when the ACF^{obs} decays much slower than the ACF^{model} leading to larger observed than modeled persistence. This can be attributed to the different production/loss rates ratio of the $CCN_{0.2}$ particles. Possible explanations are related to the aerosol aging, wet deposition and sedimentation removal, long-range transport, or model resolution that affect the lifetime of aerosols in the atmosphere.

Figure S5c depicts the ACFs of the sensitivity simulation (green lines) and the observations. Comparing the results of the two simulations (panels b and c), it is seen that during winter the two ACF^{model} are similar, while large differences are found between the two ACF^{model} during summer. This is due to the slower decay of ACF^{model} in the sensitivity simulation leading to a higher persistence time, similar to the observed one. For Cabauw station (Fig. S5d) that is affected by fresh primary emissions, the sensitivity simulation shows better agreement in persistence time with the observationally derived values; while for Mace Head station (Fig. S5f) that is received air masses transported over the ocean the two simulations

provide similar results. For Hyytiälä (Fig. S5e), the adopted change in the size of emitted particles is not sufficient to reconcile model-derived and observationally-derived persistence times, indicating that other factors might be also important for this discrepancy.

S3.2. Statistics for the comparisons between model results and observations

The following statistical variables have been computed here to quantify the agreement of model results with the observations.

Mean Error (ME)

$$\mathrm{ME} = \frac{1}{N}\sum_{i=1}^{N}(P_i - O_i)$$

Normalized Mean Bias (NMB)

$$\mathrm{NMB} = \frac{\sum_{i=1}^{N}(P_i - O_i)}{\sum_{i=1}^{N} O_i} \times 100\%$$

Normalized Mean Error (NME)

$$\mathrm{NME} = \frac{\sum_{i=1}^{N}|P_i - O_i|}{\sum_{i=1}^{N} O_i} \times 100\%$$

Root Mean Square Error (RMSE)

$$\mathrm{RMSE} = \sqrt{\frac{1}{N}\sum_{i=1}^{N}(P_i - O_i)^2}$$

Index of agreement

$$\mathrm{IOA} = 1 - \frac{\sum_{i=1}^{N}(P_i - O_i)^2}{\sum_{i=1}^{N}(|O_i - \bar{O}| + |P_i - \bar{O}|)^{2}}$$

Mean absolute Error (MAE)

$$\mathrm{MAE} = \frac{1}{N}\sum_{i=1}^{N}|P_i - O_i|$$

The results are shown in Table S6 (separate file).

S3.3. Surface PM1 composition - Global distributions

The global surface distributions of the multi-model mean of the various chemical compounds (SO4, BC, OA, SS and DU) concentrations that contribute to PM_1 as the median of the 15 models are shown in supplementary Figure S15 (left columns)

together with the corresponding model diversities (right column); while Figures S6-S14 show the results of individual models. Note that all simulated PM_1 component diversities maximize south of 60°S and north of 60°N, which reflects the challenges of the models in simulating atmospheric circulation and chemistry close to the poles. Since sulphate is formed by oxidation of sulfur dioxide (SO_2), which is mainly produced from anthropogenic activities, high concentrations of sulphate are observed in industrialized regions. In Eastern China, where the highest annual concentration of SO4 is computed, the concentration reaches 12.5 μg m^{-3}, in India 7.0 μg m^{-3}, around the Mediterranean basin 5.0 μg m^{-3} and in the eastern United States 3.0 μg m^{-3}. In marine regions, where SO_2 is formed predominantly through oxidation of dimethyl sulfide (DMS), the sulphate concentrations are significantly lower and do not exceed 1.0 μg.m^{-3} in the Northern Hemisphere and 0.6 μg m^{-3} in the Southern Hemisphere. The highest diversities are calculated for the high latitude oceanic regions in both hemispheres. In continental regions the highest diversities of 5 to 10 are found far from the main SO_2 production sources, in South America, Central Africa and Indonesia. Mann et al. (2014) pointed out that the model diversities are higher in northern Europe than in southern Europe, while in Figure 12b the opposite is seen for the present study.

BC is entirely produced by combustion of fossil fuel and biomass, including wild-fires, and volcanoes (Bycenkiene et al., 2013). Therefore, BC is observed in high amounts over continents and in significantly lower amounts over the ocean. Mass concentrations reach a maximum in Eastern China (~4.5 μgC m^{-3}) and in India (~2.0 μgC m^{-3}), while in other continental regions the annual average concentrations do not exceed 1.0 μg m^{-3} and over the ocean 0.1 μg m^{-3}. Besides polar and high-latitude regions, high diversities are also observed over the ocean and in continental regions away from the sources. Model differences can be attributed to the emissions used in the models, which differ in strength, spatial distribution, and the assumed size of the emitted particles, as well as to the removal processes, such as wet and dry deposition. Indeed, high diversity is computed over biomass burning regions in tropical South America, Africa and Indonesia but also in Saudi Arabia where oil extraction plants exist. Differences in the assumed organic mass to organic carbon ratio in the models could explain some of this model diversity. High diversities over the tropical oceans in the outflow of biomass burning regions are also associated with very low BC concentrations, differences in the transport and in the deposition patterns. The highest diversities are, however, computed close to the poles where BC levels are very low. These results are in agreement with Mann et al. (2014) who attributed model differences mainly to removal processes.

Modeling OA is more challenging than SO4 and BC, since it involves the treatment of both primary emissions as well as the secondary formation of organics from precursor gases. Model diversities are largest over the tropical oceans as well as the southern oceans suggesting that differences in the marine source of OA (or lack of such source) are the major contributors to this diversity. Similar to BC, smaller diversities are found over and downwind of biomass burning regions and in the Arabian Peninsula where oil combustion emissions prevail. Tsigaridis et al. (2014) also revealed large differences between the models in the organics source strength and formation of SOA, which reached almost an order of magnitude. That study also investigated the models' treatment of the removal of OA and found large inter-model differences especially in the strength of the wet deposition. They also found that the diversity between the models has maxima over oceanic regions south of 30°S; part of this diversity can be attributed to the marine source representation of OA in the models. In the present

study, the model diversity is slightly higher than in the earlier study over these regions, while the global surface distribution of OA shows a similar pattern.

The SS concentration (Fig. S15g), which is driven by wind speed and temperature, has maxima over the Southern Ocean. It also reveals some advection of SS over land. However, due to differences in the source parameterization and strength as well as in the deposition, large diversities (>10) between models (Fig. S15h) are computed for the Southern Ocean as well as at the Polar regions. Similarly high diversity is found over the Caspian Sea, which most probably is not properly resolved by many models, as well as over the northern part of South America where sea salt concentrations are low resulting from little inland transport of SS.

In line with these remarks, submicron dust aerosol (Fig. 15i), which is a minor contributor to the total dust emissions, has maxima over source regions with the highest concentrations over the Sahara and Gobi deserts. However, emissions from South African, Australian, Patagonia, Arizona and Great Basin deserts are also clearly seen in the DU distribution. The largest model diversities (Fig. S10j) are found to be associated with long-range transport and deposition towards the poles and in the tropical Pacific, as well as with emissions from the Patagonia desert, which seem not to be well represented in all models.

References

Adams, P. J. and Seinfeld, J. H.: Predicting global aerosol size distributions in general circulation models, J. Geophys. Res. Atmos., 107(19), 1–23, doi:10.1029/2001JD001010, 2002.

Albani, S., Mahowald, N. M., Perry, A. T., Scanza, R. A., Zender, C. S., Heavens, N. G., Maggi, V., Kok, J. F. and Otto-Bliesner, B. L.: Improved dust representation in the Community Atmosphere Model, J. Adv. Model. Earth Syst., 6(3), 541–570, doi:10.1002/2013MS000279, 2014.

Andres, R. J., and Kasgnoc, A. D.: A time-averaged inventory of subaerial volcanic sulfur emissions, J. Geophys. Res., 103(D19), 25251–25261, doi: 10.1029/98JD02091, 1998.

Astitha, M., Lelieveld, J., Abdel Kader, M., Pozzer, A. and De Meij, A.: Parameterization of dust emissions in the global atmospheric chemistry-climate model EMAC: Impact of nudging and soil properties, Atmos. Chem. Phys., 12(22), 11057–11083, doi:10.5194/acp-12-11057-2012, 2012.

Balkanski, Y. J., Jacob, D. J., Gardner, G. M., Graustein, W. C., and Turekian, K. K.: Transport and residence times of tropospheric aerosols inferred from a global three-dimensional simulation of 210Pb, J. Geophys. Res., 98, 20573–20586, 1993.

Bauer, S. E., Wright, D. L., Koch, D., Lewis, E. R., McGraw, R., Chang, L.-S., Schwartz, S. E. and Ruedy, R.: MATRIX (Multiconfiguration Aerosol TRacker of mIXing state): an aerosol microphysical module for global atmospheric models, Atmos. Chem. Phys., 8(20), 6003–6035, doi:10.5194/acp-8-6003-2008, 2008.

Bauer, S. E., & Menon, S.: Aerosol direct, indirect, semidirect, and surface albedo effects from sector contributions based on the IPCC AR5 emissions for preindustrial and present-day conditions. Journal of Geophysical Research Atmospheres, 117(1), 1–15. http://doi.org/10.1029/2011JD016816, 2012.

Bauer, S. E., Ault, A., & Prather, K. A.: Evaluation of aerosol mixing state classes in the GISS modelE-MATRIX climate model using single-particle mass spectrometry measurements. Journal of Geophysical Research Atmospheres, 118(17), 9834–9844. http://doi.org/10.1002/jgrd.50700, 2013.

Bey, I., D. J. Jacob, R. M. Yantosca, J. A. Logan, B. Field, A. M. Fiore, Q. Li, H. Liu, L. J. Mickley, and M. Schultz, Global modeling of tropospheric chemistry with assimilated meteorology: Model description and evaluation, J. Geophys. Res., 106, 23,073-23,096, 2001a.

Bergman, T., Makkonen, R., Schrödner, R., van Noije, T., et al.: Evaluation of a secondary organic aerosol scheme in TM5, in prep.

Bond, T. C., Bhardwaj, E., Dong, R., Jogani, R., Jung, S., Roden, C., … Trautmann, N. M.: Historical emissions of black and organic carbon aerosol from energy-related combustion, 1850-2000. Global Biogeochemical Cycles, 21(2). https://doi.org/10.1029/2006GB00284, 2007.

Cheng, T., Peng, Y., Feichter, J. and Tegen, I.: An improvement on the dust emission scheme in the global aerosol-climate model ECHAM5-HAM, Atmos. Chem. Phys., 8, https://doi.org/10.5194/acp-8-1105-2008, 1105– 1117, 2008.

Chin, M., M., D. J. J., Gardner, G. M., Foreman-Fowler, M. S., Spiro, P. A., and Savoie, D. L.: A global three-dimensional model of tropospheric sufate, J. Geophys. Res., 101, 18667– 18 690, 1996.

Croft, B., Lohmann, U., Martin, R. V., Stier, P., Wurzler, S., Feichter, J., Posselt, R., and Ferrachat, S.: Aerosol size-dependent below-cloud scavenging by rain and snow in the ECHAM5-HAM, Atmospheric Chemistry and Physics, 9, 4653–4675, 2009.

Croft, B., Lohmann, U., Martin, R. V., Stier, P., Wurzler, S., Feichter, J., Hoose, C., Heikkilä, U., van Donkelaar, A., and Ferrachat, S.: Influences of in-cloud aerosol scavenging parameterizations on aerosol concentrations and wet deposition in ECHAM5-HAM, Atmospheric Chemistry and Physics, 10, 1511–1543, 2010.

D'Andrea, S. D., Häkkinen, S. A. K., Westervelt, D. M., Kuang, C., Levin, E. J. T., Kanawade, V. P., Leaitch, W. R., Spracklen, D. V., Riipinen, I. and Pierce, J. R.: Understanding global secondary organic aerosol amount and size-resolved condensational behavior, Atmos. Chem. Phys., 13(22), 11519–11534, doi:10.5194/acp-13-11519-2013, 2013.

De Bruine, M., Krol, M., van Noije, T., Le Sager, P., and Röckmann, T.: The impact of precipitation evaporation on the atmospheric aerosol distribution in EC-Earth v3.2.0, Geosci. Model Dev., 11, 1443-1465, https://doi.org/10.5194/gmd-11-1443-2018, 2018.

Daskalakis, N., Myriokefalitakis, S. and Kanakidou, M.: Sensitivity of tropospheric loads and lifetimes of short lived pollutants to fire emissions, Atmos. Chem. Phys., 15(6), 3543–3563, doi:10.5194/acp-15-3543-2015, 2015.

Dentener, F., Kinne, S., Bond, T., Boucher, O., Cofala, J., Generoso, S., Ginoux, P., Gong, S., Hoelzemann, J. J., Ito, A., Marelli, L., Penner, J. E., Putaud, J. P., Textor, C., Schulz, M., Van Der Werf, G. R. and Wilson, J.: Emissions of primary aerosol and precursor gases in the years 2000 and 1750 prescribed data-sets for AeroCom, Atmos. Chem. Phys., 6(12), 4321–4344, doi:10.5194/acp-6-4321-2006, 2006.

Fountoukis, C. and Nenes, A.: ISORROPIA II: a computationally efficient thermodynamic equilibrium model for K+–Ca2+–Mg2+–NH4+–Na+–SO42−–NO3, Atmos. Chem. Phys., 7(17), 4639–4659, doi:10.5194/acp-7-4639-2007, 2007.

Ganzeveld, L., and Lelieveld J.: Dry deposition parameterization in a chemistry general circulation model and its influence on the distributionof reactive trace gases,J. Geophys. Res.,100, 20,999–21,012, 1995.

Ganzeveld, L., Lelieveld, J., and Roelofs, G. J.: Dry deposition parameterization of sulfur oxides in a chemistry and general circulation, J. Geophys. Res., 103, 5679–5694, doi:10.1029/97JD03077, 1998.

Ginoux, P., Chin, M., Tegen, I., Prospero, J. M., Holben, B., Dubovik, O. and Lin, S.-J.: Sources and distributions of dust aerosols simulated with the GOCART model, J. Geophys. Res. Atmos., 106(D17), 20255–20273, doi:10.1029/2000JD000053, 2001.

Gong, S. L.: A parameterization of sea-salt aerosol source function for sub- and super-micron particles, Global Biogeochem. Cycles, 17(4), n/a-n/a, doi:10.1029/2003GB002079, 2003.

Guelle, W., Balkanski, Y. J., Schulz, M., Dulac, F., and Monfray, P.: Wet deposition in a global size-dependent aerosol transport model: 1. Comparison of a 1 year 210Pb simulation with ground measurements, J. Geophys. Res., 103, 11429–11445, 1998.

Guenther, A., Karl, T., Harley, P., Wiedinmyer, C., Palmer, P. I. and C., G.: Estimates of global terrestrial isoprene emissions using MEGAN, Atmos. Chem. Phys., 6(1), 107–173, doi:10.5194/acpd-6-107-2006, 2006.

Heinold, B., Tegen, I., Schepanski, K. and Banks, J. R.: New developments in the representation of Saharan dust sources in the aerosol-climate model ECHAM6-HAM2, Geosci. Model Dev., 9(2), 765–777, doi:10.5194/gmd-9-765-2016, 2016.

Henzing, J. S., Olivi´e, D. J. L., and van Velthoven, P. F. J., A parameterization of size resolved below cloud scavenging of aerosol by rain, Atmos. Chem. Phys., 6, 3363–3375, doi:10.5194/acp-6-3363-2006, 2006.

Hoesly, R. M., Smith, S. J., Feng, L., Klimont, Z., Janssens-Maenhout, G., Pitkanen, T., Seibert, J. J., Vu, L., Andres, R. J., Bolt, R. M., Bond, T. C., Dawidowski, L., Kholod, N., Kurokawa, J.-I., Li, M., Liu, L., Lu, Z., Moura, M. C. P., O'Rourke, P. R. and Zhang, Q.: Historical (1750–2014) anthropogenic emissions of reactive gases and aerosols from the

Community Emissions Data System (CEDS), Geosci. Model Dev., 369-408, doi:10.5194/gmd-11-369-2018, 2018.
Hoose, C., Lohmann, U., Stier, P., Verheggen, B. and Weingartner, E.: Aerosol processing in mixed-phase clouds in ECHAM5-HAM: Model description and comparison to observations, J. Geophys. Res., 113(D7), D07210, doi:10.1029/2007JD009251, 2008a.
Hoose, C., Lohmann, U., Bennartz, R., Croft, B. and Lesins, G.: Global simulations of aerosol processing in clouds, Atmos. Chem. Phys., 8(23), 6939–6963, doi:10.5194/acp-8-6939-2008, 2008b.
Horowitz, L. W., Walters, S., Mauzerall, D. L., Emmons, L. K., Rasch, P. J., Granier, C., Tie, X., Lamarque, J.-F., Schultz, M. G., Tyndall, G. S., Orlando, J. J. and Brasseur, G. P.: A global simulation of tropospheric ozone and related tracers: Description and evaluation of MOZART, version 2, J. Geophys. Res. Atmos., 108(D24), n/a-n/a, doi:10.1029/2002JD002853, 2003.
Huijnen, V., Williams, J., Van Weele, M., Van Noije, T., Krol, M., Dentener, F., Segers, a., Houweling, S., Peters, W., De Laat, J., Boersma, F., Bergamaschi, P., Van Velthoven, P., Le Sager, P., Eskes, H., Alkemade, F., Scheele, R., Nédélec, P. and Pätz, H. W.: The global chemistry transport model TM5: Description and evaluation of the tropospheric chemistry version 3.0, Geosci. Model Dev., 3(2), 445–473, doi:10.5194/gmd-3-445-2010, 2010.
Jacob, D. J., Liu, H., Mari, C., and Yantosca, B. M.: Harvard wet deposition scheme for GMI, acmg.seas.harvard.edu/geos/wiki_docs/deposition/wetdep.jacob_etal_2000.pdf, 2000.
Jaeglé, L., Quinn, P. K., Bates, T. S., Alexander, B., & Lin, J.-T.: Global distribution of sea salt aerosols: new constraints from in situ and remote sensing observations. Atmospheric Chemistry and Physics, 11(7), 3137–3157. https://doi.org/10.5194/acp-11-3137-2011, 2011.
Jeuken, A., Veefkind, J. P., Dentener, F., Metzger, S., and Gonzalez, C. R.: Simulation of the aerosol optical depth over Europe for August 1997 and a comparison with observations, J. Geophys. Res., 106, 28295–28311, 2001.
Jöckel, P., Kerkweg, A., Pozzer, A., Sander, R., Tost, H., Riede, H., Baumgaertner, A., Gromov, S. and Kern, B.: Development cycle 2 of the Modular Earth Submodel System (MESSy2), Geosci. Model Dev., 3(2), 717–752, doi:10.5194/gmd-3-717-2010, 2010.
Jokinen T., Berndt T., Makkonen R., Kerminen V.-M., Junninen H., Paasonen P., Stratmann F., Herrmann H., Guenther A.B., Worsnop D.R., Kulmala M., Ehn M., Sipilä M., Production of extremely low volatile organic compounds from biogenic emissions: Measured yields and atmospheric implications, PNAS, 112 (23) 7123-7128, 2015
Kanakidou, M., Duce, R. A., Prospero, J. M., Baker, A. R., Benitez-Nelson, C., Dentener, F. J., Hunter, K. A., Liss, P. S., Mahowald, N., Okin, G. S., Sarin, M., Tsigaridis, K., Uematsu, M., Zamora, L. M. and Zhu, T.: Atmospheric fluxes of organic N and P to the global ocean, Global Biogeochem. Cycles, 26(3), 1–12, doi:10.1029/2011GB004277, 2012.
Karset, I. H. H., Berntsen, T. K., Storelvmo, T., Alterskjær, K., Grini, A., Olivié, D., Kirkevåg, A., Seland, Ø., Iversen, T., and Schulz, M.: Strong impacts on aerosol indirect effects from historical oxidant changes, Atmos. Chem. Phys., 18, 7669–7690, https://doi.org/10.5194/acp-18-7669-2018, 2018.
Karydis, V. A., Tsimpidi, A. P., Pozzer, A., Astitha, M. and Lelieveld, J.: Effects of mineral dust on global atmospheric nitrate concentrations, Atmos. Chem. Phys., 16(3), 1491–1509, doi:10.5194/acp-16-1491-2016, 2016.
Karydis, V. A., Tsimpidi, A. P., Bacer, S., Pozzer, A., Nenes, A., & Lelieveld, J. : Global impact of mineral dust on cloud droplet number concentration. Atmospheric Chemistry and Physics, 17(9), 5601–5621. http://doi.org/10.5194/acp-17-5601-2017, 2017.
Kazil, J. and Lovejoy, E. R.: A semi-analytical method for calculating rates of new sulphate aerosol formation from the gas phase, Atmos. Chem. Phys., 7(13), 3447–3459, doi:10.5194/acp-7-3447-2007, 2007.
Kazil, J., Stier, P., Zhang, K., Quaas, J., Kinne, S., O'Donnell, D., Rast, S., Esch, M., Ferrachat, S., Lohmann, U. and Feichter, J.: Aerosol nucleation and its role for clouds and Earth's radiative forcing in the aerosol-climate model ECHAM5-HAM, Atmos. Chem. Phys., 10(22), 10733–10752, doi:10.5194/acp-10-10733-2010, 2010.
Keller, C. A., Long, M. S., Yantosca, R. M., Da Silva, A. M., Pawson, S. and Jacob, D. J.: HEMCO v1.0: a versatile, ESMF-compliant component for calculating emissions in atmospheric models, Geosci. Model Dev., 7(4), 1409–1417, doi:10.5194/gmd-7-1409-2014, 2014.
Kerkweg, A., Buchholz, J., Ganzeveld, L., Pozzer, A., Tost, H., and Jöckel, P.: Technical Note: An implementation of the dry removal processes DRY DEPosition and SEDImentation in the Modular Earth Submodel System (MESSy), Atmos. Chem. Phys., 6, 4617-4632, https://doi.org/10.5194/acp-6-4617-2006, 2006.
Kirkevåg, A., A. Grini, D. Olivié, Ø. Seland, K. Alterskjær, M. Hummel, I. H. H. Karset, A. Lewinschal, X. Liu, R.

Makkonen, I. Bethke, J. Griesfeller, M. Schulz, T. Iversen: A production-tagged aerosol module for Earth system models, OsloAero5.3 – extensions and updates for CAM5.3-Oslo, Geosci. Model Dev., 11, 3945-3982, https://doi.org/10.5194/gmd-11-3945-2018, 2018.

Klingmüller, K., Metzger, S., Abdelkader, M., Karydis, V. A., Stenchikov, G. L., Pozzer, A. and Lelieveld, J.: Revised mineral dust emissions in the atmospheric chemistry-climate model EMAC (based on MESSy 2.52), Geosci. Model Dev., 11, 989-1008, doi:10.5194/gmd-11-989-2018, 2018.

Koch, D., Jacob, D., Tegen, I., Rind, D., and Chin, M.: Tro- pospheric sulfur simulation and sulfate direct radiative forcing in the Goddard Institute for Space Studies general circulation model, J. Geophys. Res., 104, 23 799–23 822, 1999.

Koch, D., Schmidt, G., and Field, C.: Sulfur, sea salt and ra- dionuclide aerosols in the GISS modelE, J. Geophys. Res., 111, D06206, doi:10.1029/2004JD005550, 2006.

Kodros, J. K., Cucinotta, R., Ridley, D. A., Wiedinmyer, C., & Pierce, J. R. (2016). The aerosol radiative effects of uncontrolled combustion of domestic waste. Atmospheric Chemistry and Physics, 16(11), 6771–6784. http://doi.org/10.5194/acp-16-6771-2016

Kokkola, H., Hommel, R., Kazil, J., Niemeier, U., Partanen, A.-I., Feichter, J. and Timmreck, C.: Aerosol microphysics modules in the framework of the ECHAM5 climate model – intercomparison under stratospheric conditions, Geosci. Model Dev., 2(2), 97–112, doi:10.5194/gmd-2-97-2009, 2009.

Kulmala, M., Lehtinen, K. E. J. and Laaksonen, A.: Cluster activation theory as an explanation of the linear dependence between formation rate of 3nm particles and sulphuric acid concentration, Atmos. Chem. Phys., 6(3), 787–793, doi:10.5194/acp-6-787-2006, 2006.

Lamarque, J.-F., Emmons, L. K., Hess, P. G., Kinnison, D. E., Tilmes, S., Vitt, F., Heald, C. L., Holland, E. A., Lauritzen, P. H., Neu, J., Orlando, J. J., Rasch, P. J. and Tyndall, G. K.: CAM-chem: description and evaluation of interactive atmospheric chemistry in the Community Earth System Model, Geosci. Model Dev., 5(2), 369–411, doi:10.5194/gmd-5-369-2012, 2012.

Lamarque, J. F., Bond, T. C., Eyring, V., Granier, C., Heil, A., Klimont, Z., Lee, D., Liousse, C., Mieville, A., Owen, B., Schultz, M. G., Shindell, D., Smith, S. J., Stehfest, E., Van Aardenne, J., Cooper, O. R., Kainuma, M., Mahowald, N., McConnell, J. R., Naik, V., Riahi, K. and Van Vuuren, D. P.: Historical (1850-2000) gridded anthropogenic and biomass burning emissions of reactive gases and aerosols: Methodology and application, Atmos. Chem. Phys., 10(15), 7017–7039, doi:10.5194/acp-10-7017-2010, 2010.

Lamarque, J. F., Shindell, D. T., Josse, B., Young, P. J., Cionni, I., Eyring, V., Bergmann, D., Cameron-Smith, P., Collins, W. J., Doherty, R., Dalsoren, S., Faluvegi, G., Folberth, G., Ghan, S. J., Horowitz, L. W., Lee, Y. H., MacKenzie, I. A., Nagashima, T., Naik, V., Plummer, D., Righi, M., Rumbold, S. T., Schulz, M., Skeie, R. B., Stevenson, D. S., Strode, S., Sudo, K., Szopa, S., Voulgarakis, A. and Zeng, G.: The atmospheric chemistry and climate model intercomparison Project (ACCMIP): Overview and description of models, simulations and climate diagnostics, Geosci. Model Dev., 6(1), 179–206, doi:10.5194/gmd-6-179-2013, 2013.

Lee, Y. H. and Adams, P. J.: A Fast and Efficient Version of the TwO-Moment Aerosol Sectional (TOMAS) Global Aerosol Microphysics Model, Aerosol Sci. Technol., 46(6), 678–689, doi:10.1080/02786826.2011.643259, 2012.

Lee, Y. H., Adams, P. J. and Shindell, D. T.: Evaluation of the global aerosol microphysical ModelE2-TOMAS model against satellite and ground-based observations, Geosci. Model Dev., 8(3), 631–667, doi:10.5194/gmd-8-631-2015, 2015.

Liu, X., Easter, R. C. C., Ghan, S. J. J., Zaveri, R., Rasch, P., Shi, X., Lamarque, J.-F. F., Gettelman, A., Morrison, H., Vitt, F., Conley, A., Park, S., Neale, R., Hannay, C., Ekman, A. M. L. M. L., Hess, P., Mahowald, N., Collins, W., Iacono, M. J. J., Bretherton, C. S. S., Flanner, M. G. G. and Mitchell, D.: Toward a minimal representation of aerosols in climate models: Description and evaluation in the Community Atmosphere Model CAM5, Geosci. Model Dev., 5(3), 709–739, doi:10.5194/gmd-5-709-2012, 2012.

Liu, X., Ma, P.-L. L., Wang, H., Tilmes, S., Singh, B., Easter, R. C., Ghan, S. J. and Rasch, P. J.: Description and evaluation of a new four-mode version of the Modal Aerosol Module (MAM4) within version 5.3 of the Community Atmosphere Model, Geosci. Model Dev., 9(2), 505–522, doi:10.5194/gmd-9-505-2016, 2016.

Lohmann, U., & Neubauer, D.: The importance of mixed-phase and ice clouds for climate sensitivity in the global aerosol-climate model ECHAM6-HAM2. Atmospheric Chemistry and Physics, 18(12), 8807–8828. http://doi.org/10.5194/acp-18-8807-2018, 2018.

Long, M. S., Keene, W. C., Kieber, D. J., Erickson, D. J. and Maring, H.: A sea-state based source function for size- and

composition-resolved marine aerosol production, Atmos. Chem. Phys., 11(3), 1203–1216, doi:10.5194/acp-11-1203-2011, 2011.

Luo, G., and F. Yu, Representation of cloud droplet number concentration in the Community Atmosphere Model with Chemistry and the Advanced Particle Microphysics (CAM-Chem/APM), in preparation.

Mahowald, N. M., Muhs, D. R., Levis, S., Rasch, P. J., Yoshioka, M., Zender, C. S. and Luo, C.: Change in atmospheric mineral aerosols in response to climate: Last glacial period, preindustrial, modern, and doubled carbon dioxide climates, J. Geophys. Res. Atmos., 111(D10), n/a-n/a, doi:10.1029/2005JD006653, 2006.

Mann, G. W., Carslaw, K. S., Reddington, C. L., Pringle, K. J., Schulz, M., Asmi, A., Spracklen, D. V., Ridley, D. A., Woodhouse, M. T., Lee, L. A., Zhang, K., Ghan, S. J., Easter, R. C., Liu, X., Stier, P., Lee, Y. H., Adams, P. J., Tost, H., Lelieveld, J., Bauer, S. E., Tsigaridis, K., Van Noije, T. P. C., Strunk, A., Vignati, E., Bellouin, N., Dalvi, M., Johnson, C. E., Bergman, T., Kokkola, H., Von Salzen, K., Yu, F., Luo, G., Petzold, A., Heintzenberg, J., Clarke, A., Ogren, J. A., Gras, J., Baltensperger, U., Kaminski, U., Jennings, S. G., O'Dowd, C. D., Harrison, R. M., Beddows, D. C. S., Kulmala, M., Viisanen, Y., Ulevicius, V., Mihalopoulos, N., Zdimal, V., Fiebig, M., Hansson, H. C., Swietlicki, E. and Henzing, J. S.: Intercomparison and evaluation of global aerosol microphysical properties among AeroCom models of a range of complexity, Atmos. Chem. Phys., 14(9), 4679–4713, doi:10.5194/acp-14-4679-2014, 2014.

Mårtensson, E. M., Nilsson, E. D., de Leeuw, G., Cohen, L. H. and Hansson, H.-C.: Laboratory simulations and parameterization of the primary marine aerosol production, J. Geophys. Res. Atmos., 108(D9), n/a-n/a, doi:10.1029/2002JD002263, 2003.

Matsui, H., Koike, M., Kondo, Y., Takami, A., Fast, J. D., Kanaya, Y., & Takigawa, M.: Volatility basis-set approach simulation of organic aerosol formation in East Asia: Implications for anthropogenic-biogenic interaction and controllable amounts. Atmospheric Chemistry and Physics, 14(18), 9513–9535. http://doi.org/10.5194/acp-14-9513-2014, 2014.

Matsui, H.: Development of a global aerosol model using a two-dimensional sectional method : 1 . Model design, J. Adv. Model. Earth Syst., 9, 1921–1947, doi:10.1002/2017MS000936, 2017.

Matsui, H. and Mahowald, N.: Development of a global aerosol model using a two-dimensional sectional method: 2. Evaluation and sensitivity simulations, J. Adv. Model. Earth Syst., 9(4), 1887–1920, doi:10.1002/2017MS000937, 2017.

Mihalopoulos, N., Kerminen, V. M., Kanakidou, M., Berresheim, H. and Sciare, J.: Formation of particulate sulfur species (sulphate and methanesulfonate) during summer over the Eastern Mediterranean: A modelling approach, Atmos. Environ., 41(32), 6860–6871, doi:10.1016/j.atmosenv.2007.04.039, 2007.

Monahan, E. C., Spiel, D. E. and Davidson, K. L.: A Model of Marine Aerosol Generation Via Whitecaps and Wave Disruption, pp. 167–174, Springer Netherlands., 1986.

Myriokefalitakis, S., Tsigaridis, K., Mihalopoulos, N., Sciare, J., Nenes, A., Kawamura, K., Segers, A. and Kanakidou, M.: In-cloud oxalate formation in the global troposphere: A 3-D modeling study. *Atmos. Chem. Phys.*, 11, 5761-5782, doi:10.5194/acp-11-5761, 2011.

Myriokefalitakis, S., Daskalakis, N., Mihalopoulos, N., Baker, A. R., Nenes, A., and Kanakidou, M.: Changes in dissolved iron deposition to the oceans driven by human activity: a 3-D global modelling study, Biogeosciences, 12, 3973-3992, https://doi.org/10.5194/bg-12-3973-2015, 2015.

Napari, I., Kulmala, M. and Vehkamäki, H.: Ternary nucleation of inorganic acids, ammonia, and water, J. Chem. Phys., 117(18), 8418–8425, doi:10.1063/1.1511722, 2002.

Neale, R. B., Gettelman, A., Park, S., Chen, C.-C., Lauritzen, P. H., Williamson, D. L., Conley, A. J., Kinnison, D., Marsh, D., Smith, A. K., Vitt, F., Garcia, R., Lamarque, J.-F., Mills, M., Tilmes, S., Morrison, H., Cameron-smith, P., Collins, W. D., Iacono, M. J., Easter, R. C., Liu, X., Ghan, S. J., Rasch, P. J. and Taylor, M. A.: Description of the NCAR Community Atmosphere Model (CAM 5.0). NCAR Technical Notes., NCAR/Tn-464+Str, 214, doi:10.5065/D6N877R0., 2012.

Nenes, A. and Seinfeld, J. H.: Parameterization of cloud droplet formation in global climate models, J. Geophys. Res. Atmos., 108(D14), 4415, doi:10.1029/2002JD002911, 2003.

Neu, J. L. and Prather, M. J.: Toward a more physical representation of precipitation scavenging in global chemistry models: cloud overlap and ice physics and their impact on tropospheric ozone, Atmos. Chem. Phys., 12, 3289-3310, doi:10.5194/acp-12-3289-2012, 2012.

Neubauer, D., Lohmann, U., Hoose, C. and Frontoso, M. G.: Impact of the representation of marine stratocumulus clouds on

the anthropogenic aerosol effect, Atmos. Chem. Phys., 14(21), 11997–12022, doi:10.5194/acp-14-11997-2014, 2014.
Neubauer, D., Ferrachat, S., Siegenthaler-Le Drian, C., Stier, P., Partridge, P. D., Tegen, I., Bey, I., Stanelle, T., Kokkola, H., and Lohmann, U.: Cloud evaluation, aerosol radiative forcing and climate sensitivity in the global aerosol-climate model ECHAM6.3-HAM2.3, submitted to ACPD 2018.
Paasonen, P., Nieminen, T., Asmi, E., Manninen, H. E., Petäjä, T., Plass-Dülmer, C., Flentje, H., Birmili, W., Wiedensohler, A., Hõrrak, U., Metzger, A., Hamed, A., Laaksonen, A., Facchini, M. C., Kerminen, V.-M. and Kulmala, M.: On the roles of sulphuric acid and low-volatility organic vapours in the initial steps of atmospheric new particle formation, Atmos. Chem. Phys., 10(22), 11223–11242, doi:10.5194/acp-10-11223-2010, 2010.
Petters, M. D. and Kreidenweis, S. M.: A single parameter representation of hygroscopic growth and cloud\ncondensation nucleus activity, Atmos. Chem. Phys., 1961–1971, doi:10.5194/acp-7-1961-2007, 2007.
Petters, M. D., Kreidenweis, S. M., Sandu, A., Verwer, J. G., van Loon, M., Carmichael, G. R., Potra, F. A., Dabdub, D. and Seinfeld, J. H.: A single parameter representation of hygroscopic growth and cloud condensation nucleus activity, Atmos. Chem. Phys., 7(8), 1961–1971, doi:10.5194/acp-7-1961-2007, 2007.
Pringle, K. J., Tost, H., Message, S., Steil, B., Giannadaki, D., Nenes, A., Fountoukis, C., Stier, P., Vignati, E. and Lelieveld, J.: Description and evaluation of GMXe: A new aerosol submodel for global simulations (v1), Geosci. Model Dev., 3(2), 391–412, doi:10.5194/gmd-3-391-2010, 2010a.
Pringle, K. J., Tost, H., Pozzer, A., Pöschl, U. and Lelieveld, J.: Global distribution of the effective aerosol hygroscopicity parameter for CCN activation, Atmos. Chem. Phys., 10(12), 5241–5255, doi:10.5194/acp-10-5241-2010, 2010b.
Rasch, P. J., Feichter, J., and Law, K.: A comparison of scavenging and deposition processes in global models: results from the WCRP Cambridge Workshop of 1995, Tellus, 52B, 1025–1056, 2000.
Riahi, K., Grübler, A. and Nakicenovic, N.: Scenarios of long-term socio-economic and environmental development under climate stabilization, Technol. Forecast. Soc. Change, 74(7), 887–935, doi:10.1016/j.techfore.2006.05.026, 2007.
Riccobono, F., Schobesberger, S., Scott, C. E., Dommen, J., Ortega, I. K., Rondo, L., Almeida, J., Amorim, A., Bianchi, F., Breitenlechner, M., David, A., Downard, A., Dunne, E. M., Duplissy, J., Ehrhart, S., Flagan, R. C., Franchin, A., Hansel, A., Junninen, H., Kajos, M., Keskinen, H., Kupc, A., Kürten, A., Kvashin, A. N., Laaksonen, A., Lehtipalo, K., Makhmutov, V., Mathot, S., Nieminen, T., Onnela, A., Petäjä, T., Praplan, A. P., Santos, F. D., Schallhart, S., Seinfeld, J. H., Sipilä, M., Spracklen, D. V., Stozhkov, Y., Stratmann, F., Tomé, A., Tsagkogeorgas, G., Vaattovaara, P., Viisanen, Y., Vrtala, A., Wagner, P. E., Weingartner, E., Wex, H., Wimmer, D., Carslaw, K. S., Curtius, J., Donahue, N. M., Kirkby, J., Kulmala, M., Worsnop, D. R. and Baltensperger, U.: Oxidation products of biogenic emissions contribute to nucleation of atmospheric particles, Science., 344, 717–721, doi:10.1126/science.1243527, 2014a.
Roelofs, G.-J. and Lelieveld, J.: Distribution and budget of O3 in the troposphere calculated with a chemistry general circulation model, J. Geophys. Res., 100, 20983–20998, 1995.
Salisbury, D. J., Anguelova, M. D., and Brooks, I. M.: On the vari-ability of whitecap fraction using satellite-basedobservations, J. Geophys. Res., 118, 6201–6222, doi:10.1002/2013JC008797, 2013
Salter, M. E., Zieger, P., Acosta Navarro, J. C., Grythe, H., Kirkevåg, A., Rosati, B., Riipinen, I., and Nilsson, E. D.: An empirically derived inorganic sea spray source function incorpo- rating sea surface temperature, Atmos. Chem. Phys., 15, 11047–11066, doi:10.5194/acp-15-11047-2015, 2015.
Scanza, R. A., Mahowald, N., Ghan, S., Zender, C. S., Kok, J. F., Liu, X., Zhang, Y. and Albani, S.: Modeling dust as component minerals in the Community Atmosphere Model: development of framework and impact on radiative forcing, Atmos. Chem. Phys., 15(1), 537–561, doi:10.5194/acp-15-537-2015, 2015.
Schmale, J., Henning, S., Henzing, B., Keskinen, H., Sellegri, K., Ovadnevaite, J., Schlag, P.: Data Descriptor : Collocated observations of cloud condensation nuclei , particle size distributions , and chemical composition, 1–26, 2017.
Schmale, J., Henning, S., Decesari, S., Henzing, B., Keskinen, H., Sellegri, K., Gysel, M.: Long-term cloud condensation nuclei number concentration, particle number size distribution and chemical composition measurements at regionally representative observatories. Atmospheric Chemistry and Physics, 18(4), 2853–2881. http://doi.org/10.5194/acp-18-2853-2018, 2018.
Schmidt, G. A., Kelley, M., Nazarenko, L., Ruedy, R., Russell, G. L., Aleinov, I., Bauer, M., Bauer, S. E., Bhat, M. K., Bleck, R., Canuto, V., Chen, Y.-H., Cheng, Y., Clune, T. L., Del Genio, A., de Fainchtein, R., Faluvegi, G., Hansen, J. E., Healy, R. J., Kiang, N. Y., Koch, D., Lacis, A. A., LeGrande, A. N., Lerner, J., Lo, K. K., Matthews, E. E., Menon, S., Miller, R. L., Oinas, V., Oloso, A. O., Perlwitz, J. P., Puma, M. J., Putman, W. M., Rind, D., Romanou, A., Sato, M.,

Shindell, D. T., Sun, S., Syed, R. A., Tausnev, N., Tsigaridis, K., Unger, N., Voulgarakis, A., Yao, M.-S. and Zhang, J.: Configuration and assessment of the GISS ModelE2 contributions to the CMIP5 archive, J. Adv. Model. Earth Syst., 6(1), 141–184, doi:10.1002/2013MS000265, 2014.

Seinfeld, J. H. and Pandis, S. N.: Atmospheric Chemistry and Physics: From Air Pollution to Climate Change, 1st edition, J. Wiley, New York. 1998.

Seinfeld, J. and Pandis, S.: Atmospheric Chemistry and Physics: From Air Pollution to Climate Change., J Wiley, New York, 2006.

Seland, Ø., Iversen, T., Kirkevåg, A., and Storelvmo, T.: Aerosol - climate interactions in the CAM-Oslo atmospheric GCM and investigations of associated shortcomings, Tellus A, 60, 459–491, 2008

Shindell, D. T., Faluvegi, G., Unger, N., Aguilar, E., Schmidt, G. A., Koch, D. M., Bauer, S. E., and Miller, R. L.: Simulations of preindustrial, present-day, and 2100 conditions in the NASA GISS composition and climate model G-PUCCINI, Atmos. Chem. Phys., 6, 4427-4459, https://doi.org/10.5194/acp-6-4427-2006, 2006.

Sindelarova, K., Granier, C., Bouarar, I., Guenther, A., Tilmes, S., Stavrakou, T., Müller, J.-F., Kuhn, U., Stefani, P., and Knorr, W.: Global data set of biogenic VOC emissions calculated by the MEGAN model over the last 30 years, Atmos. Chem. Phys., 14, 9317–9341, http://doi.org/10.5194/acp-14-9317-2014, 2014.

Sofiev, M., Soares, J., Prank, M., De Leeuw, G. and Kukkonen, J.: A regional-to-global model of emission and transport of sea salt particles in the atmosphere, J. Geophys. Res. Atmos., 116(21), doi:10.1029/2010JD014713, 2011.

Stier, P., Feichter, J., Kinne, S., Kloster, S., Vignati, E., Wilson,J., Ganzeveld, L., Tegen, I., Werner, M., Schulz, M., Balkanski, Y., Boucher, O., Minikin, A., and Petzold, A.: The aerosol-climate model ECHAM5-HAM, Atmos. Chem. Phys., 5, 1125–1165, 2005.

Stevens, B., Giorgetta, M., Esch, M., Mauritsen, T., Crueger, T., Rast, S., Salzmann, M., Schmidt, H., Bader, J., Block, K., Brokopf, R., Fast, I., Kinne, S., Kornblueh, L., Lohmann, U., Pincus, R., Reichler, T. and Roeckner, E.: Atmospheric component of the MPI-M earth system model: ECHAM6, J. Adv. Model. Earth Syst., 5(2), 146–172, doi:10.1002/jame.20015, 2013.

Tegen, I., Harrison, S. P., Kohfeld, K., Prentice, I. C., Coe, M. and Heimann, M.: Impact of vegetation and preferential source areas on global dust aerosol: Results from a model study, J. Geophys. Res. Atmos., 107(21), doi:10.1029/2001JD000963, 2002.

Tegen, I., Neubauer, D., Ferrachat, S., Siegenthaler-Le Drian, C., Bey, I., Schutgens, N., Stier, P., Watson-Parris, D., Stanelle, T., Schmidt, H., Rast, S., Kokkola, H., Schultz, M., Schroeder, S., Daskalakis, N., Barthel, S., Heinold, B., and Lohmann, U.: The global aerosol–climate model ECHAM6.3–HAM2.3 – Part 1: Aerosol evaluation, Geosci. Model Dev., 12, 1643-1677, https://doi.org/10.5194/gmd-12-1643-2019, 2019.

Tost, H., Jöckel, P., Kerkweg, A., Sander, R., and Lelieveld, J.: Technical note: A new comprehensive SCAVenging submodel for global atmospheric chemistry modelling, Atmos. Chem. Phys., 6, 565-574, https://doi.org/10.5194/acp-6-565-2006, 2006.

Trivitayanurak, W., Adams, P. J., Spracklen, D. V., & Carslaw, K. S. : Tropospheric aerosol microphysics simulation with assimilated meteorology: Model description and intermodel comparison. Atmospheric Chemistry and Physics, 8(12), 3149–3168. http://doi.org/10.5194/acp-8-3149-2008, 2008.

Tsigaridis, K. and Kanakidou, M.: Global modelling of secondary organic aerosol in the troposphere: a sensitivity analysis, Atmos. Chem. Phys., 3(5), 1849–1869, doi:10.5194/acp-3-1849-2003, 2003.

Tsigaridis, K. and Kanakidou, M.: Secondary organic aerosol importance in the future atmosphere, Atmos. Environ., 41(22), 4682–4692, doi:http://dx.doi.org/10.1016/j.atmosenv.2007.03.045, 2007.

Tsigaridis, K., Krol, M., Dentener, F.J., Balkanski, Y., Lathière, J., Metzger, S., Hauglustaine, D.A. and Kanakidou, M.: Change in global aerosol composition since preindustrial times. Atmos. Chem. Phys., 6, 5143-5162, doi:10.5194/acp-6-5143, 2006.

Tsigaridis, K., Daskalakis, N., Kanakidou, M., Adams, P. J., Artaxo, P., Bahadur, R., Balkanski, Y., Bauer, S. E., Bellouin, N., Benedetti, A., Bergman, T., Berntsen, T. K., Beukes, J. P., Bian, H., Carslaw, K. S., Chin, M., Curci, G., Diehl, T., Easter, R. C., Ghan, S. J., Gong, S. L., Hodzic, A., Hoyle, C. R., Iversen, T., Jathar, S., Jimenez, J. L., Kaiser, J. W., Kirkevåg, A., Koch, D., Kokkola, H., Lee, Y. H., Lin, G., Liu, X., Luo, G., Ma, X., Mann, G. W., Mihalopoulos, N., Morcrette, J.-J., Müller, J.-F., Myhre, G., Myriokefalitakis, S., Ng, N. L., O'Donnell, D., Penner, J. E., Pozzoli, L., Pringle, K. J., Russell, L. M., Schulz, M., Sciare, J., Seland, Ø., Shindell, D. T., Sillman, S., Skeie, R. B., Spracklen, D.,

Stavrakou, T., Steenrod, S. D., Takemura, T., Tiitta, P., Tilmes, S., Tost, H., van Noije, T., van Zyl, P. G., von Salzen, K., Yu, F., Wang, Z., Wang, Z., Zaveri, R. A., Zhang, H., Zhang, K., Zhang, Q. and Zhang, X.: The AeroCom evaluation and intercomparison of organic aerosol in global models, Atmos. Chem. Phys., 14(19), 10845–10895, doi:10.5194/acp-14-10845-2014, 2014.

Van Marle, M. J. E., Kloster, S., Magi, B. I., Marlon, J. R., Daniau, A.-L., Field, R. D., Arneth, A., Forrest, M., Hantson, S., Kehrwald, N. M., Knorr, W., Lasslop, G., Li, F., Mangeon, S., Yue, C., Kaiser, J. W. and van der Werf, G. R.: Historic global biomass burning emissions based on merging satellite observations with proxies and fire models (1750–2015), Geosci. Model Dev., 3329-3357, doi:10.5194/gmd-10-3329-2017, 2017.

Val Martin, M., Logan, J. A., Kahn, R. A., Leung, F.-Y., Nelson, D. L., and Diner, D. J.: Smoke injection heights from fires in North America: analysis of 5 years of satellite observations, Atmospheric Chemistry and Physics, 10, 1491–1510, 2010.

Van Noije, T. P. C., Le Sager, P., Segers, A. J., Van Velthoven, P. F. J., Krol, M. C., Hazeleger, W., Williams, A. G. and Chambers, S. D.: Simulation of tropospheric chemistry and aerosols with the climate model EC-Earth, Geosci. Model Dev., 7, 2435–2475, doi:10.5194/gmd-7-2435-2014, 2014.

Vehkamäki, H.: An improved parameterization for sulfuric acid–water nucleation rates for tropospheric and stratospheric conditions, J. Geophys. Res., 107(D22), 4622, doi:10.1029/2002JD002184, 2002.

Vignati, E., Wilson, J. and Stier, P.: M7: An efficient size-resolved aerosol microphysics module for large-scale aerosol transport models, J. Geophys. Res. Atmos., 109(D22), n/a-n/a, doi:10.1029/2003JD004485, 2004.

Vignati, E., Facchini, M. C., Rinaldi, M., Scannell, C., Ceburnis, D., Sciare, J., Kanakidou, M., Myriokefalitakis, S., Dentener, F. and O'Dowd, C. D.: Global scale emission and distribution of sea-spray aerosol: Sea-salt and organic enrichment, Atmos. Environ., 44(5), 670–677, doi:10.1016/j.atmosenv.2009.11.013, 2010.

Wang, Q., D.J. Jacob, J.A. Fisher, J. Mao, E.M. Leibensperger, C.C. Carouge, P. Le Sager, Y. Kondo, J.L. Jimenez, M.J. Cubison, and S.J. Doherty, Sources of carbonaceous aerosols and deposited black carbon in the Arctic in winter-spring: implications for radiative forcing, Atmos. Chem. Phys., 11, 12,453-12,473, 2011.

Wang, H., Easter, R. C., Rasch, P. J., Wang, M., Liu, X., Ghan, S. J., Qian, Y., Yoon, J.-H., Ma, P.-L. and Vinoj, V.: Sensitivity of remote aerosol distributions to representation of cloud–aerosol interactions in a global climate model, Geosci. Model Dev., 6(3), 765–782, doi:10.5194/gmd-6-765-2013, 2013.

Wiedinmyer, C., Akagi, S. K., Yokelson, R. J., Emmons, L. K., Al-Saadi, J. A., Orlando, J. J., & Soja, A. J.: The Fire INventory from NCAR (FINN): a high resolution global model to estimate the emissions from open burning. Geoscientific Model Development, 4(3), 625–641. https://doi.org/10.5194/gmd-4-625-2011, 2011.

Wesely, M. L.: Parameterization of surface resistances to gaseous dry deposition in regional-scale numerical models, Atmos. Env-iron., 23, 1293–1304, 1989

Westervelt, D. M., Pierce, J. R., Riipinen, I., Trivitayanurak, W., Hamed, A., Kulmala, M., Laaksonen, A., Decesari, S., and Adams, P. J.: Formation and growth of nucleated particles into cloud condensation nuclei: model-measurement comparison, Atmospheric Chemistry and Physics, 13, 7645-7663, doi:10.5194/acp-13-7645-2013, 2013.

Yang, Y., Wang, H., Smith, S. J., Ma, P.-L. and Rasch, P. J.: Source attribution of black carbon and its direct radiative forcing in China, Atmos. Chem. Phys., 17(6), 4319–4336, doi:10.5194/acp-17-4319-2017, 2017.

Yu, F.: A secondary organic aerosol formation model considering successive oxidation aging and kinetic condensation of organic compounds: Global scale implications, Atmos. Chem. Phys., 11(3), 1083–1099, doi:10.5194/acp-11-1083-2011, 2011.

Yu, F. and Luo, G.: Simulation of particle size distribution with a global aerosol model: contribution of nucleation to aerosol and CCN number concentrations, Atmos. Chem. Phys. 9, 7691-7710, https://doi.org/10.5194/acp-9-7691-2009, 2009.

Yu, F., Nadykto, A. B., Herb, J., Luo, G., Nazarenko, K. M., and Uvarova, L. A.: H2SO4-H2O-NH3 ternary ion-mediated nucleation (TIMN): Kinetic-based model and comparison with CLOUD measurements, Atmos. Chem. Phys. Discuss., https://doi.org/10.5194/acp-2018-396, in review, 2018.

Zender, C. S., Bian, H. and Newman, D.: Mineral Dust Entrainment and Deposition (DEAD) model: Description and 1990s dust climatology, J. Geophys. Res., 108(D14), 4416, doi:10.1029/2002JD002775, 2003.

Zhang, L. M., Gong, S. L., Padro, J., and Barrie, L.: A size-segregated particle dry deposition scheme for an atmospheric aerosol module, Atmos. Environ., 35(3), 549-560, doi:10.1016/s1352-2310(00)00326-5, 2001.

Zhang, K., O'Donnell, D., Kazil, J., Stier, P., Kinne, S., Lohmann, U., Ferrachat, S., Croft, B., Quaas, J., Wan, H., Rast, S.

and Feichter, J.: The global aerosol-climate model ECHAM-HAM, version 2: Sensitivity to improvements in process representations, Atmos. Chem. Phys., 12(19), 8911–8949, doi:10.5194/acp-12-8911-2012, 2012.

Supplementary figures captions

The following figures can be found in a separate file:

Fig. S1. Monthly averages of the mass concentration of sulphate (SO_4), organics (OA), dust (DU) and sea-salt (SS) of PM_1 particles for the period 2011-2015. The median of all models is shown with blue bold line, while the shaded areas depict the 25%/75 % quartiles of all data. The green-dashed lines show the min/max values of all models. Available observational data are also shown with black dashed-lines and dots. Observations are from Schmale et al. (2017). Note that SS is here derived from Cl^- PM_1 measurements that underestimate the SS levels.

Fig. S2. Comparisons of the monthly mean observations of CCN at various supersaturation ratios, N_{50}, N_{80}, N_{120} particle number concentrations, and sulphate (SO_4) and organic aerosol (OA) mass concentrations with the corresponding results of each model. Models are plotted with different colours as indicated in the figure legend. Observations (black circles) are from Schmale et al. (2017).

Fig. S3. Comparison between monthly averages of the cloud droplet properties from observations (black circles) and from the individual models (see figure legend for model identification). Figures are drawn per station and for two different updraft velocities (w=0.3 ms^{-1} and w=0.6 ms^{-1}) marked on the y-axis. For each station and updraft velocity the five graphs show (as indicated in the *y*-axis label), the total number of particles Nt, the number of cloud droplets, N_d, the maximum supersaturation, s_{max} (in %), the sensitivity of the N_d to the total number of aerosol particles, $(\partial N_d/\partial N_a)$, and the sensitivity of the N_d to the wind speed, $(\partial N_d/\partial w)$.

Fig. S4. Comparison between the persistence times derived from the observations (in black bars) and from the model results of $CCN_{0.2}$ during winter and summer for each station. Each pair of bars that follows corresponds to the predictions of each model for winter (left bar) and summer (right shaded bar), respectively. The white bars show the persistence times of the MMM. The persistence times derived from model simulations have been computed at the same time periods as those derived from the observations.

Fig. S5. (a) Comparison of the winter and summer persistence times of $CCN_{0.2}$ for Finokalia station calculated using observational data (black bars) and the results of TM4-ECPL model for the base simulation (red bars) and the sensitivity simulation assuming the same emission of carbonaceous aerosols with the base case but fewer particles of larger size (green bars). The pairs of columns show persistences first for winter and second for summer. In both simulations a log-normal distribution

for the size distribution of emitted particles with a geometric standard deviation σ=1.59 has been assumed/used. In the base simulation, the dry median diameters of the emitted particles coming from fossil fuel combustion and from vegetation fires are 30 nm and 80 nm, respectively, while in the sensitivity simulations all particles are emitted at 60 nm. Left bars are for winter and right shaded bars are for summer. (b) Autocorrelation function (ACF) of the $CCN_{0.2}$ for Finokalia station calculated for summer (continuous lines) and for winter (dashed lines). Lines in colors are for the bars. The large-lag standard deviation curves are also shown in the graphs. The persistence time is defined as the time that the large-lag standard error crosses the ACF curve (Schmale et al., 2018). (c) same as panel-b using the results of the sensitivity simulation. (d)-(f) same as the panel-a for Cabauw, Hyytiälä and Mace Head stations, respectively.

Fig. S6. Global surface distribution for the year 2011 of the N_3 number concentrations as computed by the fifteen models that participated in this study. At the top of each panel the maximum value of the N_3 simulated by the model is denoted. Units are number of particles.cm^{-3}.

Fig. S7. Same as Figure-S6 for N_{50} number concentrations. Units are number of particles.cm^{-3}.

Fig. S8. Same as Figure-S6 for N_{120} number concentrations. Units are number of particles.cm^{-3}.

Fig. S9. Same as Figure-S6 for $CCN_{0.2}$ number concentrations. Units are number of particles.cm^{-3}.

Fig. S10. Global surface distribution of the mass concentration of sulphate (SO4) of PM_1 particles as computed by all models. At the top of each map the maximum value of the SO4 is denoted. Units are μg-SO4.m^{-3}

Fig. S11. Same as Figure-S10 for OA. Units are μg-OA.m^{-3}

Fig. S12. Same as Figure-S10 for BC. Units are μg.m^{-3}.

Fig. S13. Same as Figure-S10 for SS. Units are μg.m^{-3}

Fig. S14. Same as Figure-S10 for DU. Units are μg.m^{-3}

Fig. S15. Global distributions of the annual multi-model median concentrations of the SO4, OA, BC, DU and SS (from top to bottom) for the year 2011 (left column) and the corresponding diversities (right column). Model diversities are calculated as the ratio of the standard deviation to the mean of the models.

Fig. S16. Monthly ensembles for the years 2011-2015 of the CCN number concentration for supersaturation 0.2 % ($CCN_{0.2}$), 0.1% ($CCN_{0.1}$), 0.7% ($CCN_{0.7}$) and 1.0% ($CCN_{1.0}$) when observational data are available for Finokalia, Cabauw and Vavihill.

Fig. S17. Mean $CCN_{0.2}$ as calculated from the observations and as computed from the daily MMM for the days with available observations. The stations have been ranked based on CCN0.2 observations in decreasing levels.

Fig. S18. Ratio of the model diversity of N_3 (Figure 10b) to that of $CCN_{0.2}$ (Figure 10f).
