# Supplementary material for: Evaluation of global simulations of aerosol particle and cloud condensation nuclei number, with implications for cloud droplet formation
Source: Atmos Chem Phys. Author manuscript; Available in PMC 2020 Dec 2. (PMC7709872; doi:10.5194/acp-19-8591-2019)
Supplement: Supplementary figures [file NIHMS1643828-supplement-Supplementary_figures.pdf]

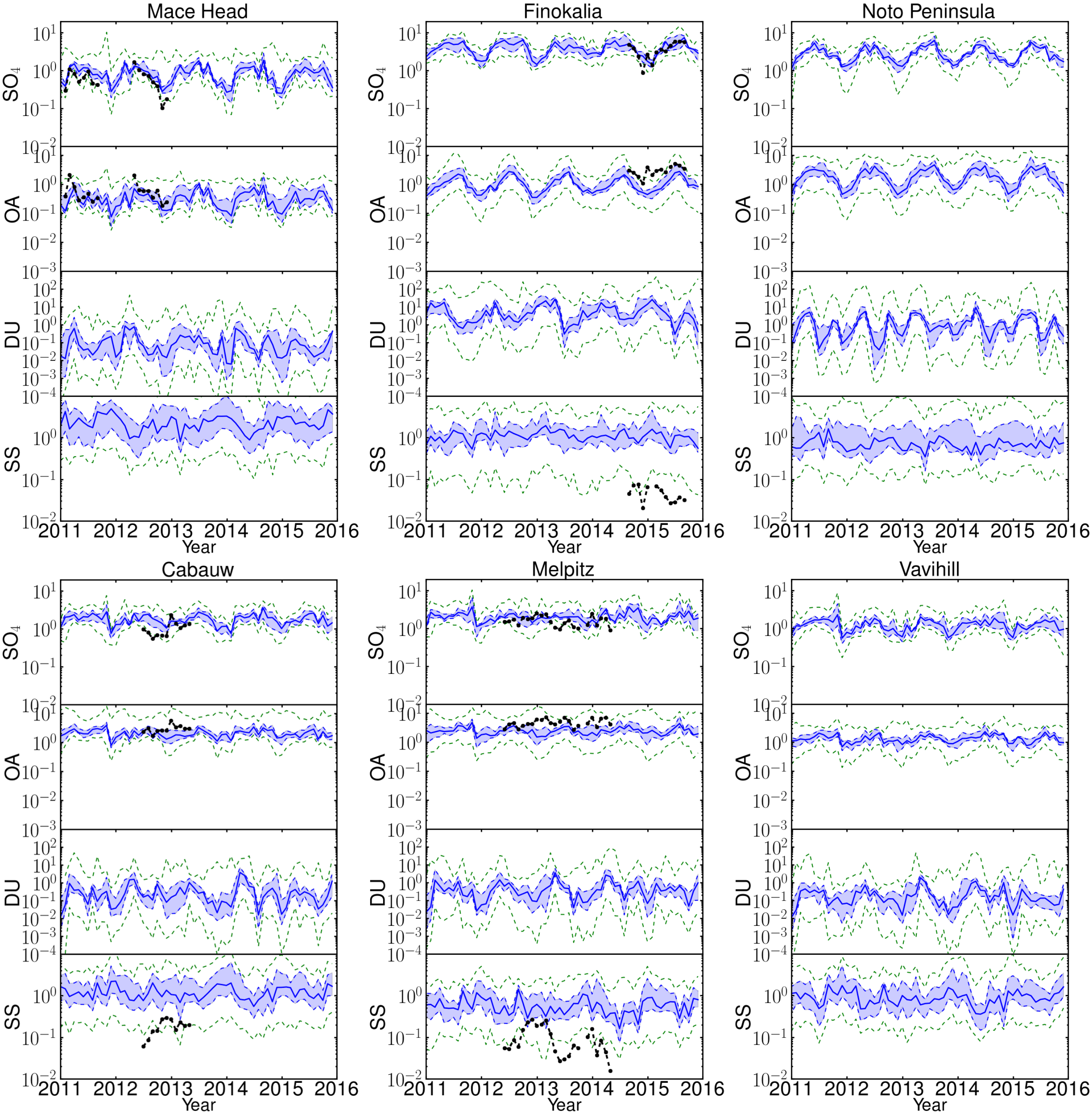

Figure-S1: Monthly averages of the mass concentration of sulfate (SO4), organics (OA), dust (DU) and sea-salt (SS) of PM_1 particles for the period 2011-2015. The median of all models is shown with blue bold line, while the shaded areas depict the 25 %/75 % quartiles of all data. The green-dashed lines show the min/max values of all models. Available observational data are also shown with black dashed-lines and dots. Observations are from Schmale et al. (2017). Note that SS is here derived from Cl^- PM_1 measurements that underestimate the SS levels.

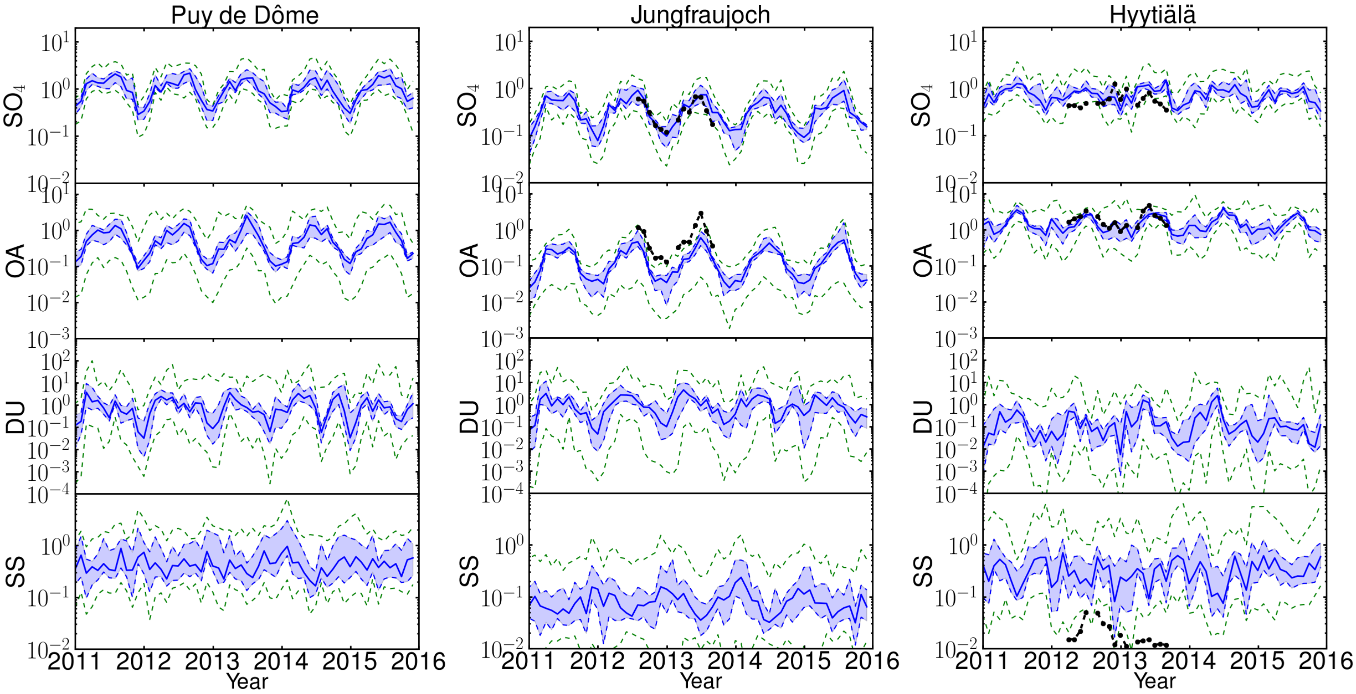

Figure-S1 (cont)

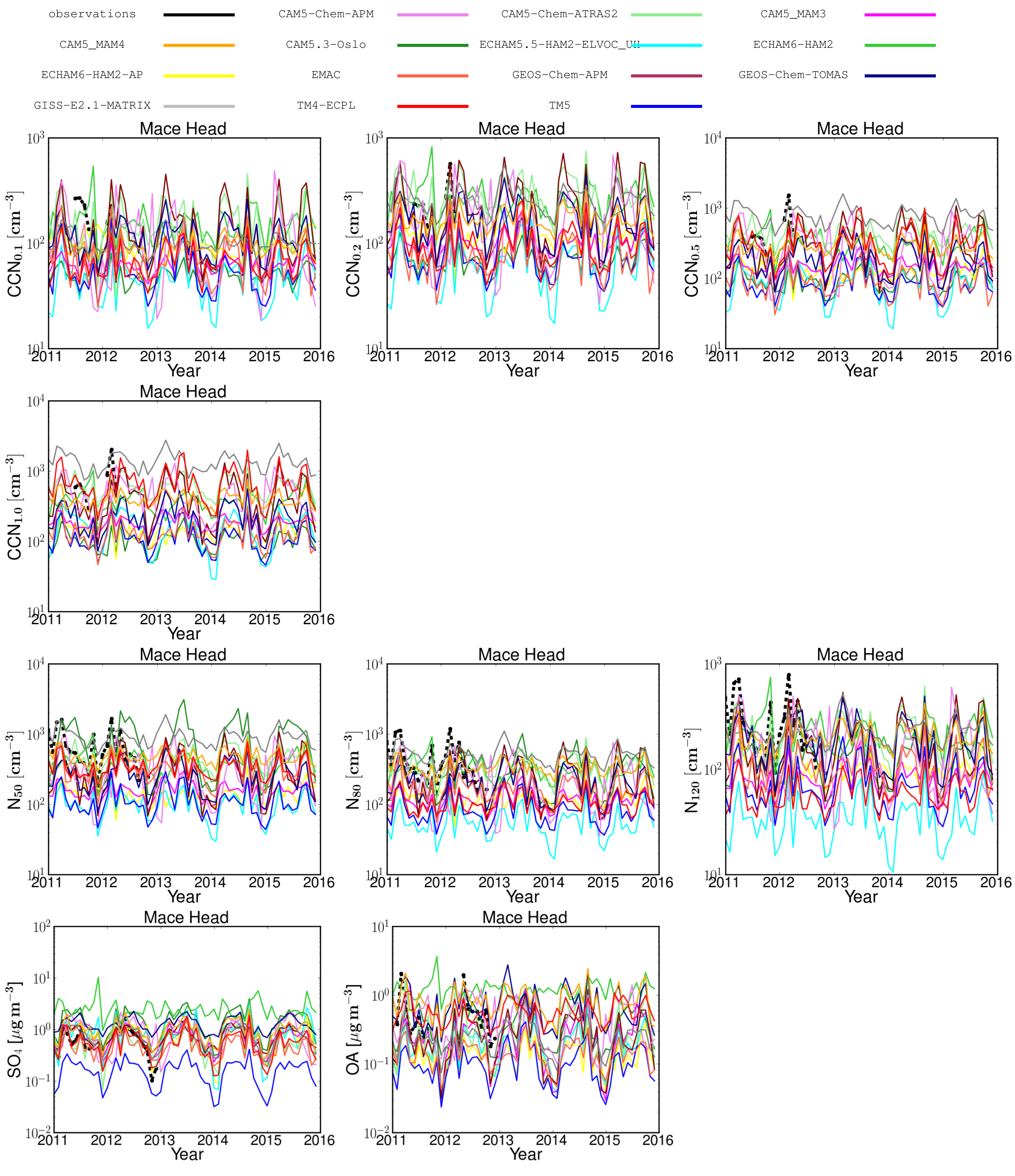

Figure-S2: Comparisons of the monthly mean observations of CCN at various supersaturation ratios, N_{50}, N_{80}, N_{120} particle number concentrations, and sulfate (SO4) and organic aerosol (OA) mass concentrations with the corresponding results of each model. Models are plotted with different colours as indicated in the figure legend. Observations (black circles) are from Schmale et al. (2017).

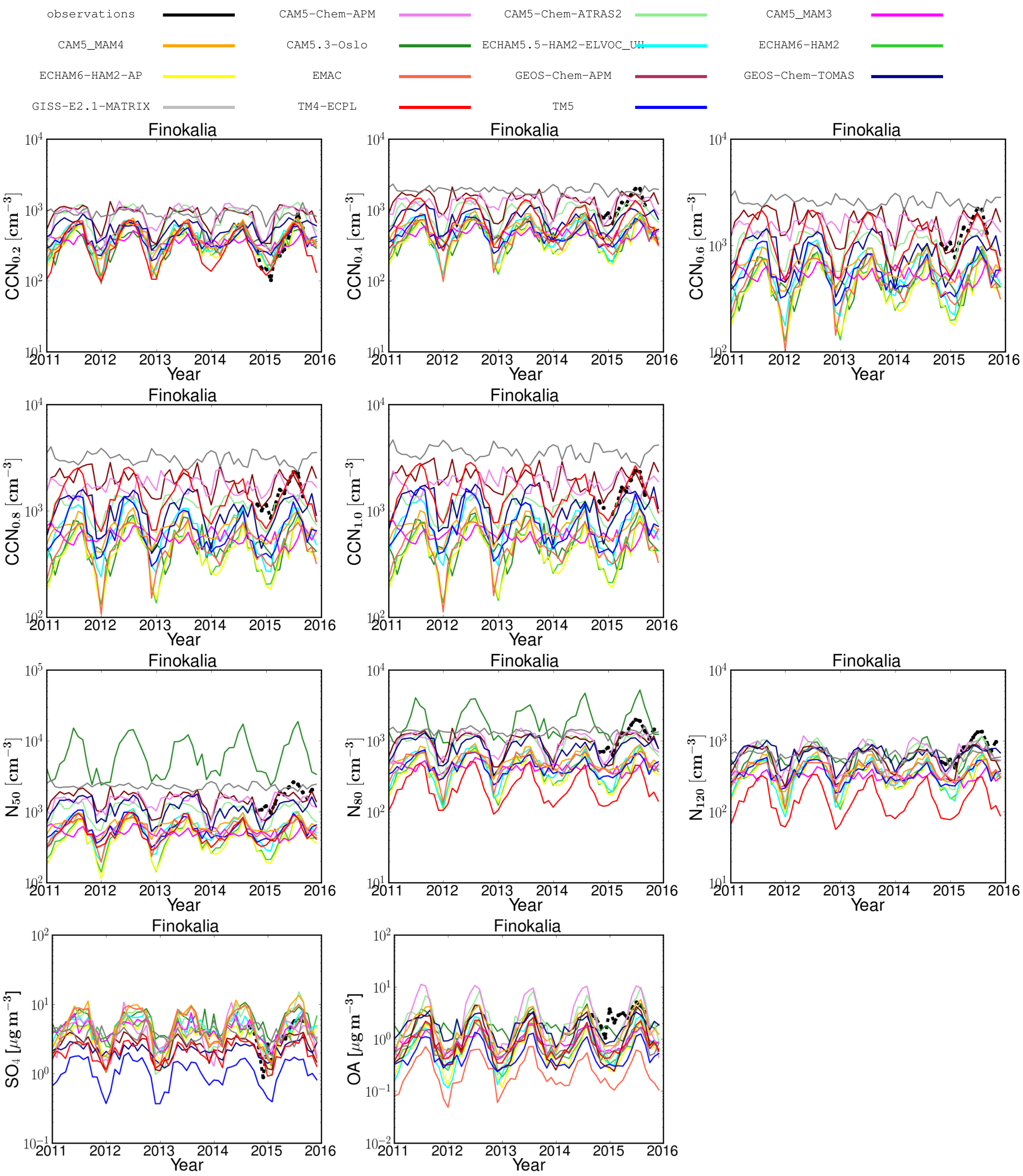

Figure S2 (cont 2/9)

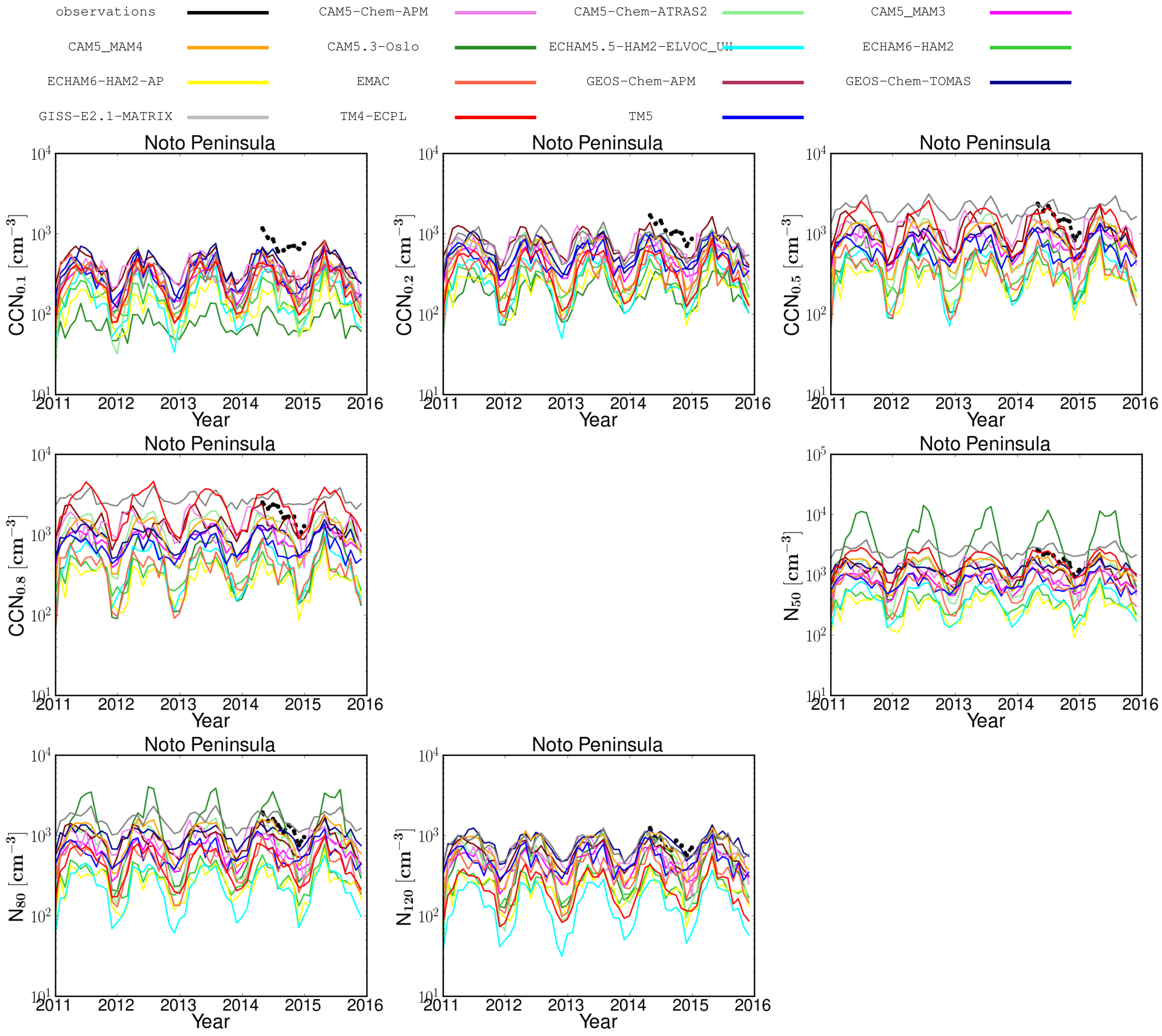

Figure S2 (cont 3/9)

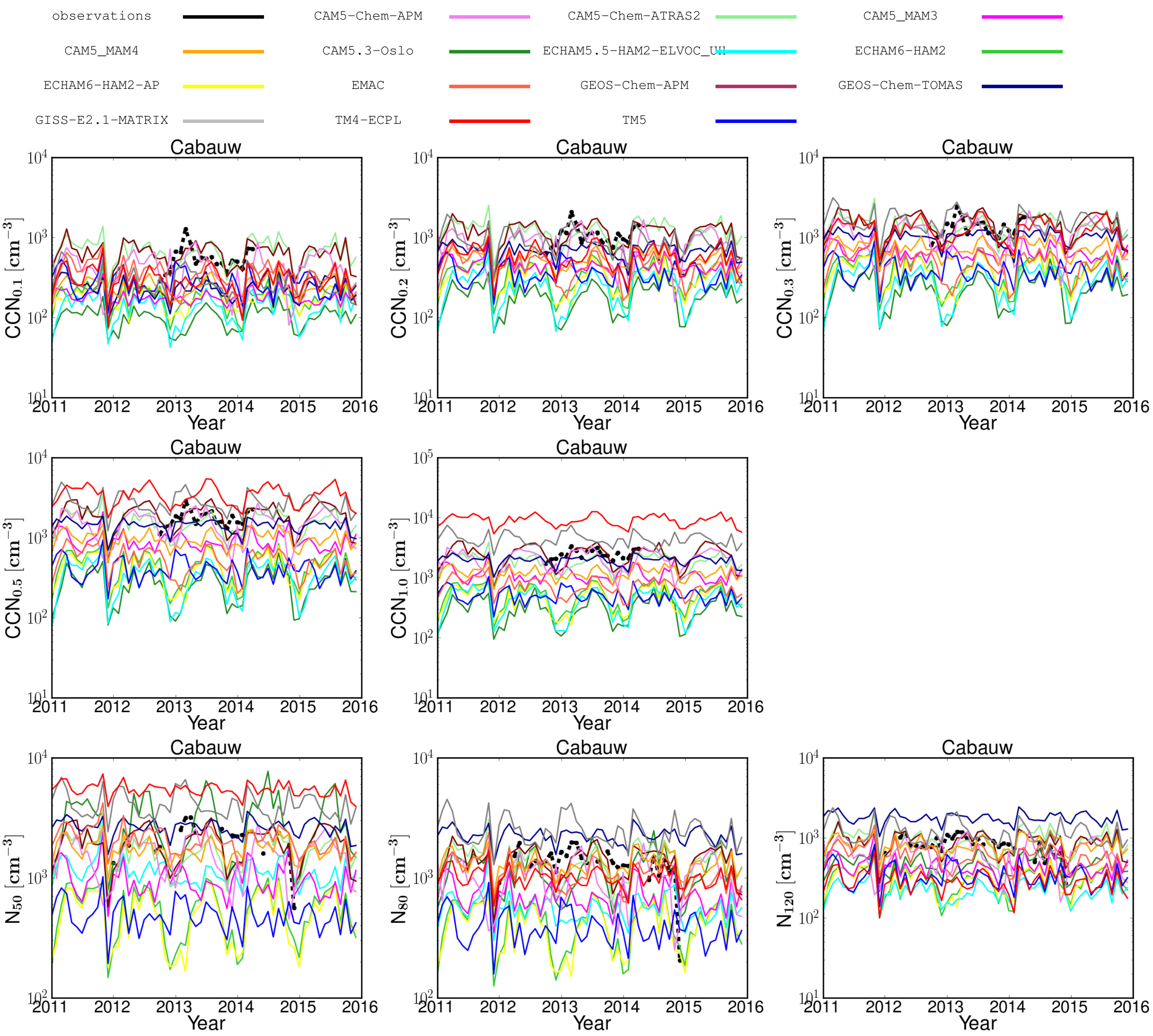

Figure S2 (cont 4/9)

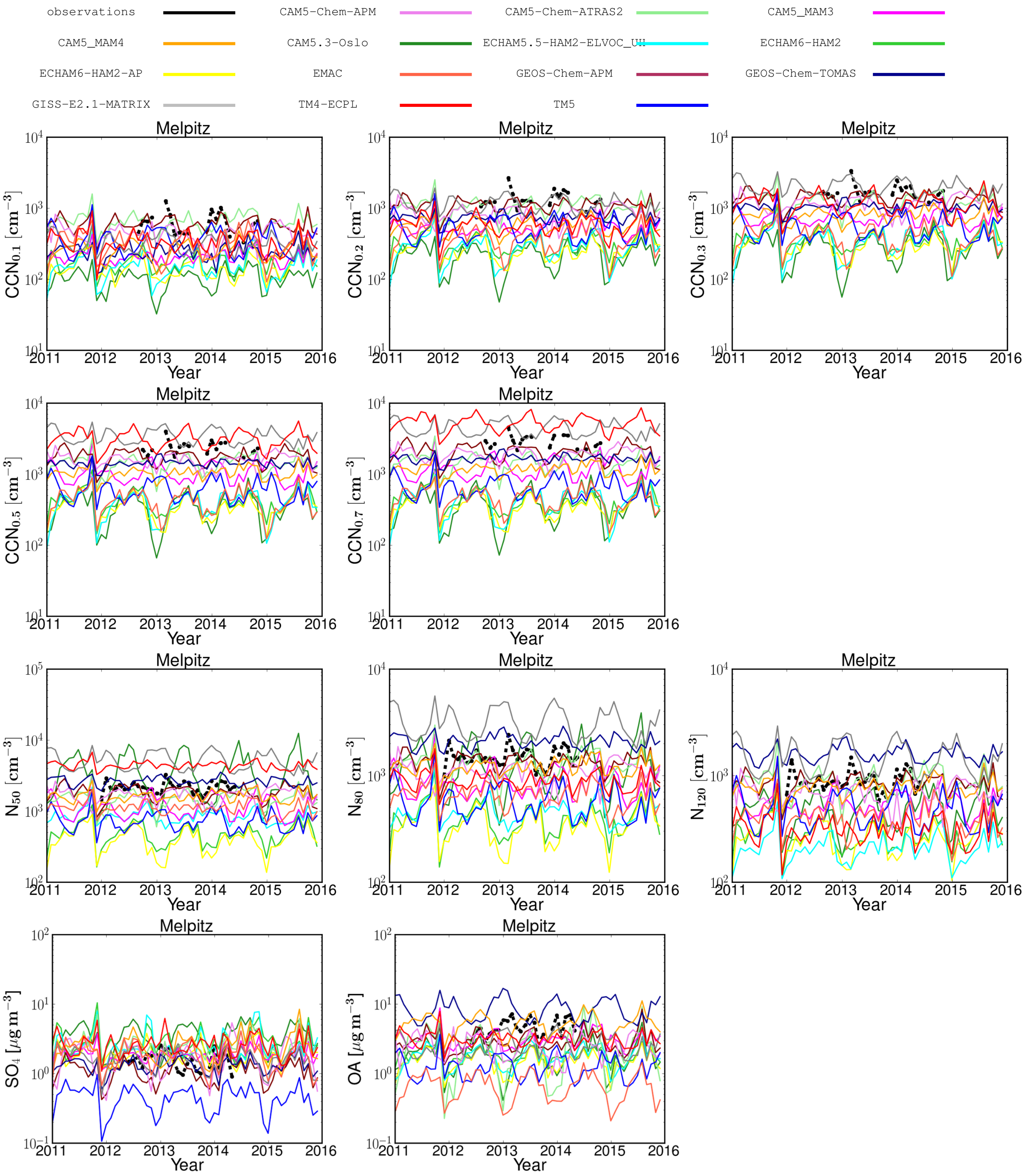

Figure S2 (cont 5/9)

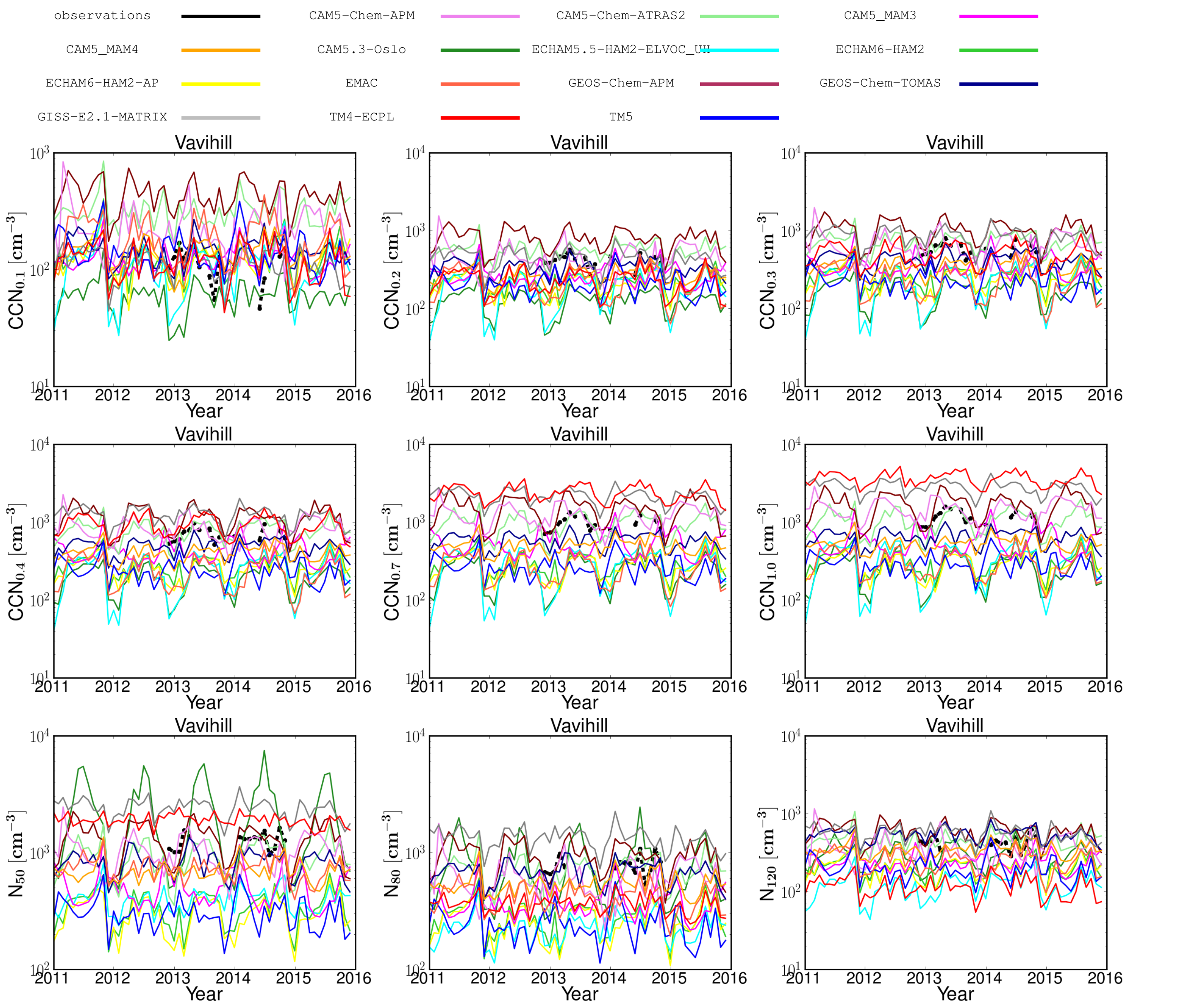

Figure S2 (cont 6/9)

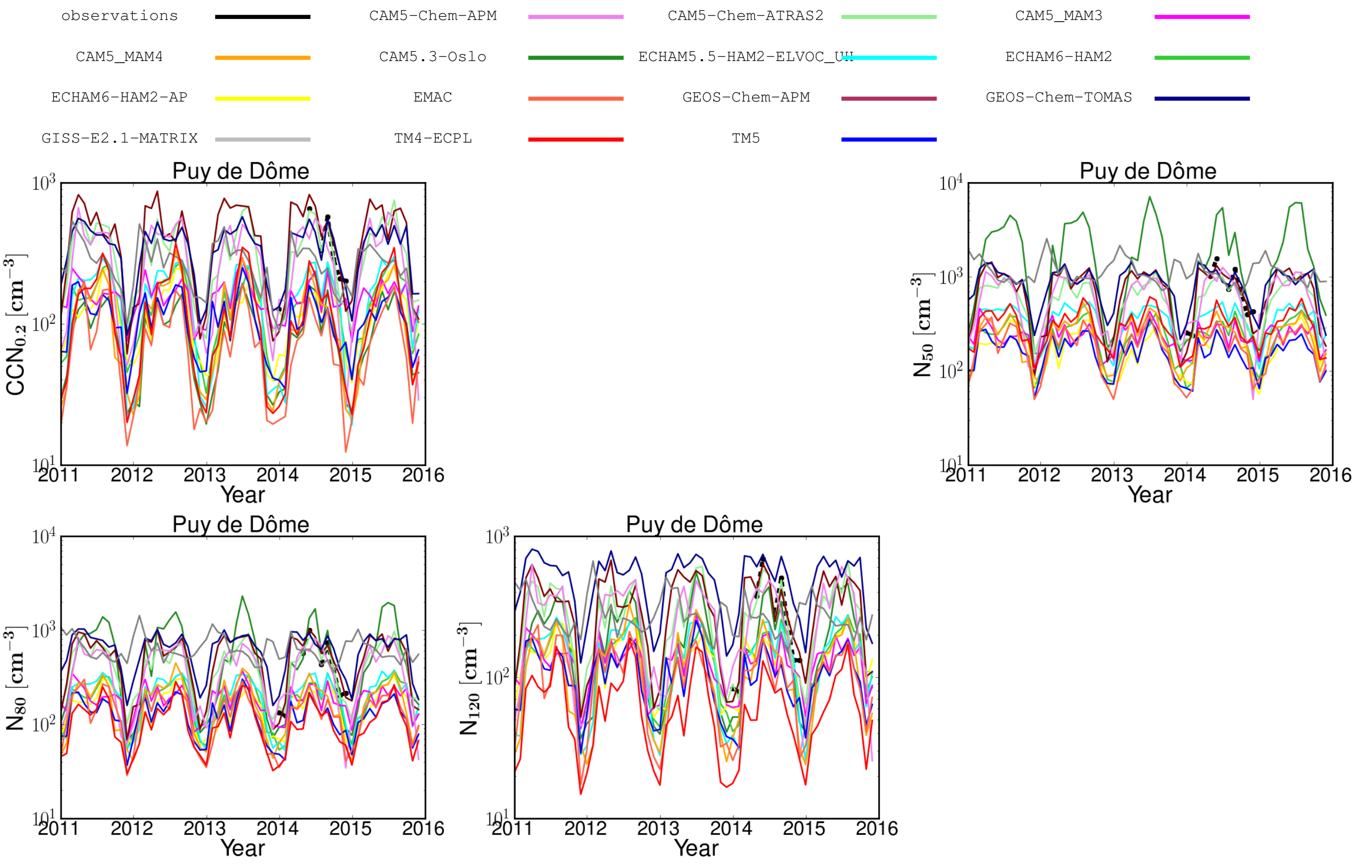

Figure S2 (cont 7/9)

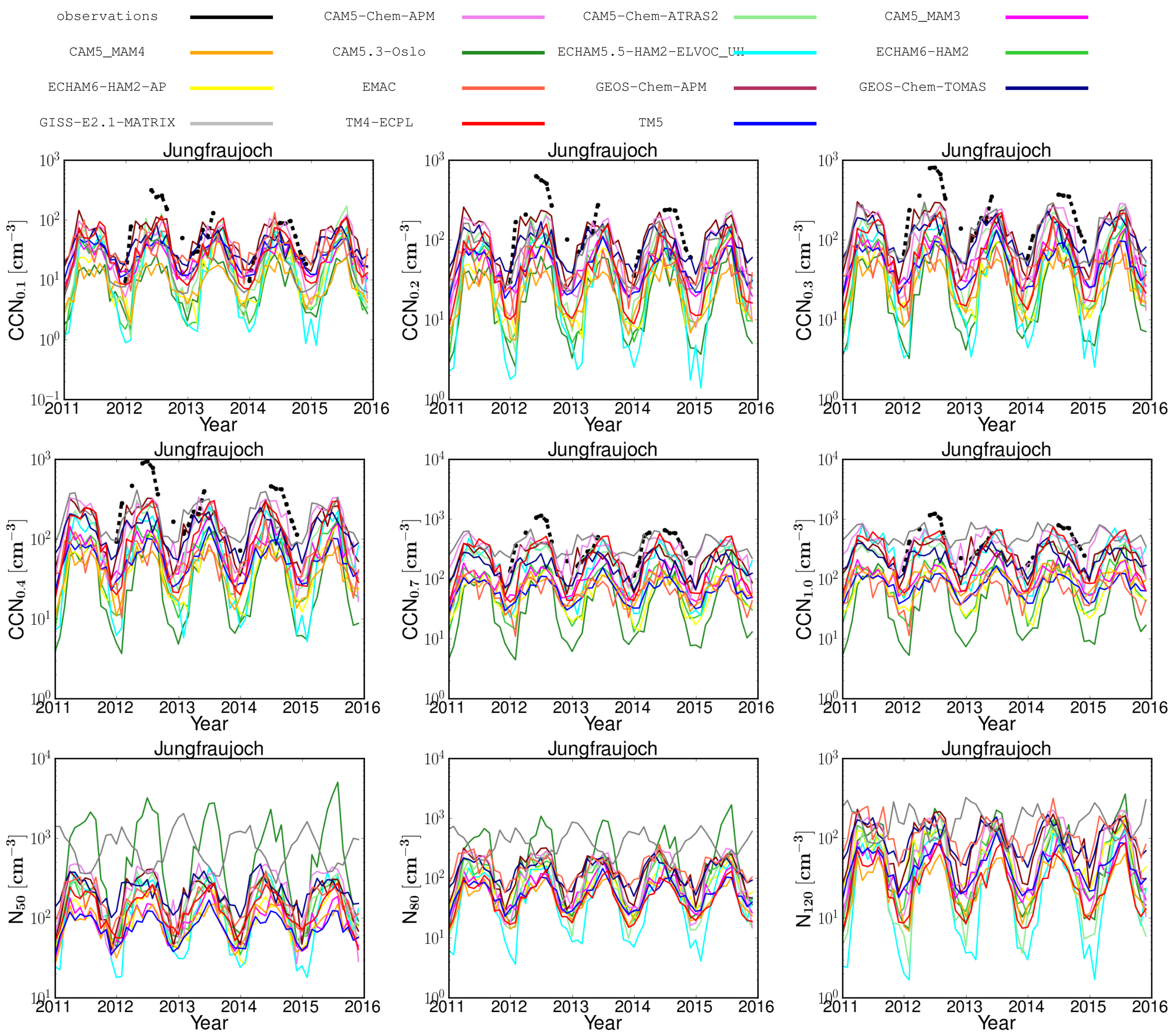

Figure S2 (cont 8/9)

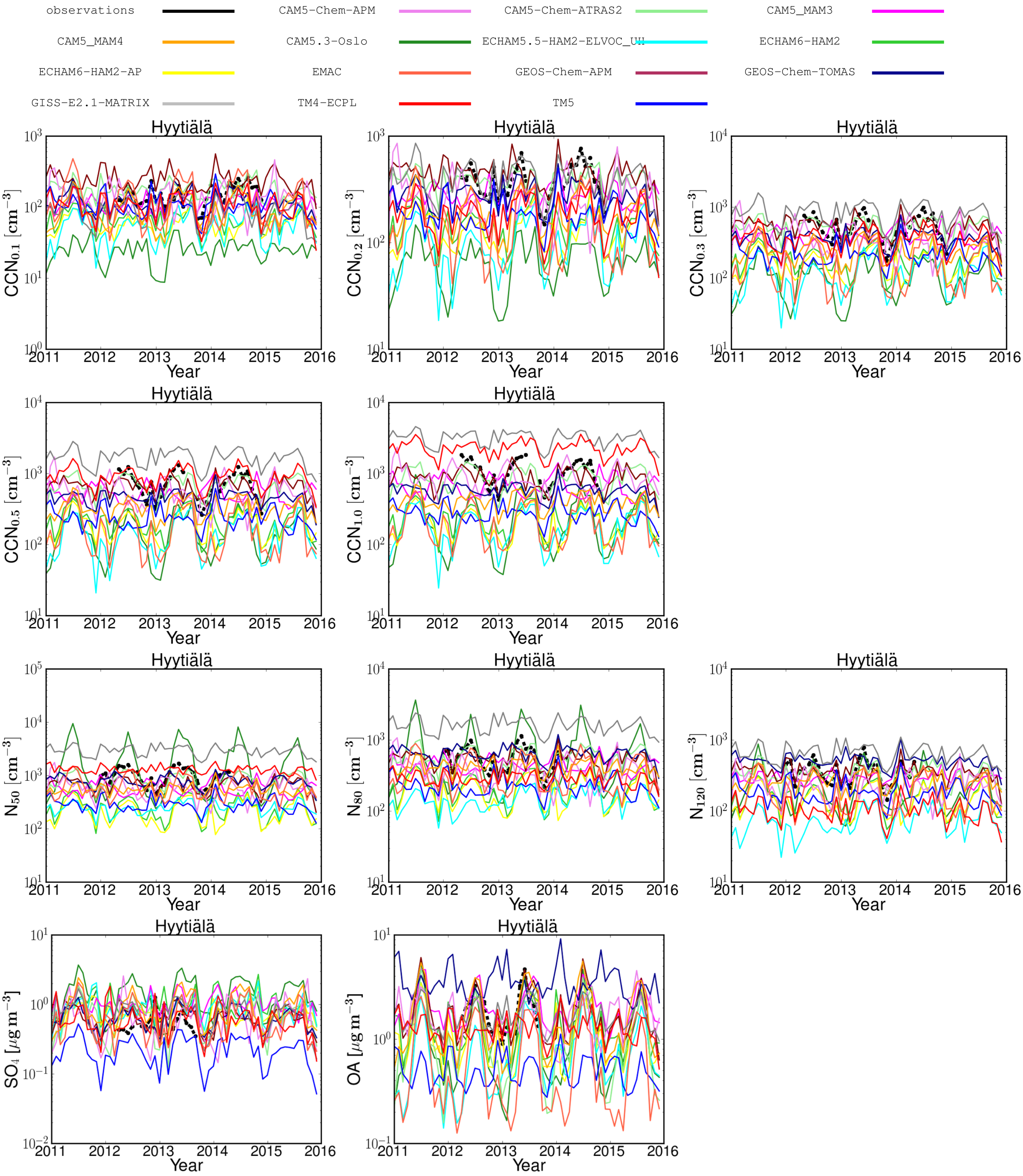

Figure S2 (cont 9/9)

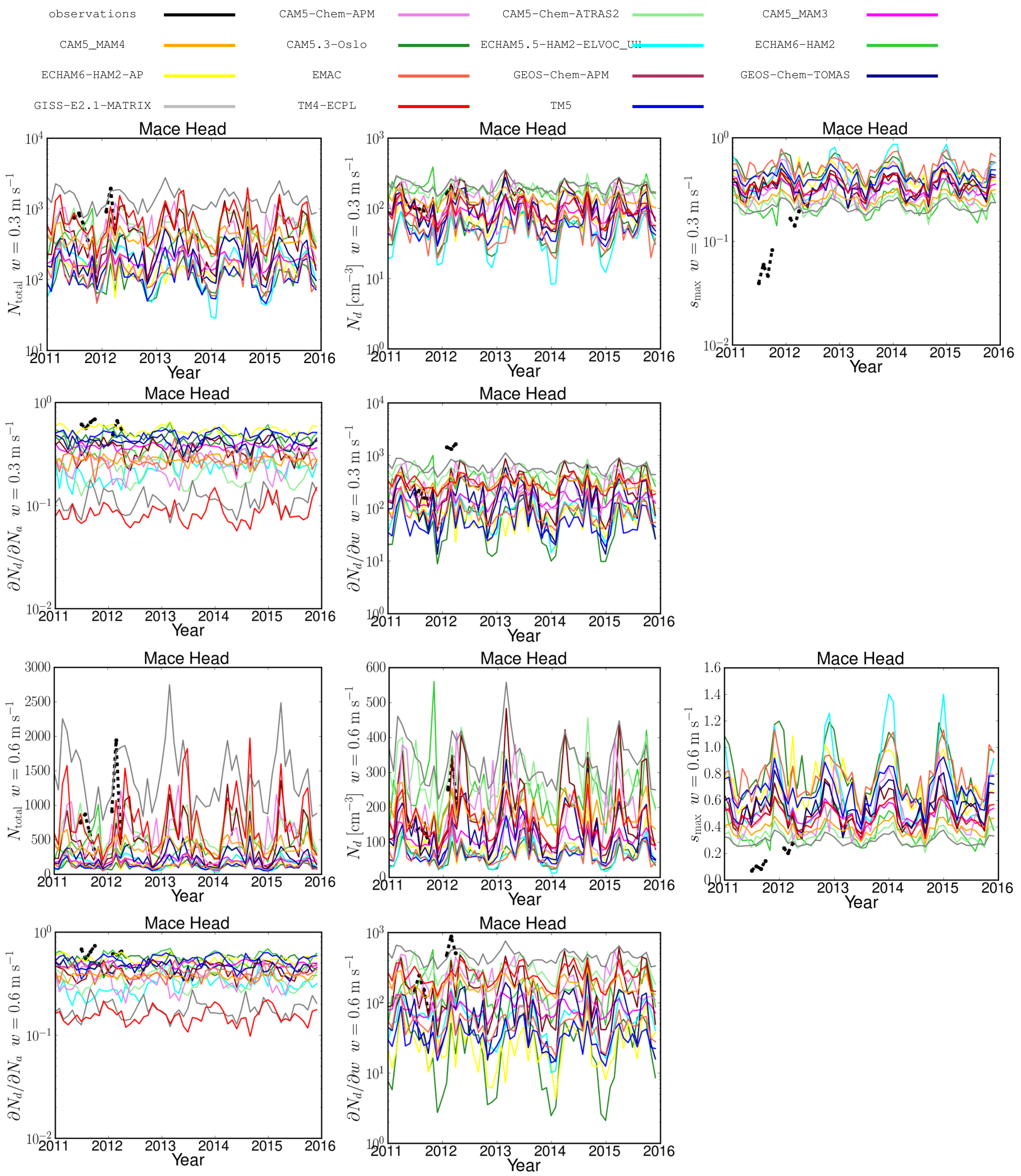

Figure S3: Comparison between monthly averages of the cloud droplet properties from observations (black circles) and from the individual models (see figure legend for model identification). Figures are drawn per station and for two different updraft velocities (w=0.3 ms^{-1} and w=0.6 ms^{-1}) marked on the y-axis. For each station and updraft velocity the five graphs show (as indicated in the y-axis label), the total number of particles, N_t, the number of cloud droplets, N_d, the maximum supersaturation, s_{max} (in %), the sensitivity of the N_d to the total number of aerosol particles, ($\partial N_d/\partial N_a$), and the sensitivity of the N_d to the wind speed, ($\partial N_d/\partial w$).

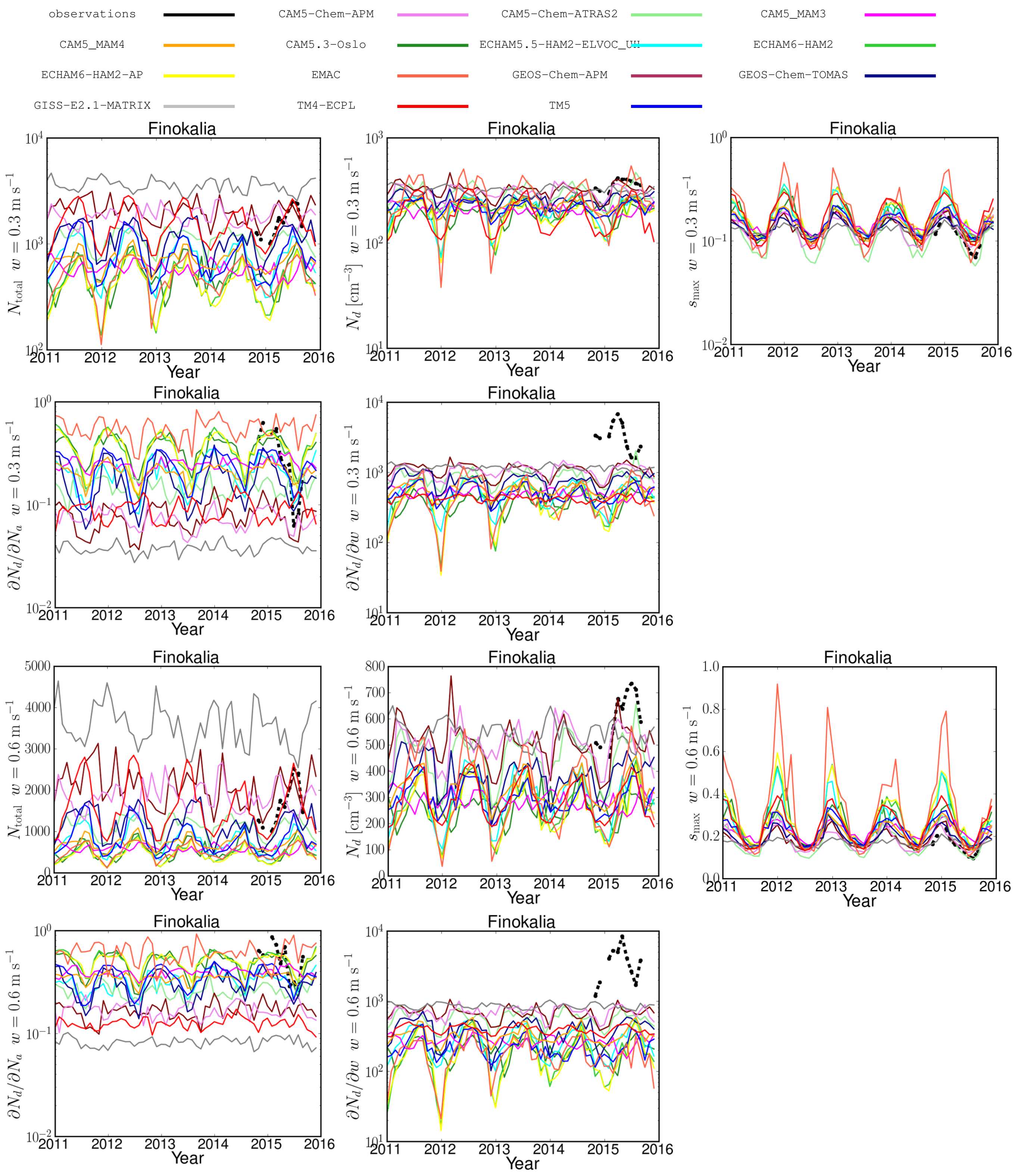

Figure S3 (cont 2/5)

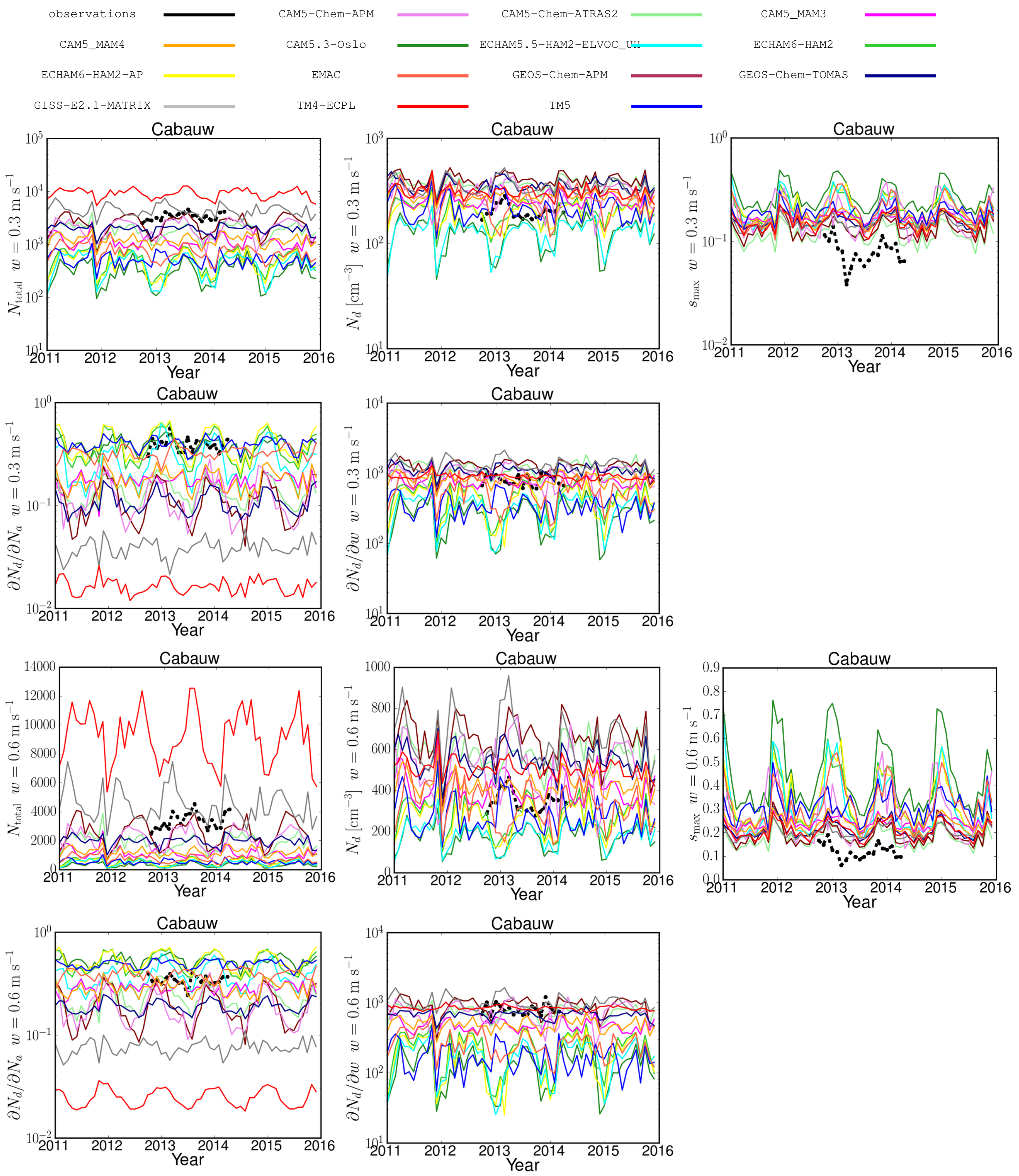

Figure S3 (cont 3/5)

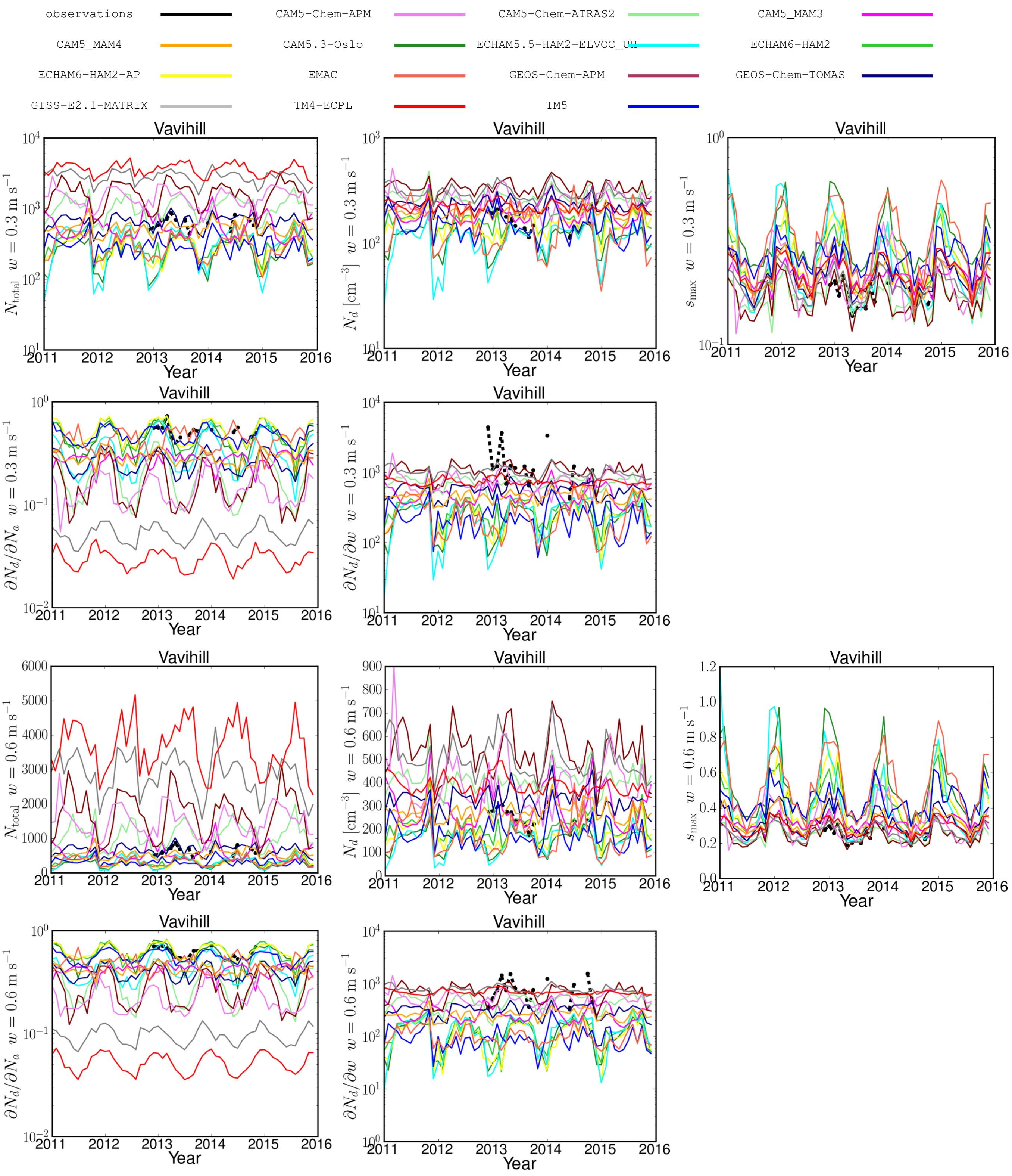

Figure S3 (cont 4/5)

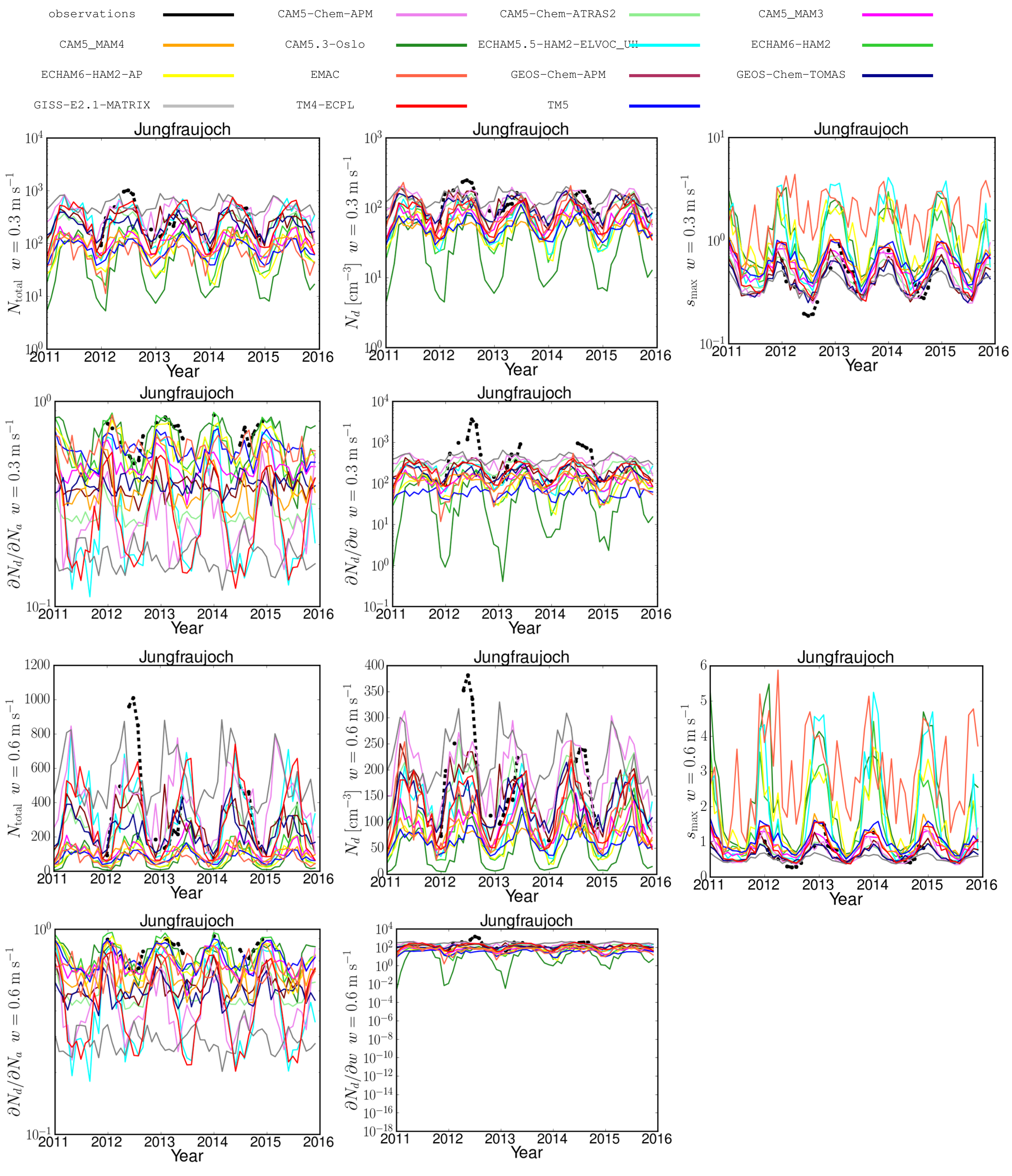

Figure S3 (cont 5/5)

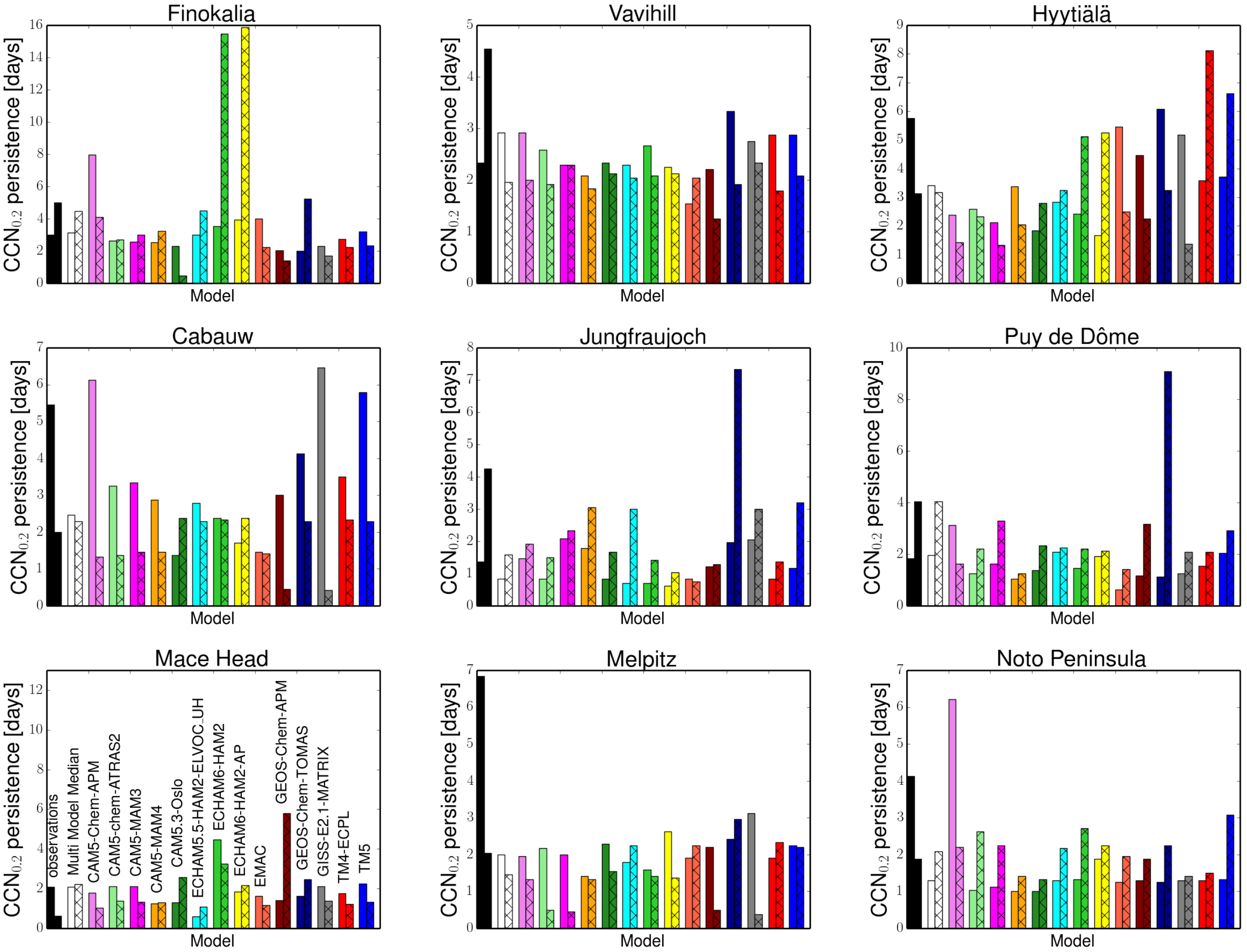

Figure-S4: Comparison between the persistence times derived from the observations (in black bars) and from the model results of $CCN_{0.2}$ during winter and summer for each station. Each pair of bars that follows corresponds to the predictions of each model for winter (left bar) and summer (right shaded bar), respectively. The white bars show the persistence times of the MMM. The persistence times derived from model simulations have been computed at the same time periods as those derived from the observations.

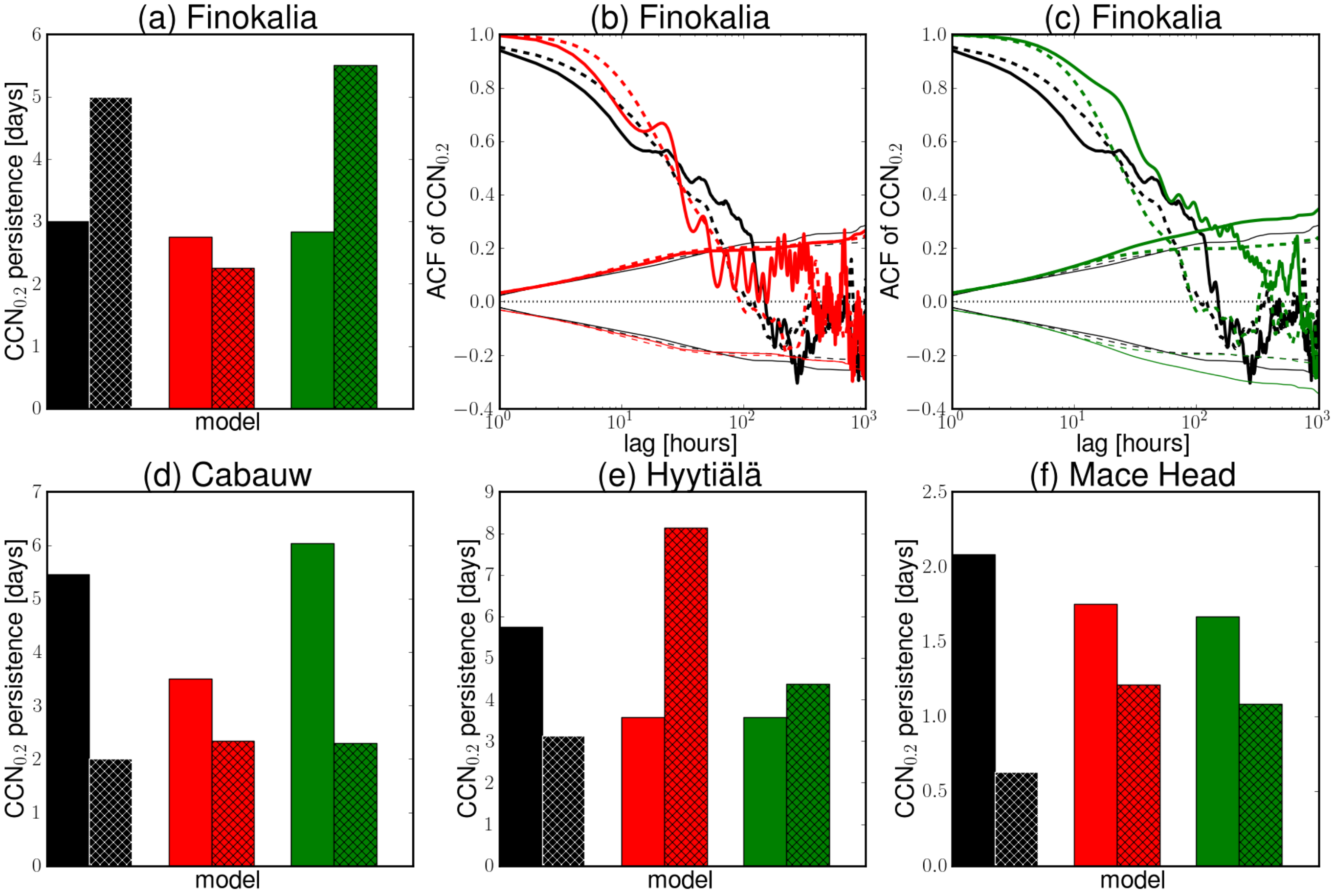

Figure-S5: (a) Comparison of the winter and summer persistence times of $CCN_{0.2}$ for Finokalia station calculated using observational data (black bars) and the results of TM4-ECPL model for the base simulation (red bars) and the sensitivity simulation assuming the same emission of carbonaceous aerosols with the base case but fewer particles of larger size (green bars). The pairs of columns show persistences first for winter and second for summer. In both simulations a log-normal distribution for the size distribution of emitted particles with a geometric standard deviation $\sigma = 1.59$ has been assumed/used. In the base simulation, the dry median diameters of the emitted particles coming from fossil fuel combustion and from vegetation fires are 30 nm and 80 nm, respectively, while in the sensitivity simulations all particles are emitted at 60 nm. Left bars are for winter and right shaded bars are for summer. (b) Autocorrelation function (ACF) of the $CCN_{0.2}$ for Finokalia station calculated for summer (continuous lines) and for winter (dashed lines). Lines in colors are for the bars. The large-lag standard deviation curves are also shown in the graphs. The persistence time is defined as the time that the large-lag standard error crosses the ACF curve (Schmale et al., 2018). (c) same as panel-b using the results of the sensitivity simulation. (d)-(f) same as the panel-a for Cabauw, Hyytiälä and Mace Head stations, respectively.

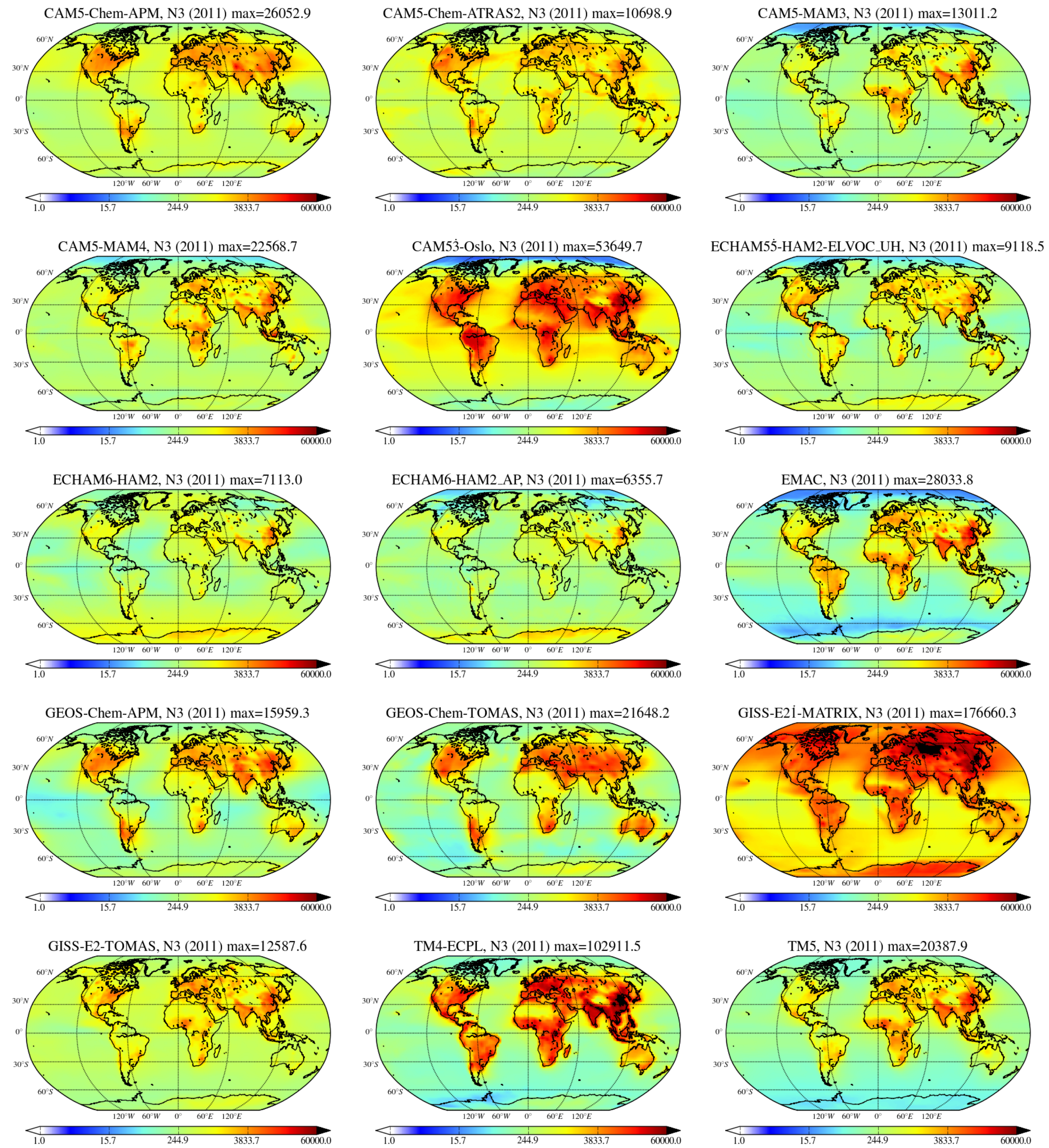

Figure-S6: Global surface distribution for the year 2011 of the N_3 number concentrations as computed by the fifteen models participated in this study. At the top of each panel the maximum value of the N_3 simulated by the model is denoted. Units are number of particles cm^{-3}.

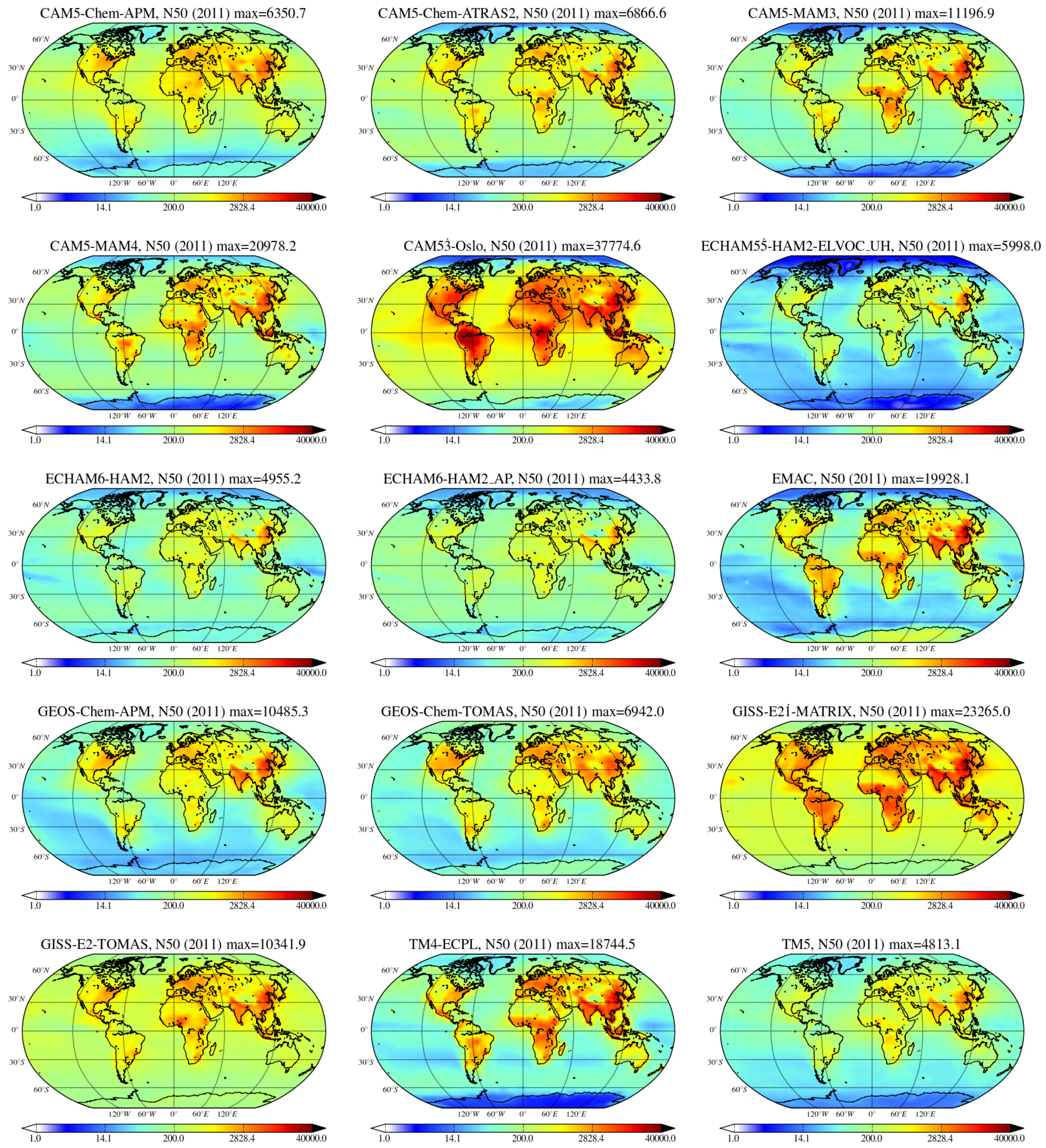

Figure-S7: Same as Figure-S6 for N_{50} number concentrations. Units are number of particles cm^{-3}.

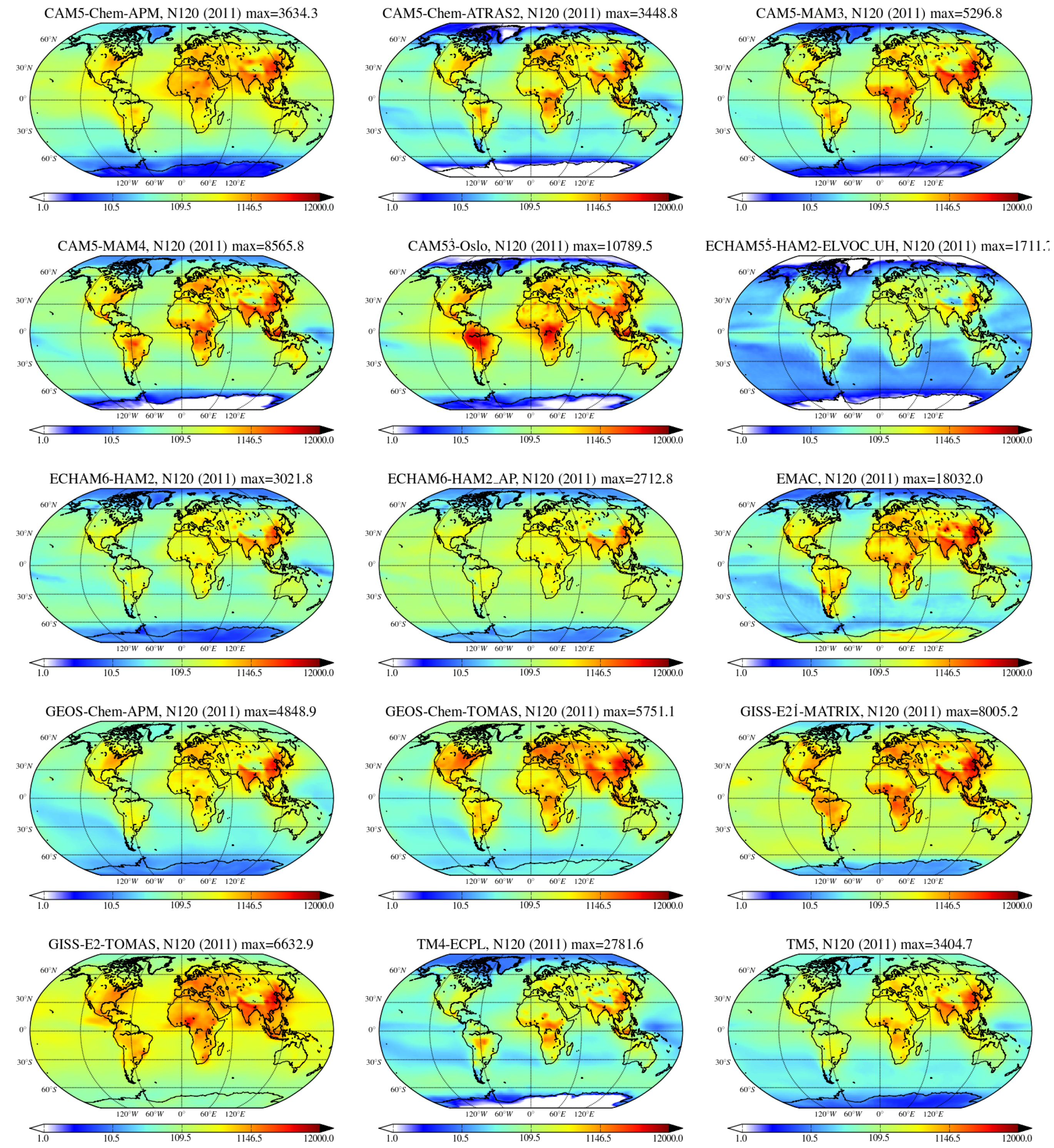

Figure-S8: Same as Figure-S6 for N_{120} number concentrations. Units are number of particles cm^{-3}.

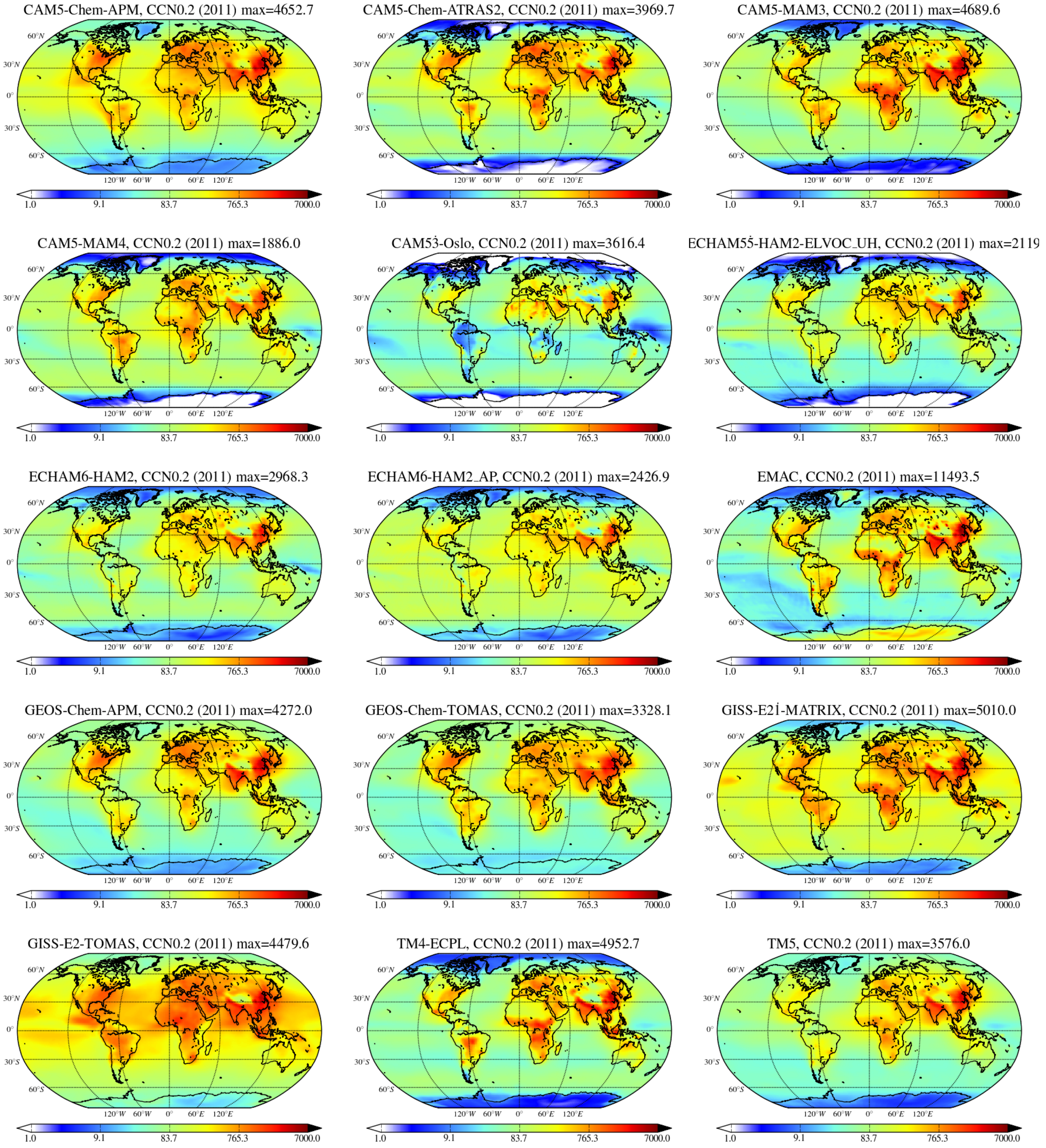

Figure-S9: Same as Figure-S6 for $CCN_{0.2}$ number concentrations. Units are number of particles cm^{-3}.

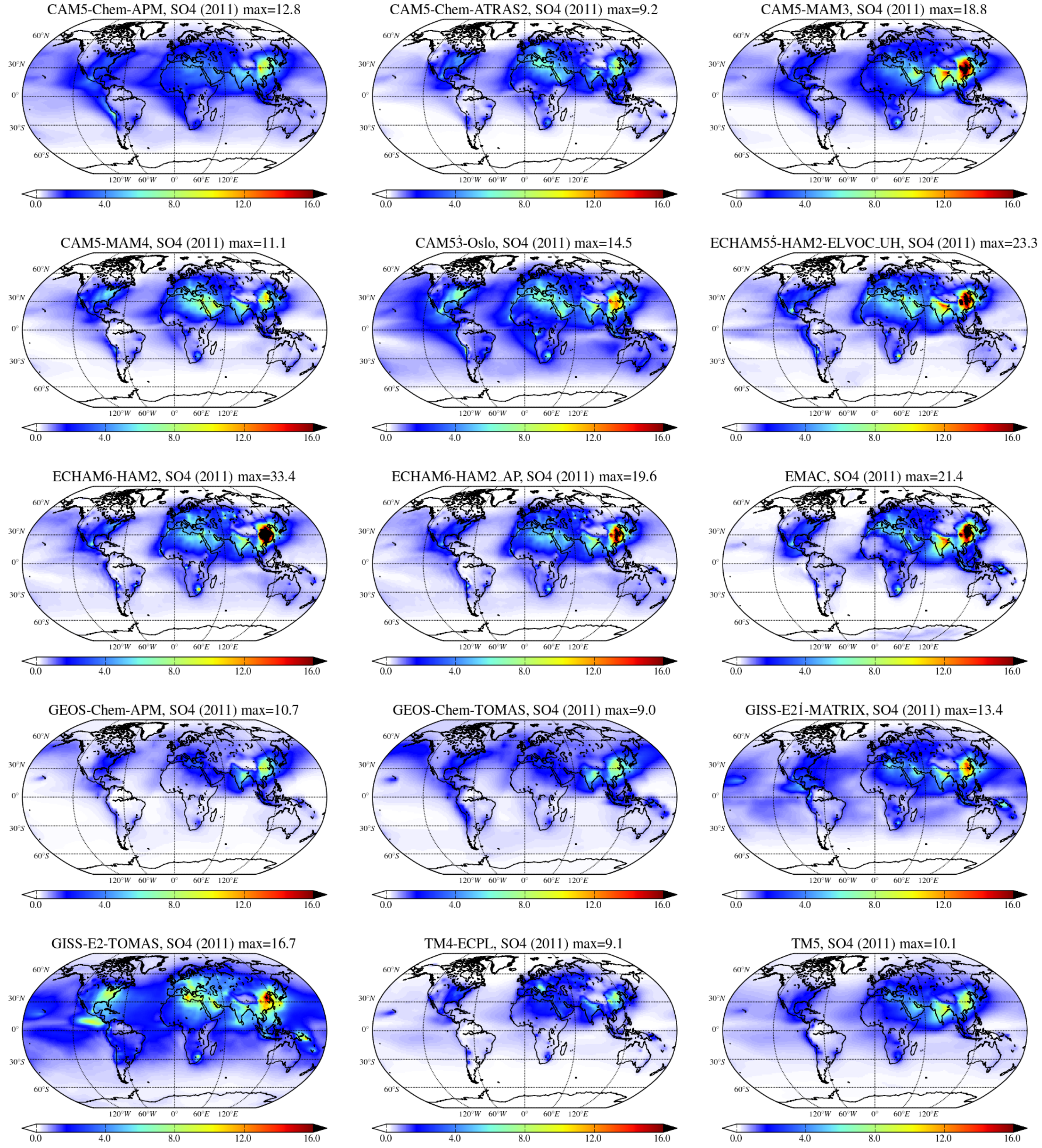

Figure-S10: Global surface distribution of the mass concentration of sulfate (SO4) of PM_1 particles as computed by all models. At the top of each map the maximum value of the SO4 is denoted. Units are μg-SO4 m^{-3}.

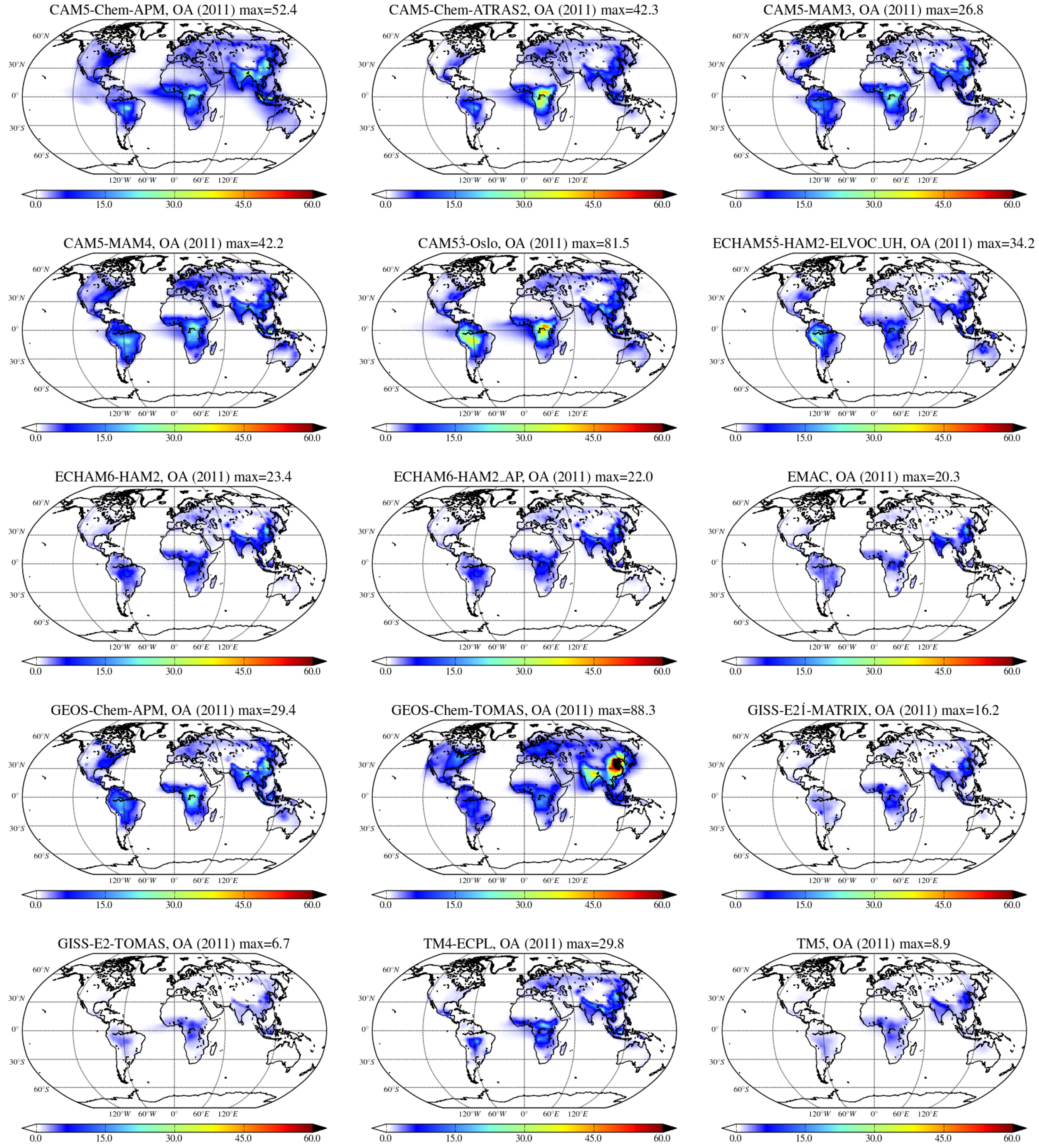

Figure-S11: Same as Figure-S10 for OA. Units are μg-OA m^{-3}.

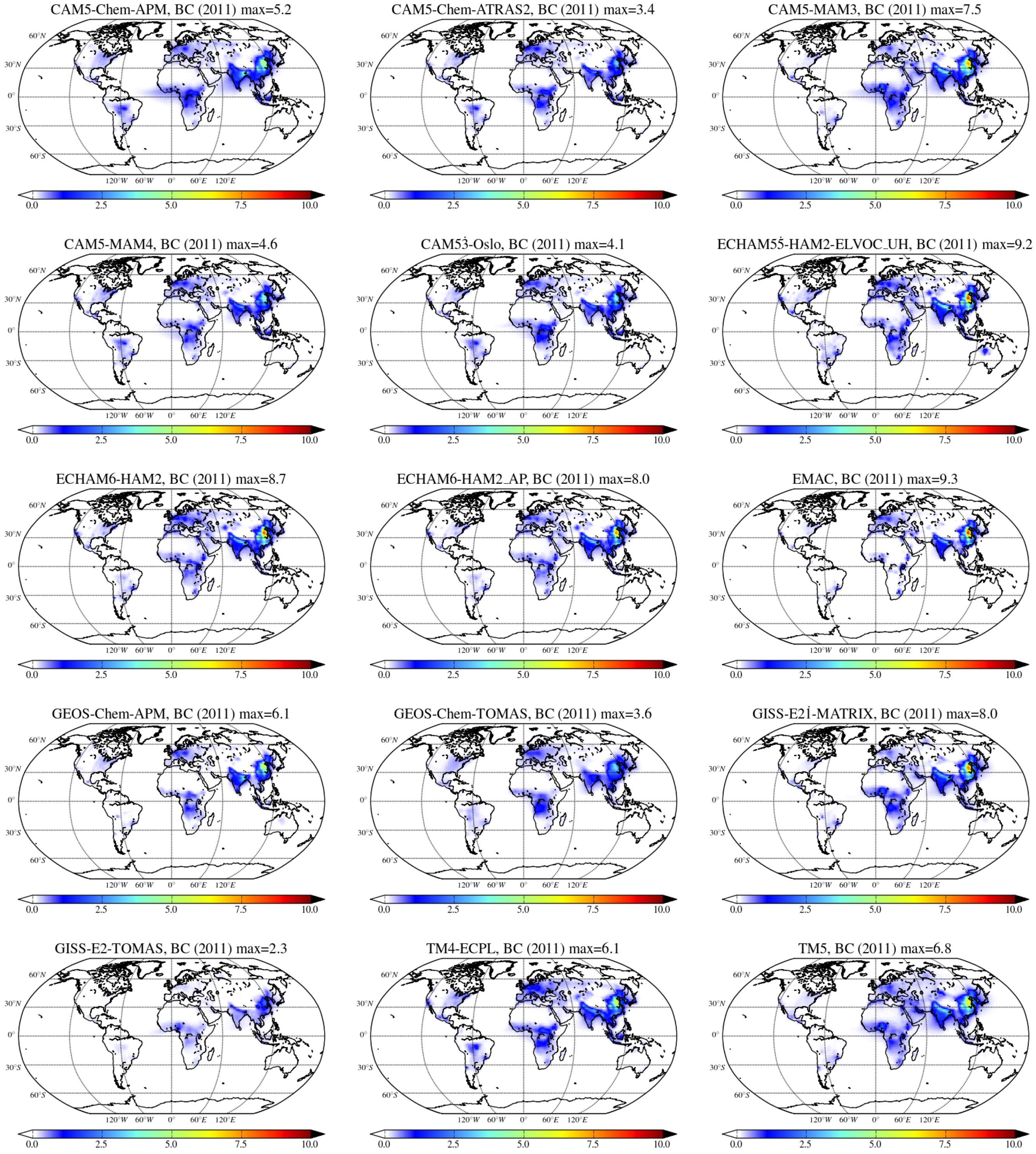

Figure-S12: Same as Figure-S10 for BC. Units are $\mu g\, m^{-3}$.

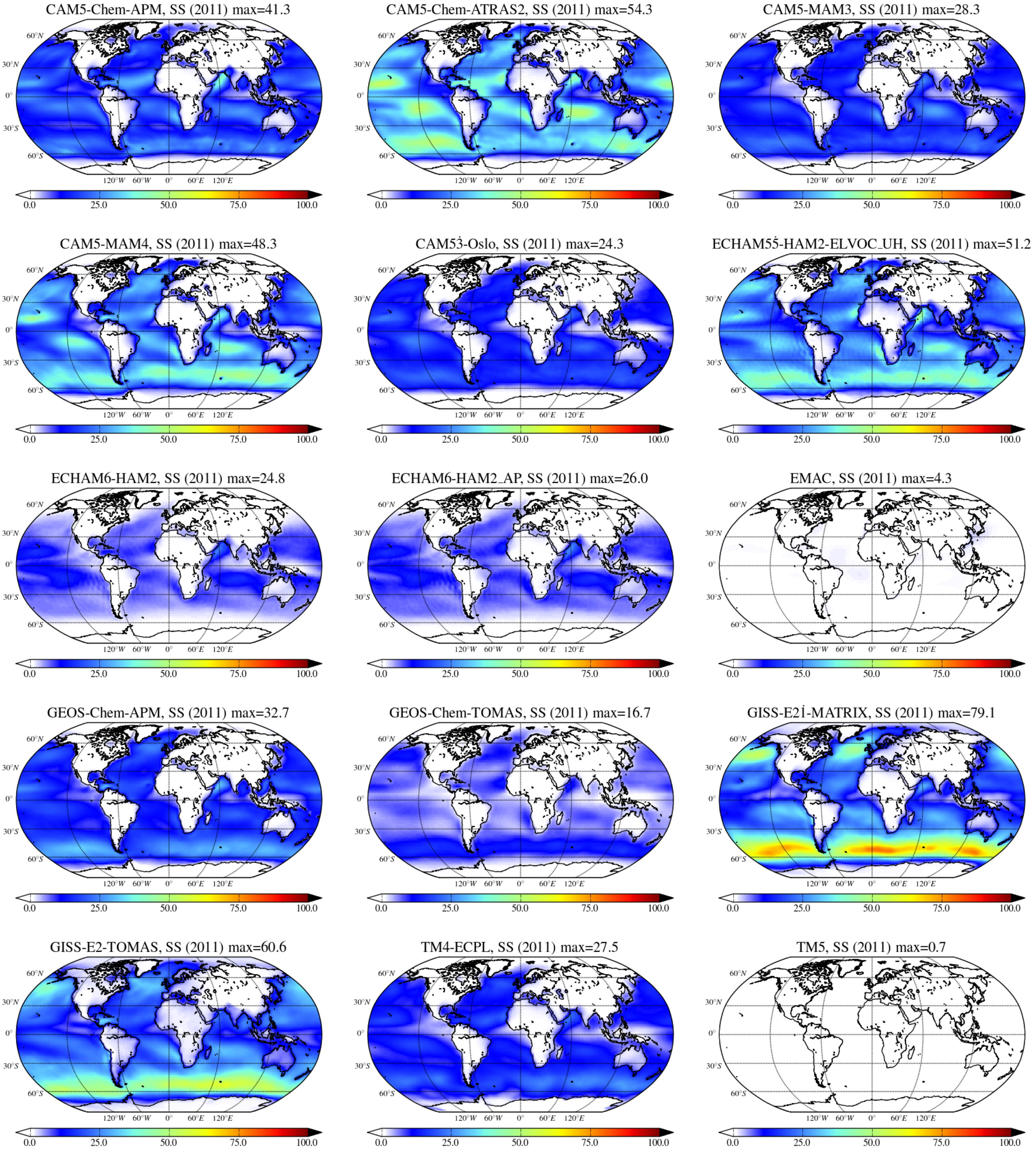

Figure-S13: Same as Figure-S10 for SS. Units are $\mu g\, m^{-3}$.

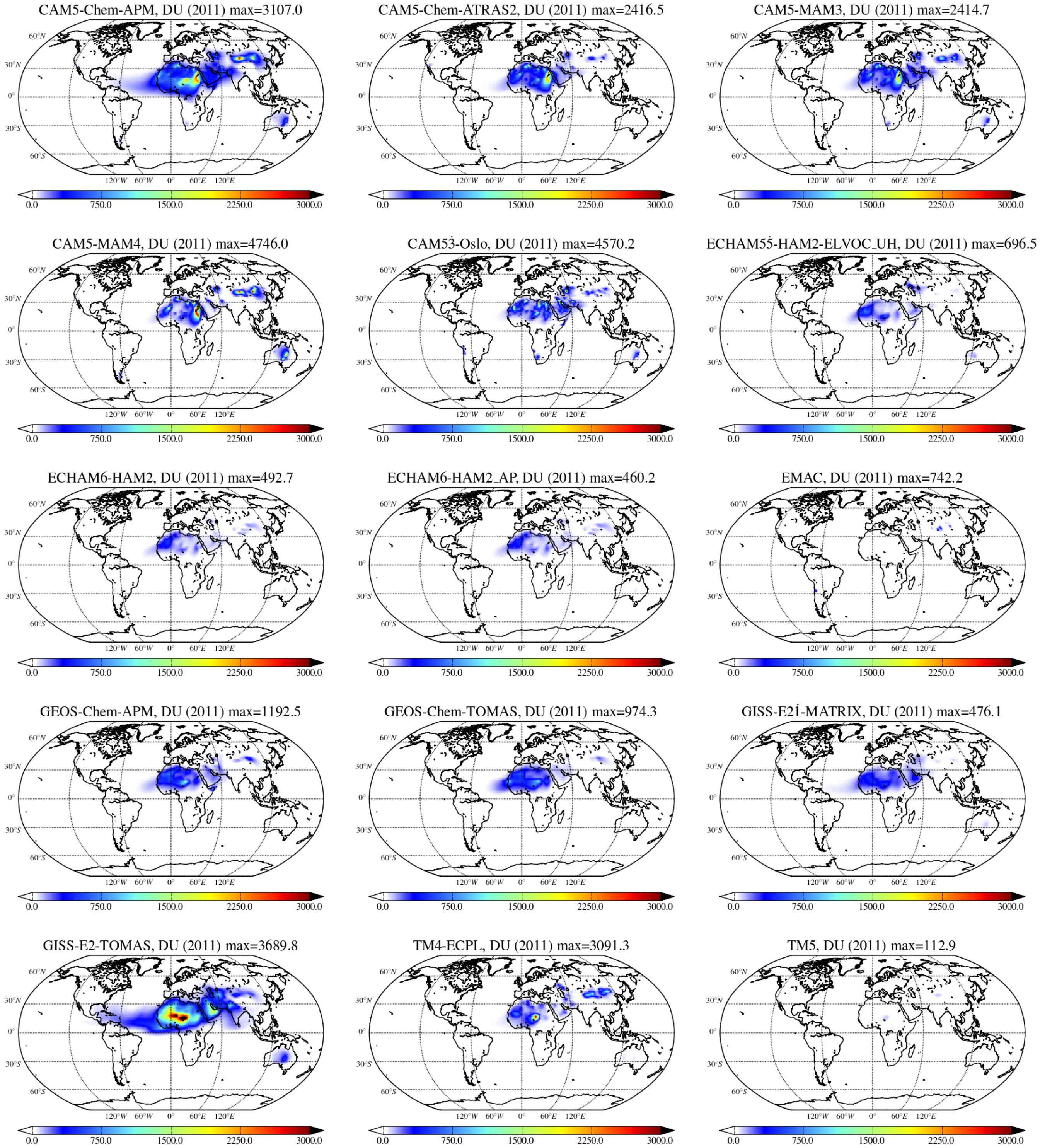

Figure-S14: Same as Figure-S10 for DU. Units are $\mu g\, m^{-3}$.

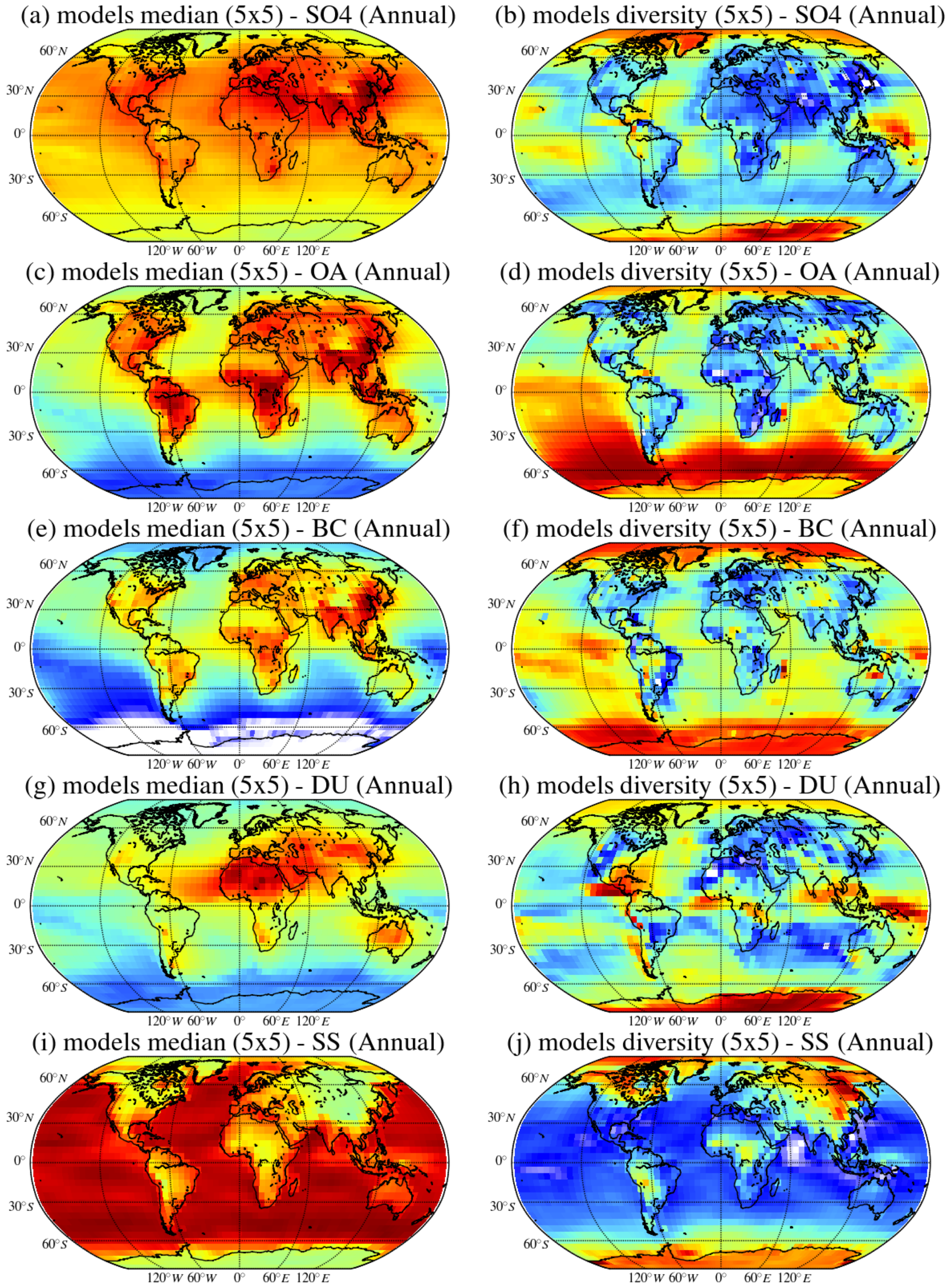

Figure-S15: Global distributions of the annual multi-model median concentrations of the SO4, OA, BC, DU and SS (from top to bottom) for the year 2011 (left column) and the corresponding diversities (right column). Model diversities are calculated as the ratio of the standard deviation to the mean of the models.

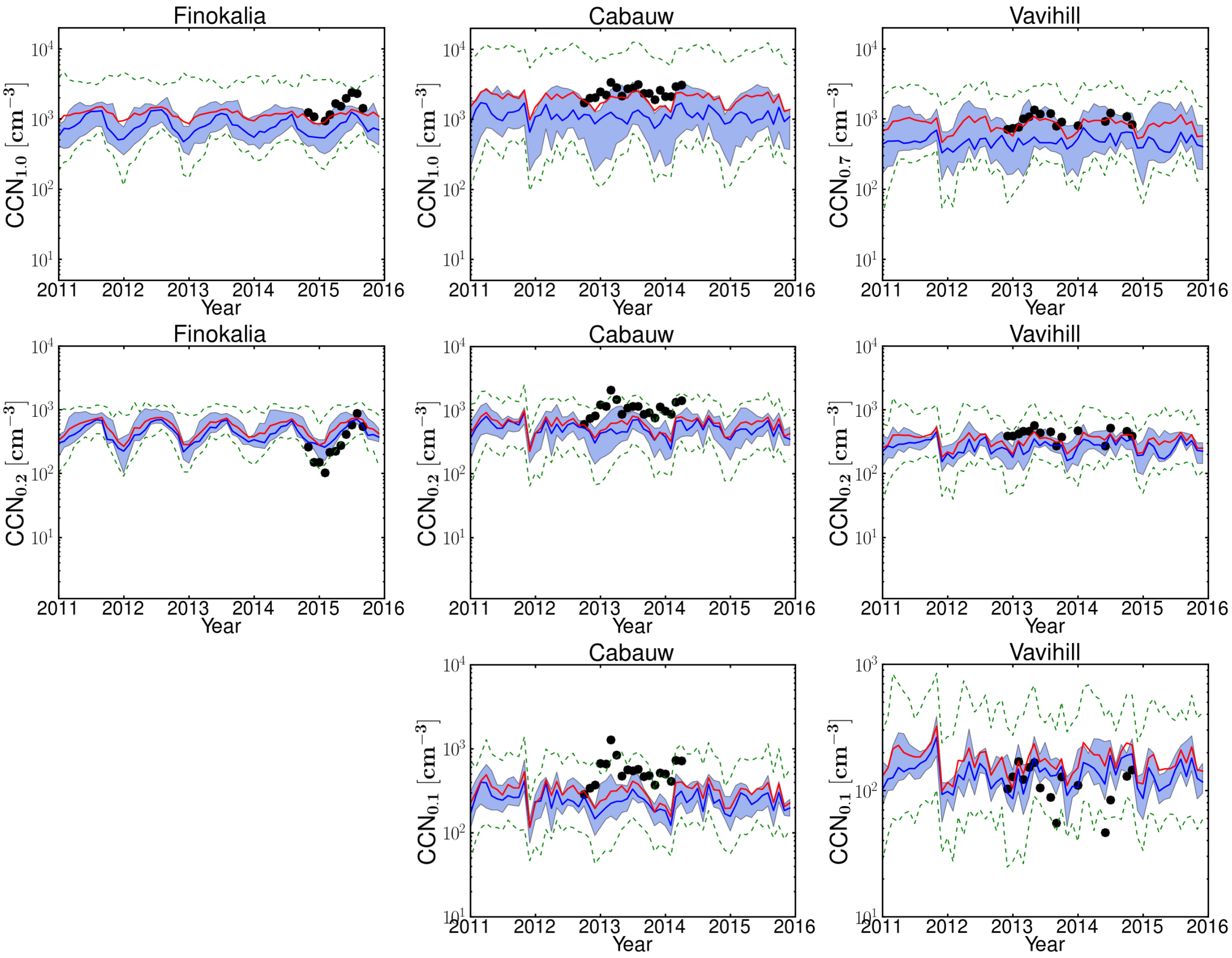

Figure-S16: Monthly ensembles for the years 2011-2015 of the CCN number concentration for supersaturation 0.2% ($CCN_{0.2}$), 0.1% ($CCN_{0.1}$), 0.7% ($CCN_{0.7}$) and 1.0% ($CCN_{1.0}$) when observational data are available for Finokalia, Cabauw and Vavihill.

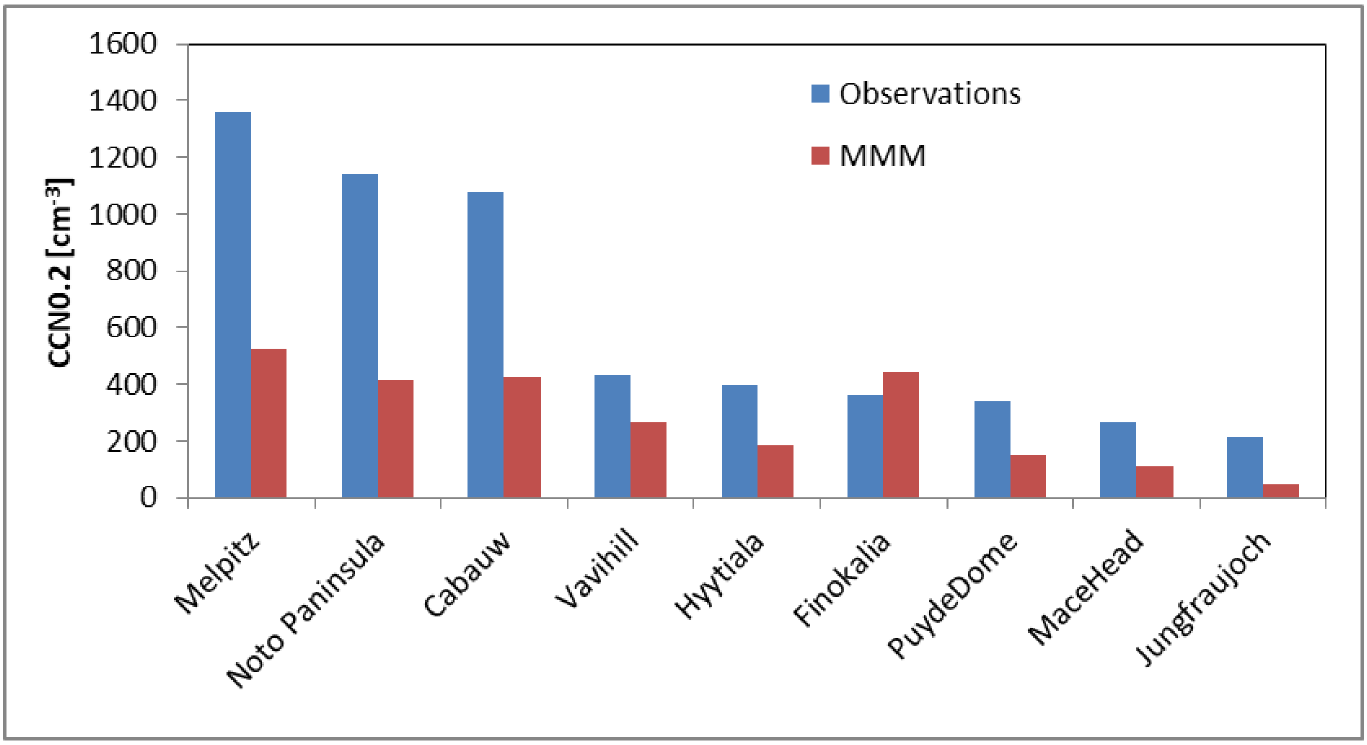

Figure-S17: Mean $CCN_{0.2}$ as calculated from the observations and as computed from the daily MMM for the days with available observations. The stations have been ranked based on $CCN_{0.2}$ observations in decreasing levels.

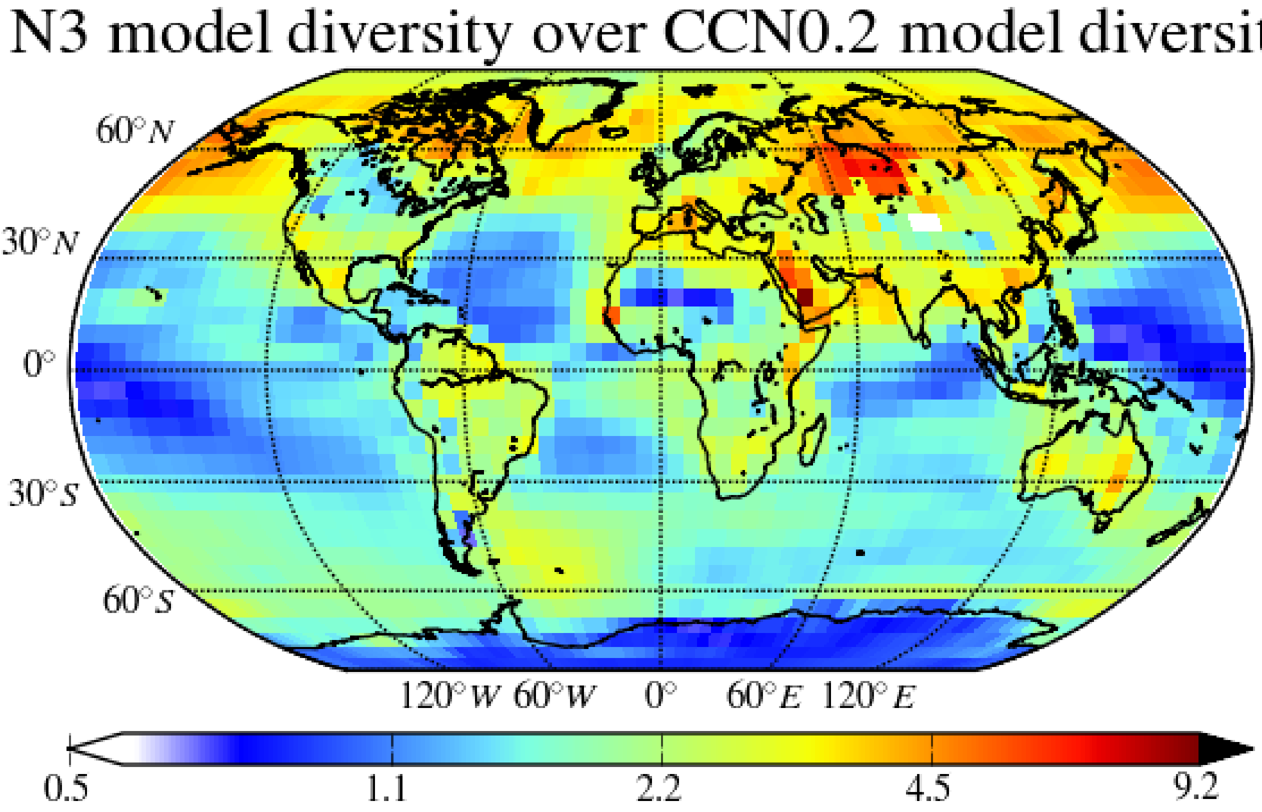

Figure-S18: Ratio of the model diversity of N_3 (Figure 10b) to that of $CCN_{0.2}$ (Figure 10f)
